# A vesicular stomatitis virus-based prime-boost vaccination strategy induces potent and protective neutralizing antibodies against SARS-CoV-2

**DOI:** 10.1371/journal.ppat.1010092

**Published:** 2021-12-16

**Authors:** Gyoung Nyoun Kim, Jung-ah Choi, Kunyu Wu, Nasrin Saeedian, Eunji Yang, Hayan Park, Sun-Je Woo, Gippeum Lim, Seong-Gyu Kim, Su-Kyeong Eo, Hoe Won Jeong, Taewoo Kim, Jae-Hyung Chang, Sang Hwan Seo, Na Hyung Kim, Eunsil Choi, Seungho Choo, Sangkyun Lee, Andrew Winterborn, Yue Li, Kate Parham, Justin M. Donovan, Brock Fenton, Jimmy D. Dikeakos, Gregory A. Dekaban, S. M. Mansour Haeryfar, Ryan M. Troyer, Eric J. Arts, Stephen D. Barr, Manki Song, C. Yong Kang

**Affiliations:** 1 Department of Microbiology and Immunology, Schulich School of Medicine and Dentistry, The University of Western Ontario, London, Ontario, Canada; 2 International Vaccine Institute, SNU Research Park, 1 Gwanak-ro, Gwanak-gu, Seoul, Korea; 3 Sumagen, 4F Dongwon Bldg, Teheran-ro 77-gil, Gangnam-gu, Seoul, Korea; 4 Animal Facility, Queen’s University, Kinston, Ontario, Canada; 5 Department of Biology, Faculty of Science, The University of Western Ontario, London, Ontario, Canada; 6 Molecular Medicine Research Laboratories, Robarts Research Institute, University of Western Ontario, London, Ontario, Canada; Chang Gung University, TAIWAN

## Abstract

The development of safe and effective vaccines to prevent SARS-CoV-2 infections remains an urgent priority worldwide. We have used a recombinant vesicular stomatitis virus (rVSV)-based prime-boost immunization strategy to develop an effective COVID-19 vaccine candidate. We have constructed VSV genomes carrying exogenous genes resulting in the production of avirulent rVSV carrying the full-length spike protein (S_F_), the S1 subunit, or the receptor-binding domain (RBD) plus envelope (E) protein of SARS-CoV-2. Adding the honeybee melittin signal peptide (msp) to the N-terminus enhanced the protein expression, and adding the VSV G protein transmembrane domain and the cytoplasmic tail (Gtc) enhanced protein incorporation into pseudotype VSV. All rVSVs expressed three different forms of SARS-CoV-2 spike proteins, but chimeras with VSV-Gtc demonstrated the highest rVSV-associated expression. In immunized mice, rVSV with chimeric S protein-Gtc derivatives induced the highest level of potent neutralizing antibodies and T cell responses, and rVSV harboring the full-length msp-S_F_-Gtc proved to be the superior immunogen. More importantly, rVSV-msp-S_F_-Gtc vaccinated animals were completely protected from a subsequent SARS-CoV-2 challenge. Overall, we have developed an efficient strategy to induce a protective response in SARS-CoV-2 challenged immunized mice. Vaccination with our rVSV-based vector may be an effective solution in the global fight against COVID-19.

## Author summary

The COVID-19 pandemic has had unprecedented global health, economic and societal impact globally. Vaccinating the majority of the world’s population is the best way to help prevent new infections. Many vaccines have been developed to prevent various viral diseases that are currently in use around the world. This has generated a high demand for these vaccines, putting a strain on production capacity and delivery. With new variants of concern starting to dominate the human pandemic, new derivatives of the current vaccines may be necessary for continued protection from SARS-CoV-2 infection. We have developed a vaccine that uses a safe vesicular stomatitis virus-based delivery vehicle to present a key SARS-CoV-2 protein to our immune system in order to train it to recognize and prevent SARS-CoV-2 infection. Our vaccine completely protected vaccinated animals from SARS-CoV-2 infection and significantly reduced lung damage, a major hallmark of COVID-19.

## Introduction

Severe acute respiratory syndrome coronavirus 2 (SARS-CoV-2) causes coronavirus disease 2019 (COVID-19) and, as of October 27, 2021, had infected over 244 million people and caused over 4.8 million deaths. The global pandemic has resulted in massive societal and economic disruption worldwide [1]. SARS-CoV-2 belongs to the *Betacoronavirus* genus which includes recently emerged human pathogenic coronaviruses SARS-CoV and MERS-CoV. As a respiratory pathogen, SARS-CoV-2 primarily targets the lungs in humans. However, it also targets other organs such as the heart, liver and kidneys. As a result, SARS-CoV-2 infection can lead to multiple organ failure, shock, acute respiratory distress syndrome, heart failure, arrhythmias, and renal failure [2,3].

The ability of SARS-CoV-2 to infect humans is largely due to its envelope spike protein using the human angiotensin-converting enzyme 2 (hACE2) for cell entry [4,5]. The spike protein is cleaved into S1 and S2 subunits and entry is initiated by binding of the receptor-binding domain (RBD) within the S1 subunit to hACE2 on the cell surface. The S2 subunit mediates viral envelope fusion with the host cell membrane. The full-length spike protein (S_F_) induces neutralizing antibodies and cell-mediated immune responses, making it a favorable target antigen for vaccine development [6–9]. Several different vaccine platforms have used the S_F_ to induce immunity in humans. Phase 3 clinical trials have shown varying degrees of protection from COVID-19 disease in humans by presenting the S_F_ to induce immunity using different vaccine platforms. Two experimental mRNA vaccines, one from Pfizer/BioNTech and another from Moderna, have shown the highest efficacy to date (95% and 94% respectively) [10,11]. Oxford–AstraZeneca’s chimpanzee adenovirus vector-based vaccine trial has shown a 62.1%-90% efficacy depending on the dose of the priming vaccination [12]. With high global demand for COVID-19 vaccines, it has been challenging to maintain the current production capacity. This challenge, together with others such as delivery, vaccine hesitancy, and unknown longevity of protection highlights the need for additional vaccine strategies. An estimated two-thirds to three-quarters of global population will likely receive viral vector vaccines for SARS-CoV-2 prevention due to reduced cost of production and delivery compared to mRNA vaccines. If new variants of concern dominate the human pandemic in the future, new derivatives of the current vaccines may be necessary for continued SARS-CoV-2 protection and a concomitant block in viral spread. However, anti-vector immunity induced with the prime-boost delivery of the adenovirus-based vaccines such as those produced by Oxford–AstraZeneca and Johnson & Johnson may prevent reuse of these viral vectors for vaccination for new SARS-CoV-2 variants of concern in the future. Thus, SARS-CoV-2 vaccine development and testing must continue, and inexpensive viral vector-based vaccines, such as the rVSV-SARS-CoV-2 described herein, may be critical for future pandemic control.

Vesicular stomatitis virus (VSV) was developed as a live viral vaccine vector for Ebola virus infections and is in pre-clinical trials for other viruses including SARS-CoV-2, HIV-1, hantaviruses, arenaviruses, and influenza viruses [13–19]. For increased vaccine safety, we previously introduced three mutations into the *matrix* (*M*) gene of these vectors that resulted in reduced vector cytopathogenicity [20]. The recombinant VSV-ΔG expressing SARS-CoV-2 spike protein vaccine developed by Yahalom-Ronen [19], similar to Merck’s Ebola virus vaccine, showed protection against the SARS-CoV-2 challenge in Syrian hamsters. This approach was abandoned due to insufficient VSV vector production mediated by entry through the SARS-CoV-2 spike protein, an observation we have also described herein. Our VSV approach involves non-pathogenic vector production through VSV G-mediated entry and through the introduction of three mutations into the *matrix* (*M*) gene that drastically reduced vector cytopathogenicity [20]. We previously developed a recombinant vesicular stomatitis virus vaccine platform to prime with the Indiana serotype (rVSV_Ind_) and to boost with the New Jersey serotype (rVSV_NJ_) (or vice versa). This induced robust adaptive immune responses against the inserted gene products [20,21]. Antibodies raised against one serotype of rVSV did not neutralize the other serotype [22]. Therefore, the impact of anti-vector antibodies arising after the priming immunization was avoided or minimized, resulting in a stronger immune response to the inserted immunogen. The attenuated and replication-competent nature of the rVSV vaccine vector allows for lower levels of vaccine needed per vaccination compared to replication-incompetent vaccine vectors and thus, decreases manufacturing costs and capacity [20]. Similar viral vectors have demonstrated an excellent safety profile in human trials (reviewed in [23]).

Herein, we developed and tested multiple rVSV vaccine candidates using different SARS-CoV-2 antigens. In an attempt to induce potent and long-lasting protection against SARS-CoV-2 infection, we developed rVSVs expressing the full-length spike (S_F_) protein, S1 subunit, and receptor-binding domain of SARS-CoV-2 spike protein. These proteins were expressed in *cis* with or without the honeybee melittin signal peptide (msp), at the N-terminus to increase the efficiency of spike protein synthesis, glycosylation and intracellular trafficking, as well as with or without the transmembrane domain and cytoplasmic tail (Gtc) of VSV G protein at the C-terminus to enhance the incorporation of the spike protein into pseudotype VSV [24]. We found that prime-boost vaccination of mice with dual rVSV serotypes carrying modified SARS-CoV-2 S_F_ antigens induced robust neutralizing antibodies and cell-mediated immune responses against wild-type SARS-CoV-2. Furthermore, vaccination resulted in complete survival of hACE2 transgenic mice challenged with SARS-CoV-2, limited weight loss, lack of infectious virus recovery from the lungs, and significantly reduced pathology in lung tissue.

## Results

### Efficient expression of SARS-CoV-2 antigens in rVSV_Ind_ and rVSV_NJ_ infected cells

**Fig 1:**
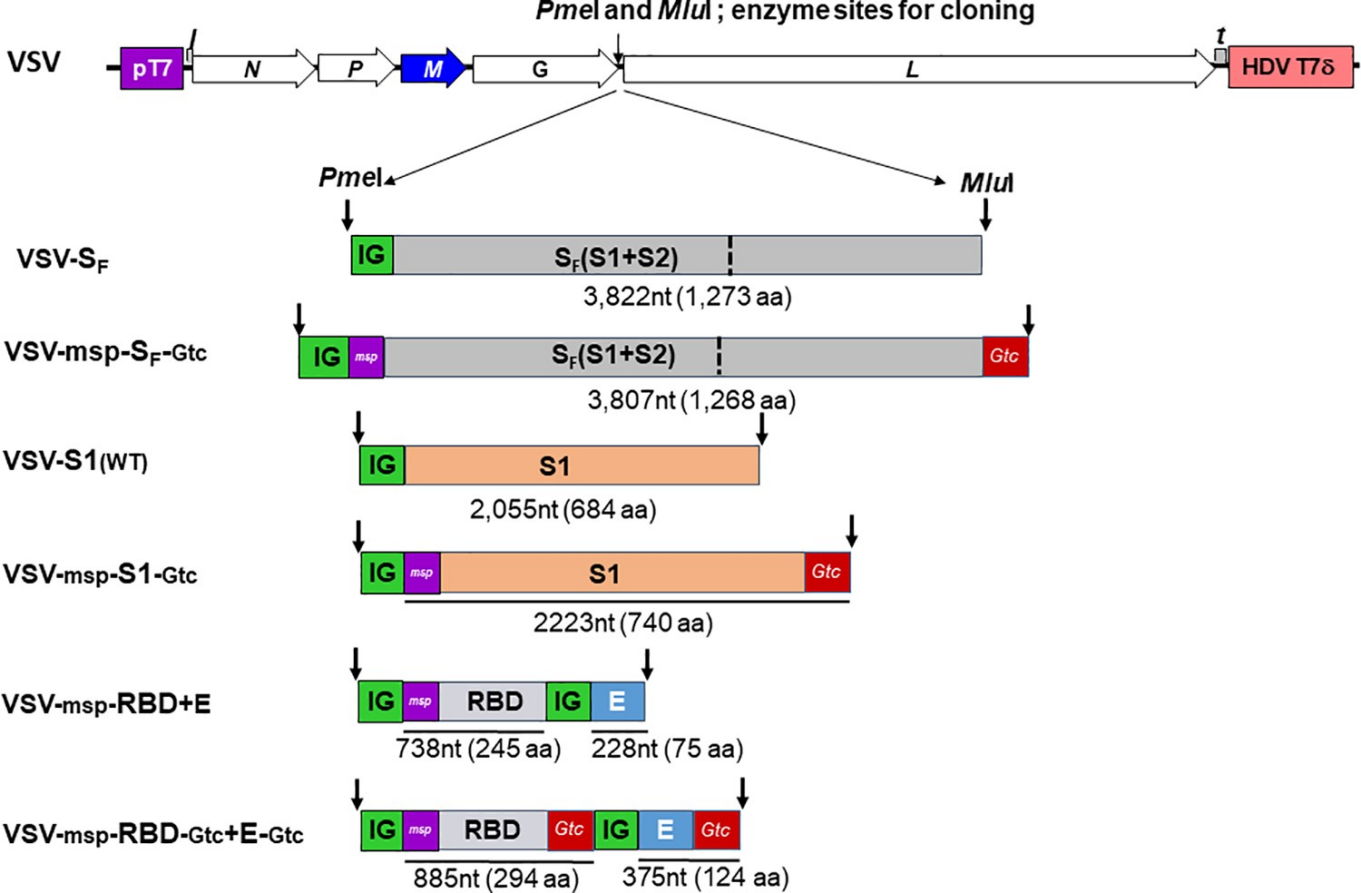
Construction of recombinant rVSV_Ind_ and rVSV_NJ_ with S_F_, S1, and RBD+E genes of SARS-CoV-2 with and without honeybee *msp* and VSV *Gtc*. Codon-optimized full-length Spike protein gene (S_F_), S1 subunit gene and the receptor-binding domain (RBD) plus envelope protein genes of SARS-CoV-2 with and without 21 amino acids honeybee melittin signal peptide [(msp) NH_2_-MKFLVNVALVFMVVYISYIYA-COOH] [24] gene in the purple box, and 49 amino acids VSV G protein transmembrane domain and cytoplasmic tail [(Gtc) NH_2_-SSIASFFFIIGLIIGLFL VLRVGIYLCIKLKHTKKRQIYTDIEMNRLGK-COOH] gene in the red box were inserted into the G and L gene junction of rVSV_Ind_ and rVSV_NJ_. In addition, 25- nucleotides-long VSV intergenic junctions (5´-CATATGAAAAAAACTAACAGATATC-3´), in the green box, were inserted between genes to provide transcription termination, polyadenylation and the transcription reinitiation sequences. Recombinant viruses were rescued by VSV reverse genetics [20]. pT7: Bacteriophage T7 promoter for DNA-dependent RNA polymerase. *N*: VSV Nucleocapsid Protein gene. *P*: VSV Phosphoprotein gene. *M*: VSV Matrix protein gene. *G*: VSV Glycoprotein gene. *L*: VSV Large protein, RNA-dependent RNA polymerase gene. *l*: Leader region in the 3´-end of the VSV genome. *t*: Trailer region in the 5´-end of the VSV genome. HDV: Hepatitis delta virus ribozyme encoding sequences. T7δ: Bacteriophage T7 transcriptional terminator sequences. nt: nucleotides. aa: amino acids.

**Fig 2:**
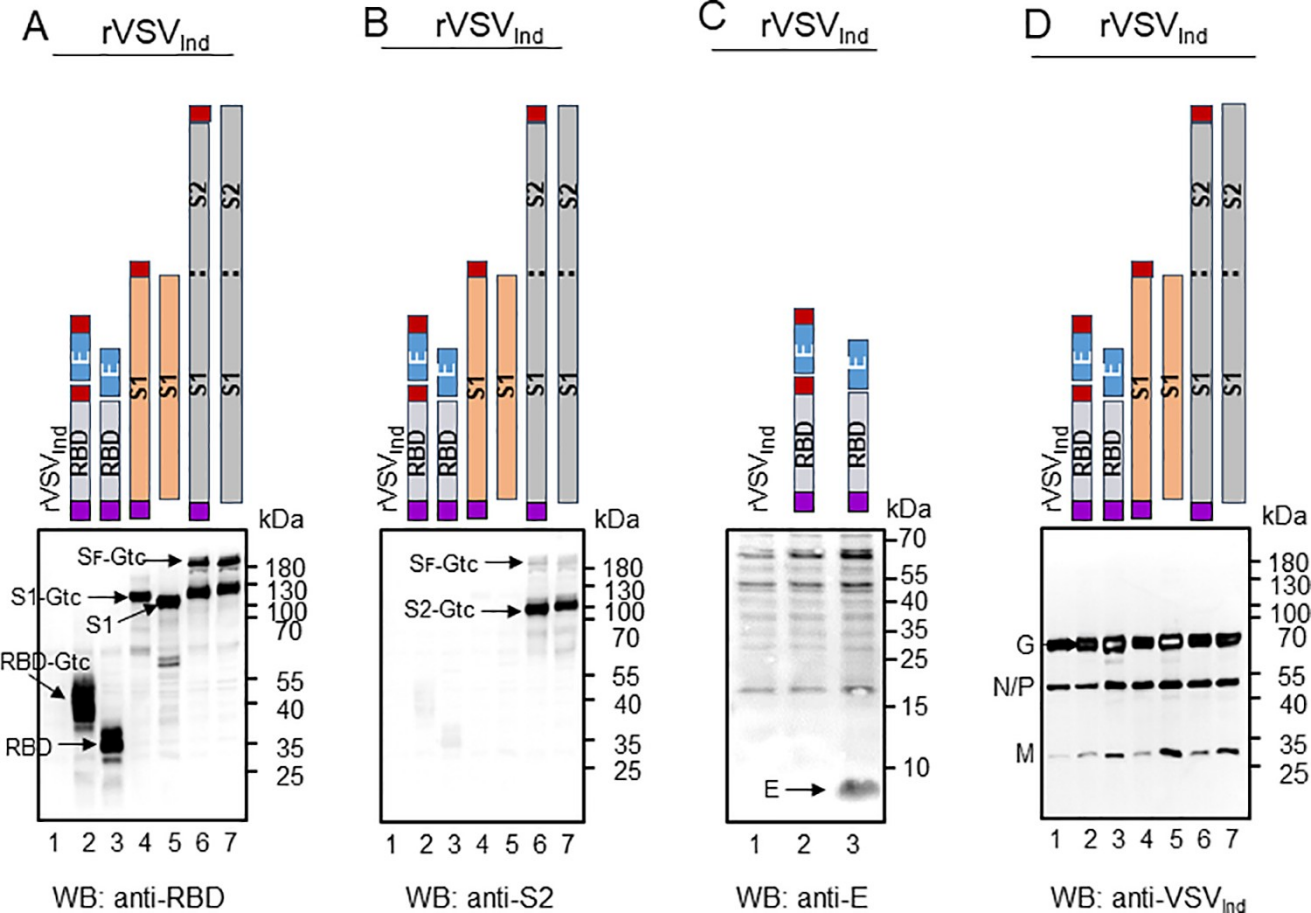
Expression of SARS-CoV-2 proteins from rVSV_Ind_-SARS-CoV-2. To check the expression of SARS-CoV-2 RBD, S1, S_F_, and E proteins from the rVSV_Ind_-SARS-CoV-2 infected cells, BHK-21 cells were infected with the virus at an MOI of 6. After six hours incubation at 37°C, cell lysates were prepared and protein expression was determined by Western blot analysis. Cell lysates were loaded in 5 μg quantity for SDS-PAGE. RBD, S1, and S_F_ proteins were detected by rabbit antibody against SARS-CoV-2 RBD. S2 protein was detected by rabbit antibody against SARS-CoV-2 S2. E protein was detected by rabbit antibody against SARS-CoV-2 E peptides. **(A)** Expression of RBD, S1, and S_F_ with and without msp and Gtc. **(B)** Expression of S2 with and without Gtc. **(C)** Expression of E protein. **(D)** Expression of VSV_Ind_ N, P, M, and G proteins. Purple box: honeybee msp, red box: VSV Gtc.

To construct SARS-CoV-2 vaccines, we individually inserted codon-optimized genes of the receptor-binding domain (RBD), the N-terminal half of the Spike protein (S1), and full-length S (S_F_) coding sequences of SARS-CoV-2 between the VSV glycoprotein (G) and polymerase (L) genes in both the New Jersey serotype and Indiana serotype of rVSV vectors (Fig 1). We designed the rVSV-RBD constructs to also encode the SARS-CoV-2 envelope (E) protein because we previously found that RBD plus E induced better neutralizing antibodies against MERS-CoV than RBD alone. We used Western blotting to compare expression levels of S_F_, S1, S2, RBD and E proteins from the various constructs described in Fig 1 in rVSV_Ind_-infected BHK-21 cells. S1 and S2 proteins were generated after cleavage of S_F_ by the cellular protease furin [25,26]. We detected robust expression of RBD (Fig 2A), S_F_ (Fig 2A) and S1 cleavage product (Fig 2A) using an anti-RBD antibody, and of S_F_ and S2 proteins (Fig 2B) from the VSV-S_F_ and VSV-msp-S_F_-Gtc constructs using the anti-S2 antibody. The anti-S2 antibody detected its binding domain less efficiently in the S_F_ compared to the S2 cleavage product. We noted a moderate level of E protein expression in cells infected with rVSV carrying RBD and E protein genes using the anti-E antibody (VSV-msp*-*RBD+E, Figs 2C and S1C). For unknown reason, we did not detect expression of E protein with the VSV-msp*-*RBD-Gtc*+*E-Gtc construct. As expected, the addition of VSV Gtc to RBD and S1 increased their protein molecular mass by 5.5 kDa (Fig 2A lanes 2–5). In contrast, we did not observe a difference in levels of S protein constituents in the cell lysate with the addition of msp or Gtc. All cell lysates contained a similar amount of VSV proteins (Fig 2D). Expression levels of S_F_, S1, S2, and RBD proteins in rVSV_NJ_ infected cells (S1 Fig) were very similar to levels in rVSV_Ind_ infected cells (Fig 2A, 2B and 2C).

Notably, various SARS-CoV-2 S or S-derived proteins migrated more slowly than their predicted protein size, regardless of the msp or Gtc addition. Our treatment of infected cell lysates with Peptide N-Glycosidase F (PNGase F) prior to Western blot analysis resulted in a loss of these more slowly migrating forms, confirming that the SARS-CoV-2 proteins are highly glycosylated (S2 Fig). Glycosylation of viral proteins has been implicated for specific antibody responses [27–29]. Together, our data show that introduction of SARS-CoV-2 S_F_, S1, and RBD into rVSV_Ind_ and rVSV_NJ_ resulted in efficient expression of glycosylated SARS-CoV-2 proteins in infected cells.

### SARS-CoV-2 protein secretion and incorporation into viral particles is enhanced by the addition of msp and Gtc

**Fig 3:**
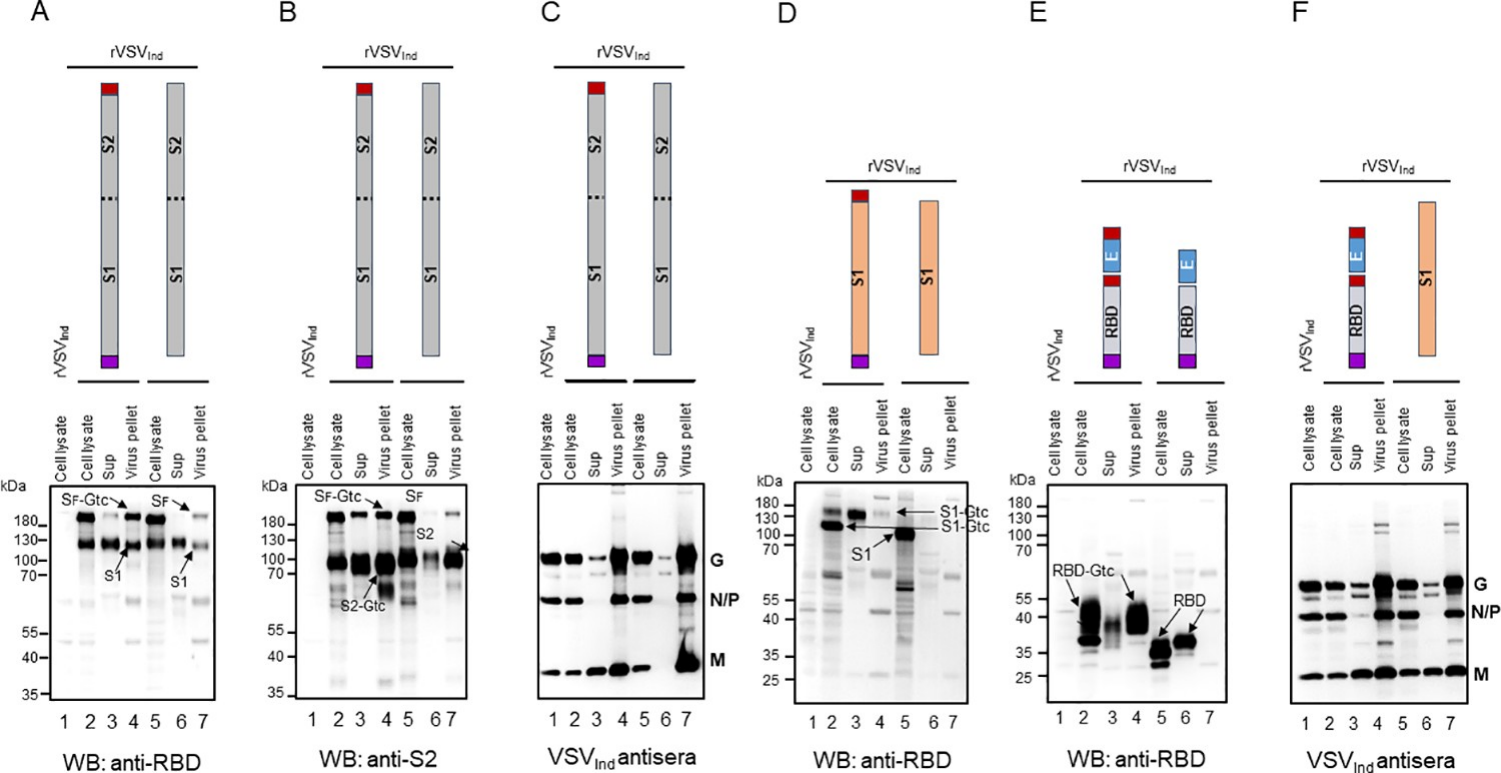
Incorporation of S_F_, S1, S2, and RBD proteins with VSV Gtc into rVSV_Ind_ viral particles. Incorporation of SARS-CoV-2 S_F_, S1, S2, and RBD with or without VSV Gtc into rVSV_Ind_ particles was examined by infecting BHK-21 cells with rVSV_Ind_-SARS-CoV-2 at an MOI of 3. The rVSV_Ind_-SARS-CoV-2 infected cells were incubated at 31°C for 6 hrs. Infected cell lysates were prepared in lysis buffer (lanes 1, 2, and 5). Culture media from the infected cells was centrifuged at 500 x g for 10 minutes and supernatant was filtered through a 0.45 μm filter to remove cell debris. The filtered culture media was loaded onto 1 ml of 25% sucrose cushion and ultra-centrifuged at 150,900 x g for 3 hrs. Supernatant on top of the 25% sucrose cushion was collected to check the soluble proteins in the media (lanes 3 and 6). Pelleted samples were checked for proteins incorporated into VSV particles (lanes 4 and 7). We detected RBD, S1, and S_F_ proteins by Western blot using an antibody against the SARS-CoV-2 RBD protein. S2 and S_F_ proteins were detected by rabbit antibody against SARS-CoV-2 S2. **(A)** Detection of S_F_ and S1 proteins in cell lysate, concentrated culture media, and virus pellet from cells infected with rVSV_Ind_-msp-S_F_-Gtc or rVSV_Ind_-S_F_. **(B)** Detection of S_F_ and S2 proteins in cell lysate, concentrated culture media, and virus pellet from cells infected with rVSV_Ind_-msp-S_F_-Gtc or rVSV_Ind_-S_F_. **(C)** Detection of VSV_Ind_ proteins in cell lysate, concentrated culture media, and virus pellet from cells infected with rVSV_Ind_-msp-S_F_-Gtc or rVSV_Ind_-S_F_. **(D)** Detection of S1 protein in cell lysate, concentrated culture media, and virus pellet from cells infected with rVSV_Ind_-msp-S1-Gtc or rVSV_Ind_-S1. **(E)** Detection of RBD proteins in cell lysate, concentrated culture media, and virus pellet from cells infected with rVSV_Ind_-msp-RBD-Gtc+E-Gtc or rVSV_Ind_-msp-RBD+E. **(F)** Detection of VSV_Ind_ proteins in cell lysate, concentrated culture media, and virus pellet from the cells infected with rVSV_Ind_-msp-RBD-Gtc+E-Gtc or rVSV_Ind_-msp-RBD+E. Purple box: honeybee msp, red box: VSV Gtc.

We then used Western blotting to analyze the incorporation of SARS-CoV-2 proteins into viral particles as pseudotype VSV, and their secretion from infected cells. We analyzed centrifuged virus pellets and the remaining supernatant fractions using antibodies to RBD, S2, or VSV and compared them to cell lysates (Fig 3). In contrast to the similar levels observed in the cell lysates (Fig 2), levels of S protein constituents expressed on viral particles, or within the supernatant (free of virus particles), were much higher for the S_F_ and S1 vaccine constructs with msp and Gtc modifications (compare lane 3 & 4 with 6 & 7 in Fig 3A, 3B, 3D and 3E). We observed similar levels of VSV G and M proteins for each of the rVSV constructs (Fig 3C and 3F). This indicates that differences in S protein secretion and virion incorporation were not due to VSV particle production. Results for rVSV_Ind_ (Fig 3) and rVSV_NJ_ (S3 Fig) S_F_ vaccine constructs were similar.

Analysis of the rVSV-RBD constructs showed that while RBD protein, with and without Gtc modification was readily detected in the cell lysates, only RBD with the Gtc addition was detected in virus pellets (Fig 3E, lane 4). This indicates that VSV Gtc is required for RBD to be incorporated into rVSV particles or released into the supernatant (Fig 3E, lanes 3 and 6). Taken together, these data show that msp and Gtc modified SARS-CoV-2 S_F_, S1, and RBD proteins are efficiently incorporated into rVSV particles and are secreted from infected cells. In contrast, msp and Gtc modified S1 protein was not incorporated into rVSV efficiently (Fig 3D), suggesting that S2 protein is required for efficient incorporation of S1.

### Incorporation of SARS-CoV-2 proteins in highly purified rVSV virions

**Fig 4:**
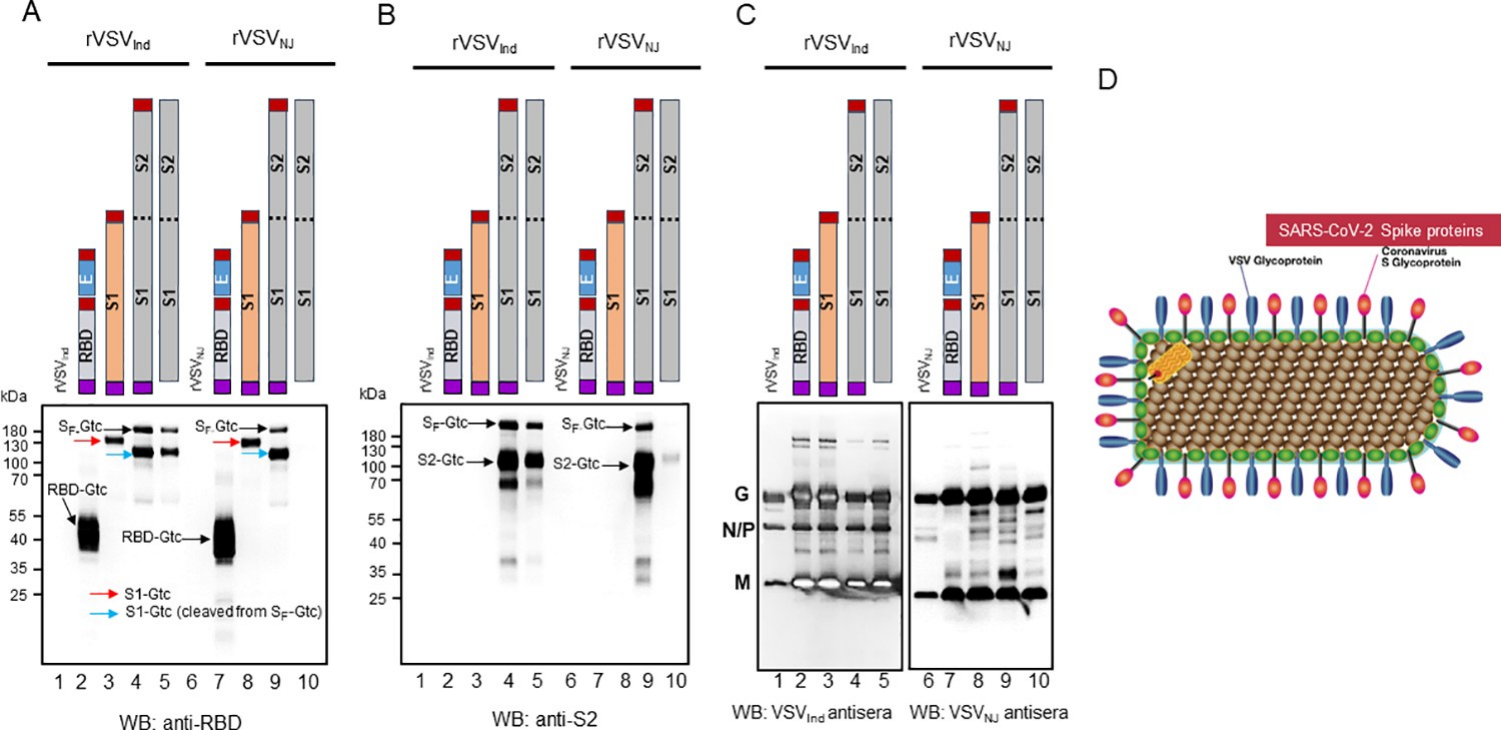
SARS-CoV-2 RBD, S2, and S_F_ with VSV G transmembrane domain and cytoplasmic tail (Gtc) are incorporated efficiently into highly purified rVSV virions. To examine immune responses in mice, it was first necessary to purify rVSV-SARS-CoV-2 viral particles by anion-exchange chromatography. One μg of the purified rVSV-SARS-CoV-2 was analyzed by SDS-PAGE and the presence of RBD, S1, S2, and S_F_ was determined by Western blot analysis. **(A)** Detection of RBD, S1, and S_F_ on VSV particles. **(B)** Detection of S2 and S_F_ on VSV particles. **(C)** Detection of VSV_Ind_ and VSV_NJ_ proteins. **(D)** Depicted model of pseudotype recombinant VSV virions with three different forms of SARS-CoV-2 Spike proteins. rVSV pseudotypes are formed when rVSV-SARS-CoV-2 Spike proteins are expressed with the msp at the NH_2_-terminus and VSV Gtc at the COOH-terminus. Purple box: honeybee msp, red box: VSV Gtc.

To prepare rVSV vaccine vectors for immunization, we purified rVSV containing the SARS-CoV-2 genes, as well as control viruses without SARS-CoV-2 genes, by anion-exchange chromatography. Western blot analysis revealed appreciable quantities of S_F_, RBD, S1, and S2 incorporated into rVSV pseudotype virions when these proteins were expressed with msp and Gtc (Fig 4). In addition, our results showed that rVSV forms pseudotype virions containing both VSV G and SARS-CoV-2 S glycoproteins (Figs 4D and S4). Thus, purified rVSV-SARS-CoV-2-msp-S_F_-Gtc vaccine virus carries spike proteins of SARS-CoV-2. As expected, msp and Gtc modified S_F_ or S1 protein had higher virion incorporation into the rVSV pseudotypes when compared to the non-modified forms. This difference was particularly apparent for rVSV_NJ_ vaccines where the non-modified S_F_ had no detectable incorporation of S_F_ or S1, and barely detectable S2. Similar quantities of VSV G and M proteins were observed between the different rVSV SARS-CoV-2 vaccine constructs. The results demonstrated that purified rVSV-SARS-CoV-2 carry sufficient RBD, S1, S2, and S_F_ proteins to induce immunity. The relative amount of infectious rVSV-SARS-CoV-2-msp-S_F_-Gtc vectors harboring gRNA was between 1 and 2% (S1 Table) as an estimate of total virus particle count. All VSV particles contain VSV gRNA based on process of rhabdovirus assembly. The ratio of infectious to total virus particles for wild type VSV is estimated to be 1 to 5%, which is similar to that observed with our VSV vaccine vectors [30–32]. Recent studies suggest that aggregation of infectious VSV particles can lead to co-infection of a cell resulting in a single plaque, often reducing the ratio of infectious to non-infectious particles [33,34]. It is important to stress that all infectious, defective-interfering, or non-infectious VSV vectors found as single particle or as an aggregate, will express the SARS-CoV-2 spike protein (S4 Fig) and are immunogenic (see below).

### Vaccination of C57BL/6 mice with rVSV-SARS-CoV-2 induced high IgG titers against SARS-CoV-2 spike proteins

**Fig 5:**
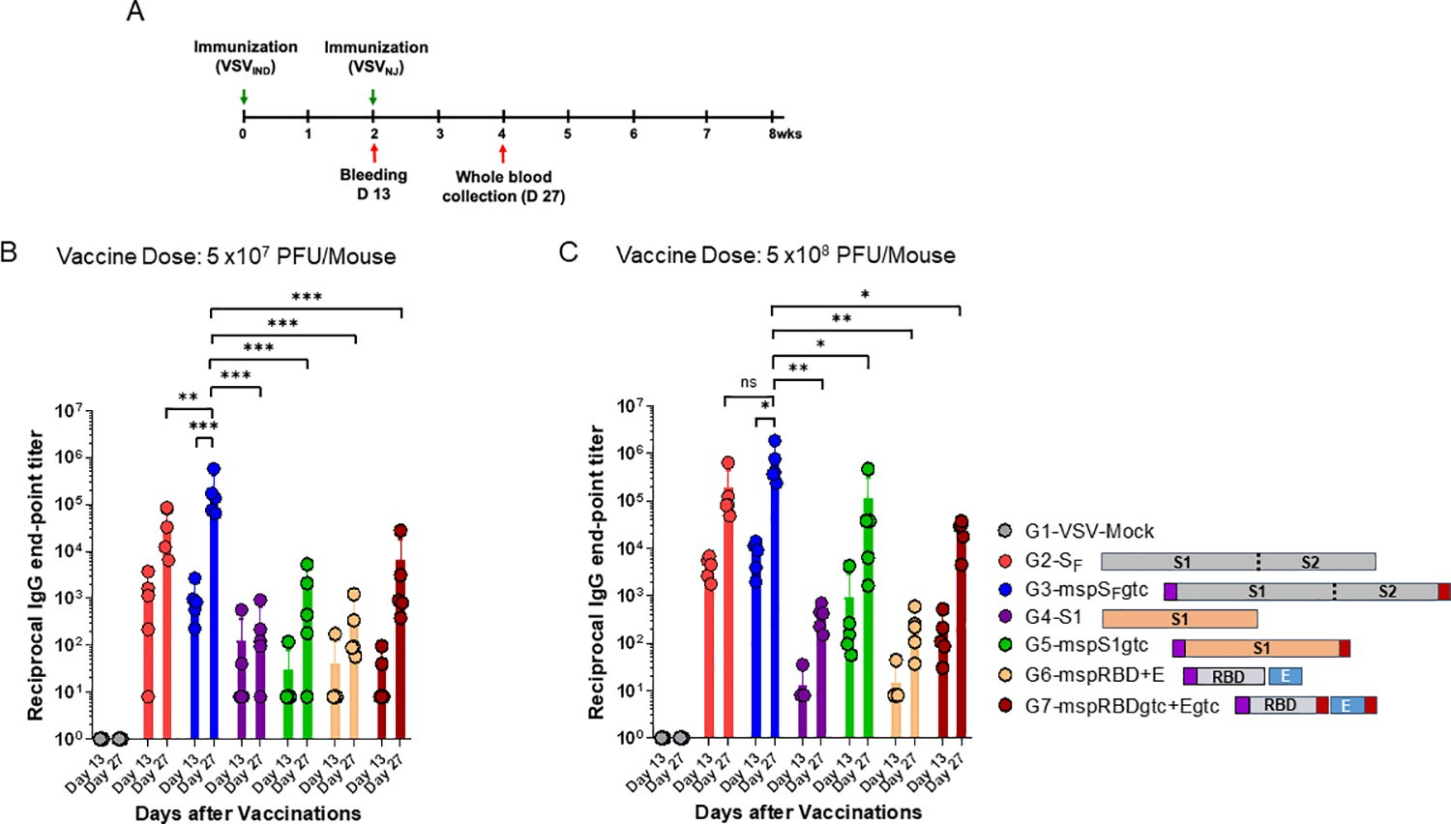
Full-length S_F_ protein with the melittin signal peptide (msp) at the amino terminus and VSV G protein transmembrane domain and cytoplasmic tail (Gtc) at the carboxyl terminus induce the highest IgG titers against the SARS-CoV-2 Spike(ΔTM) protein. Mice were prime immunized with rVSV_Ind_-SARS-CoV-2 and boost immunized with rVSV_NJ_-SARS-CoV-2 two weeks after prime-immunization. Serum was collected to determine SARS-CoV-2 S1 protein-specific antibody levels by ELISA on day 13, one day before boost-immunization, and on day 27, two weeks after boost-immunization. **(A)** Prime-boost vaccination schedule. **(B)** Spike(ΔTM)-specific IgG titer after the prime-boost vaccination with doses of 5X10^7^ PFU/mouse (**C)** Spike(ΔTM)-specific IgG titer after the prime-boost vaccination with doses of 5X10^8^ PFU/mouse. Statistical significance was determined by two-way ANOVA with Tukey’s correction (*, p < 0.05; **, p < 0.005; ***, p< 0.001; ns, not significant). The data were presented as means with error bars of standard deviation (n = 5 mice per group). Purple box: honeybee msp, red box: VSV Gtc. VSV-Mock denotes VSV vector alone without any gene insert.

To analyze humoral immune responses towards SARS-CoV-2 S_F_, S1 and RBD, we vaccinated C57BL/6 mice (N = 5/vaccination group) intramuscularly with rVSV vaccine vectors at 5x10^7^ PFU/mouse or 5x10^8^ PFU/mouse (S2 Table). We prime vaccinated each mouse with rVSV_Ind_ constructs (Fig 5A). Two weeks after prime immunization, we boost-immunized the mice with rVSV_NJ_ constructs. We then collected sera on days 13 (one day before boost immunization) and day 27 (two weeks after boost-immunization) (Fig 5A). We detected higher levels of Spike(ΔTM)-specific IgG antibodies by ELISA in sera of mice immunized with the 5x10^8^ PFU dose than with the 5x10^7^ PFU dose, regardless of rVSV construct (Fig 5B and 5C). For both doses, Spike(ΔTM)-specific antibody levels were further increased after boost-immunization (Fig 5B and 5C). Overall, mice immunized with rVSV-msp-S_F_-Gtc or rVSV-S_F_ produced the highest level of Spike(ΔTM)-specific antibodies.

### Modified SARS-CoV-2 S_F_ protein induced potent neutralizing antibodies

To assess the possibility that this VSV-based vaccine prevents SARS-CoV-2 infection, we measured the level of SARS-CoV-2 neutralizing antibodies in immunized mice sera on day 13 and/or day 27 sera using a FRNT_50_ assay. In all immunized groups, neutralizing antibody titers were significantly increased after boost (Table 1 and S5 Fig). Mice immunized with rVSV-msp-S_F_-Gtc or rVSV-S_F_ had the highest average neutralizing antibody titers (1/13,824 and 1/4,864 respectively) after boost compared to all other groups of immunized mice, with neutralizing titers up to a high of 1/40,960. These results indicate that the S_F_ protein induced stronger neutralizing antibody responses than S1 or RBD+E immunogens, and modification of S_F_ with msp and Gtc further enhanced neutralizing antibody titers. It is not clear why the full-length spike protein induced the highest level of neutralizing antibodies when compared with RBD or S1. The induction of a strong neutralizing antibody may depend on the efficiency of full-length spike protein incorporation into the pseudotype VSV. Moreover, all mice immunized with 5x10^8^ PFU induced stronger neutralizing antibody responses compared to mice immunized with 5x10^7^ PFU (Table 1). Taken together, a vaccine regimen in mice involving prime immunization with 5x10^8^ PFU of rVSV_Ind_-msp-S_F_-Gtc, followed two weeks later by boost immunization with 5x10^8^ PFU rVSV_NJ_-msp-S_F_-Gtc induced highly potent neutralizing antibody responses.

**Table 1:** Titration of Neutralizing Antibodies against SARS-CoV-2 in Mice after Prime-Boost Vaccination with rVSV-SARS-CoV-2.

50% Focus Reduction Neutralization antibody titer (FRNT_50_) against SARS-CoV-2											
	rVSV (5X10^8^ PFU)	rVSV-S_F_ (5X10^7^ PFU)		rVSV-S_F_ (5X10^8^ PFU)		rVSV-msp-S_F_-Gtc (5X10^7^ PFU)		rVSV-msp-S_F_-Gtc (5X10^8^ PFU)		rVSV-msp-S1-Gtc (5X10^8^ PFU)	rVSV-msp-RBD-Gtc+E-Gtc (5X10^8^ PFU)
	D 27 (Pooled)	D 13 (Pooled)	D 27	D 13	D 27	D 13	D 27	D 13	D 27	D 27	D27
**1**	20	40	640	40	1,280	40	10,240	40	10,240	320	40
	20	40	640	40	1,280	40	10,240	40	10,240	320	40
**2**	40	40	1,280	80	1,280	40	10,240	80	2,560	160	160
	20	40	2,560	80	1,280	40	5,120	80	2,560	160	160
**3**		40	80	40	10,240	40	2,560	160	10,240	20	80
		40	80	40	10,240	20	2,560	160	10,240	20	80
**4**			640		2,560		640		5,120		
			640		5,120		640		5,120		
**5**			160		10,240		640		40,960		
			160		5,120		640		40,960		
**Average**	**25**	**40**	**688**	**53**	**4,864**	**37**	**4,352**	**93**	**13,824**	**167**	**93**
**STDEV**	10	0	757.3	20.7	3,993.4	8.2	4,296.4	54.7	14,641.6	134.3	54.7

We then investigated whether our rVSV vaccine virus is neutralized by the sera of individuals with pre-existing anti-SARS-CoV-2 antibodies. We incubated the rVSV-SARS-CoV-2-msp-S_F_-Gtc pseudovirions with sera from five COVID-19 patients or with anti-VSV antibodies and infected human 293T cells with and without ACE2 expression. We found that pseudotype VSVs incubated with sera from recovered COVID-19 patients can infect both 293T and 293T-ACE2 cells (S6 Fig). These results indicate that rVSV is resistant to neutralization by 1/40 dilution of patients’ sera.

**Fig 6:**
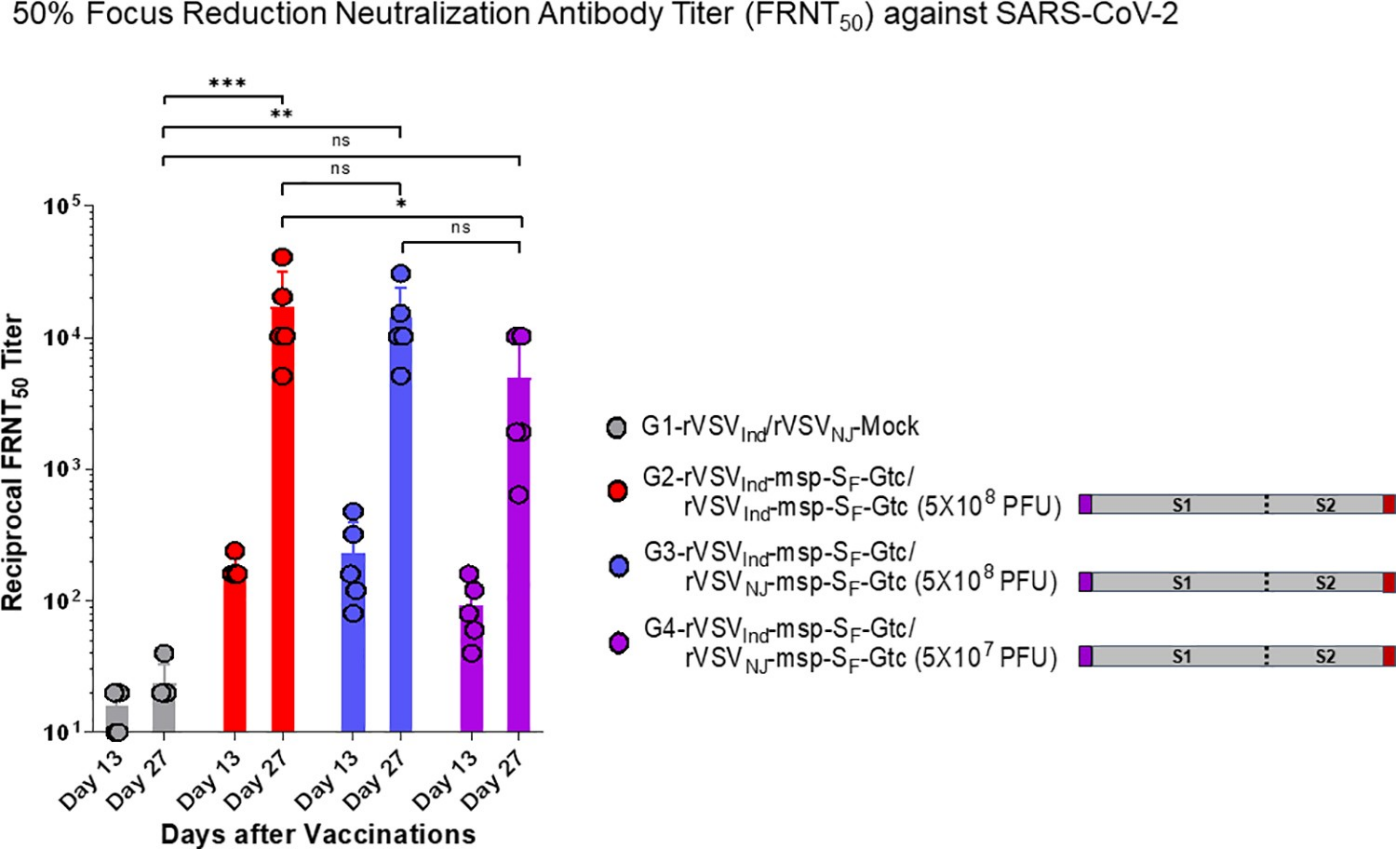
Prime-boost vaccination of hACE2 transgenic mice induces high levels of neutralizing antibodies against SARS-CoV-2. Six-week-old female hACE2 transgenic mice were prime vaccinated with rVSV_Ind_-msp-S_F_-Gtc and boost immunized with rVSV_Ind_-msp-S_F_-Gtc or rVSV_NJ_-msp-S_F_-Gtc two weeks after the prime-vaccination. Serum was collected on day 13, one day before the boost-vaccination and on day 27, two weeks after the boost-vaccination. SARS-CoV-2 neutralization was determined by FRNT_50_ assay. Statistical significance was determined by two-way ANOVA with Tukey’s correction (*, p < 0.05; **, p<0.005; ***, p< 0.001; ns, not significant). The data were presented as means with error bars of standard deviation (n = 5 mice per group). Purple box: honeybee msp, red box: VSV Gtc. VSV-Mock denotes VSV vector alone without any gene insert.

**Fig 7:**
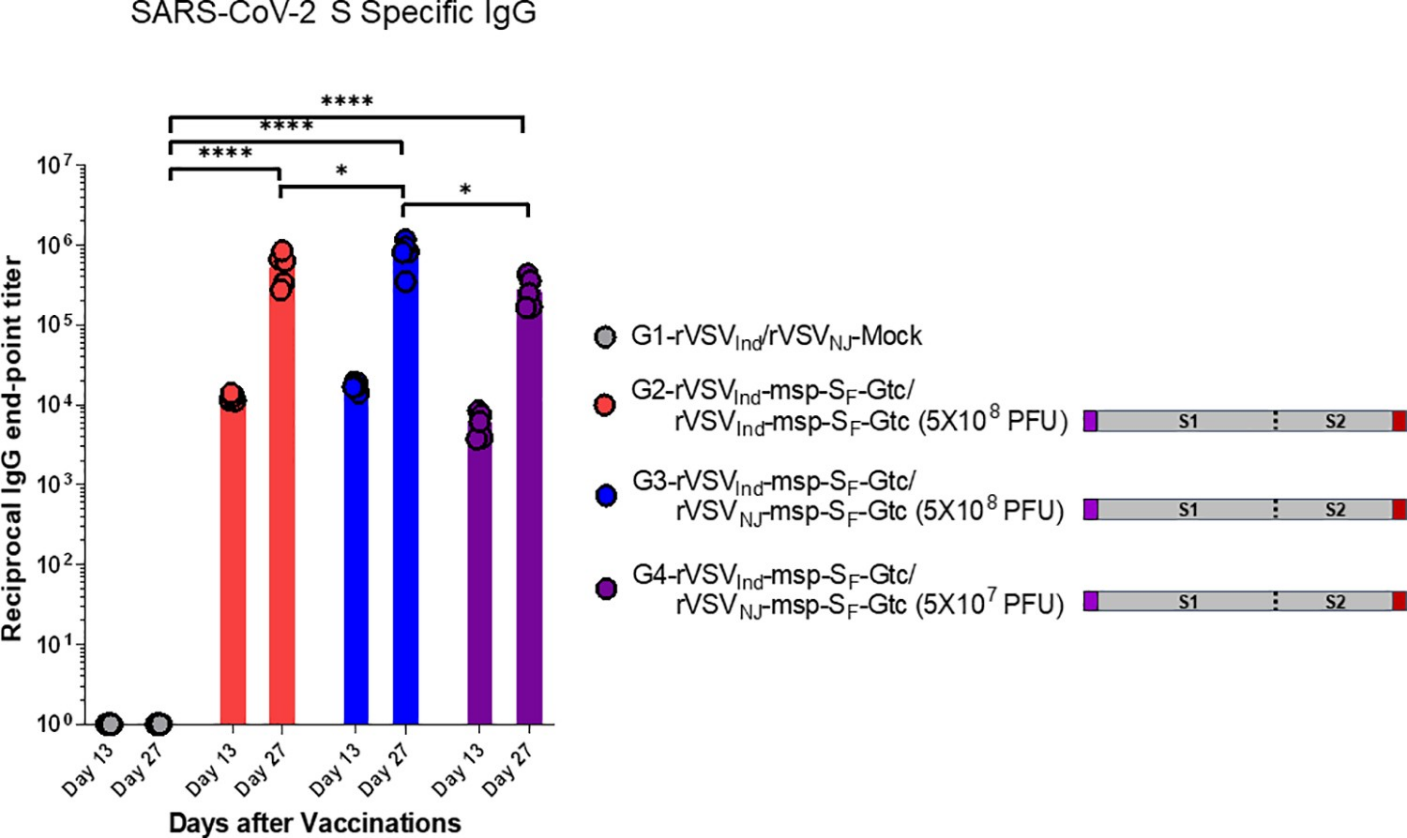
Prime-boost vaccination of hACE2 transgenic mice with rVSV-msp-S_F_-Gtc induces a high level of Spike(ΔTM) specific IgG. Six-week-old female hACE2 transgenic mice were prime vaccinated with rVSV_Ind_-msp-S_F_-Gtc and boost vaccinated with rVSV_Ind_-msp-S_F_-Gtc or rVSV_NJ_-msp-S_F_-Gtc two weeks after the prime-vaccination. Serum was collected to determine the SARS-CoV-2 Spike(ΔTM) protein-specific antibody level by ELISA on day 13, one day before the boost-vaccination and on day 27, two weeks after the boost-vaccination. Statistical significance was determined by two-way ANOVA with Tukey’s correction (*, p < 0.05; **, p<0.005, ***, p<0.001, ****, p< 0.0001; ns, not significant). The data were presented as means with error bars of standard deviation (n = 5 mice per group). Purple box: honeybee msp, red box: VSV Gtc. VSV-Mock denotes VSV vector alone without any gene insert.

Anti-vector mediated immunity is expected considering both the VSV-G and SARS-CoV-2-S are co-expressed on the VSV vector surface. As compared to other viral vectors for vaccinations, non-pathogenic, replicating VSV vector can achieve stronger immune responses with considerably less vaccine vector load upon immunization. Replication of these vaccines are also self-limiting because the anti-VSV G antibody response will eventually control vector replication. In S6B Fig, we show that the sera of SARS-CoV-2 infected patients and an anti-S SARS-CoV-2 neutralizing antibody did not block the replication of the Indiana serotype of rVSV-SARS-CoV-2-msp-S_F_-Gtc. This indicates that, despite high levels of the S_F_ on the vector surface, only VSV-G and not SARS-CoV-2 S_F_ mediated vector replication. In S3B Table, we show that sera obtained post rVSV_Ind_-SARS-CoV-2-msp-S_F_-Gtc vaccination of mice could neutralize the replication of VSV_Ind_ vector but only at a low sera dilution (1:50) suggesting some level of control of vector replication following vaccination. The sera from mice primed with rVSV_Ind_-SARS-CoV-2-msp-S_F_-Gtc had higher neutralizing antibody titers to SARS-CoV-2 (a 50% neutralization titer of ~1/200; Fig 6) but that the anti-S NAb in this serum was likely not involved in VSV_Ind_ inhibition (as suggested by S6B Fig). Sera from mice primed with rVSV_Ind_-SARS-CoV-2-msp-S_F_-Gtc could not neutralize the New Jersey serotype vaccine, rVSV_NJ_-SARS-CoV-2-msp-S_F_-Gtc (S3A Table). Given that the neutralizing SARS-CoV-2 titers of sera increased over 100-fold upon the boost with either the New Jersey or Indiana serotype of rVSV-SARS-CoV-2-msp-S_F_-Gtc (Fig 6), the boosting vector is clearly inducing enhanced humoral immunity (Figs 6 and 7) and protection from SARS-CoV-2 challenges (see below). If there was immediate antibody-mediated vaccine vector reduction based on anti-G_Ind_ antibodies induced by the prime, we would have anticipated much higher anti-S IgG levels and sera neutralization with the New Jersey serotype vector boost than with the Indiana serotype vector boost, because the latter would be controlled by the vector mediated immunity from the prime. Instead the anti-S IgG levels (Fig 7) and sera neutralization (Fig 6) was the same for the homologous VSV_Ind_-VSV_Ind_ and heterologous VSV_Ind_-VSV_NJ_ prime-boost.

### S_F_ protein with msp and Gtc modifications induced high numbers of IFN-γ secreting cells

**Fig 8:**
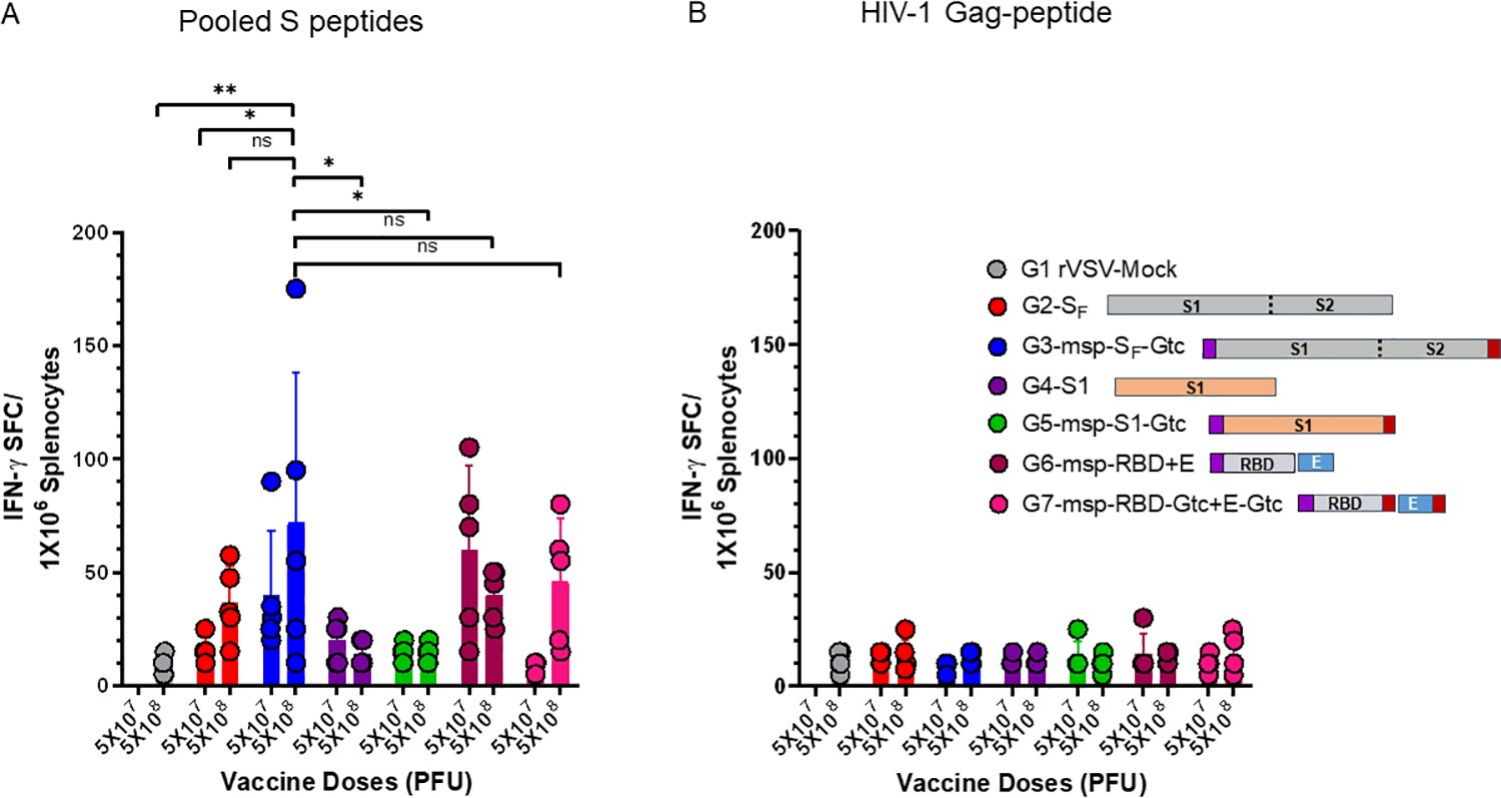
T cell responses against SARS-CoV-2 Spike protein. Mice were primed with rVSV_Ind_-SARS-CoV-2 followed with rVSV_NJ_-SARS-CoV-2 two weeks after prime-immunization. Two weeks after the boost-immunization, splenocytes were prepared and stimulated with a PepTivator SARS-CoV-2 Prot_S [**(A)** (S5 Fig)], or an irrelevant (control) peptide derived from the HIV Gag **(B)**. IFN-γ spot-forming units (SFUs) were enumerated by ELISPOT. Statistical significance was determined by two-way ANOVA with Tukey’s correction (*, p < 0.05; **, p < 0.005; ns, not significant). Data are presented as mean SFU numbers with error bars representing standard deviation (n = 5 mice per group). Purple box: honeybee msp, red box: VSV Gtc. VSV-Mock denotes VSV vector alone without any gene insert.

To investigate the cellular immune responses elicited by our rVSV-SARS-CoV-2 vaccines, we tested T cell immune responses against SARS-CoV-2 Spike protein-derived peptides in an IFN-γ ELISpot assay using a peptide pool (with an 11 amino acid overlap) covering the immunodominant sequence domains of the Spike glycoprotein of SARS-CoV-2 (GenBank MN908947.3, Protein QHD43416.1) (S7 Fig). We analyzed splenocytes from mice immunized with the six different SARS-CoV-2 vaccine constructs. Results indicated the presence of responsive IFN-γ-secreting cells from mice in several immunized groups, with rVSV-msp-S_F_-Gtc immunized mice having the highest number of IFN-γ-secreting cells (Fig 8A). As a negative control, HIV-1 Gag peptides showed minimal IFN-γ ELISpot responses for all groups (Fig 8B). Overall, the pattern of T cell immune responses elicited by the six different rVSV-SARS-CoV-2 vaccines are consistent with humoral immune responses shown in Fig 5 and Table 1.

### rVSV-msp-S_F_-Gtc-vaccinated hACE2 transgenic mice were protected from wild-type SARS-CoV-2 challenge

**Fig 9:**
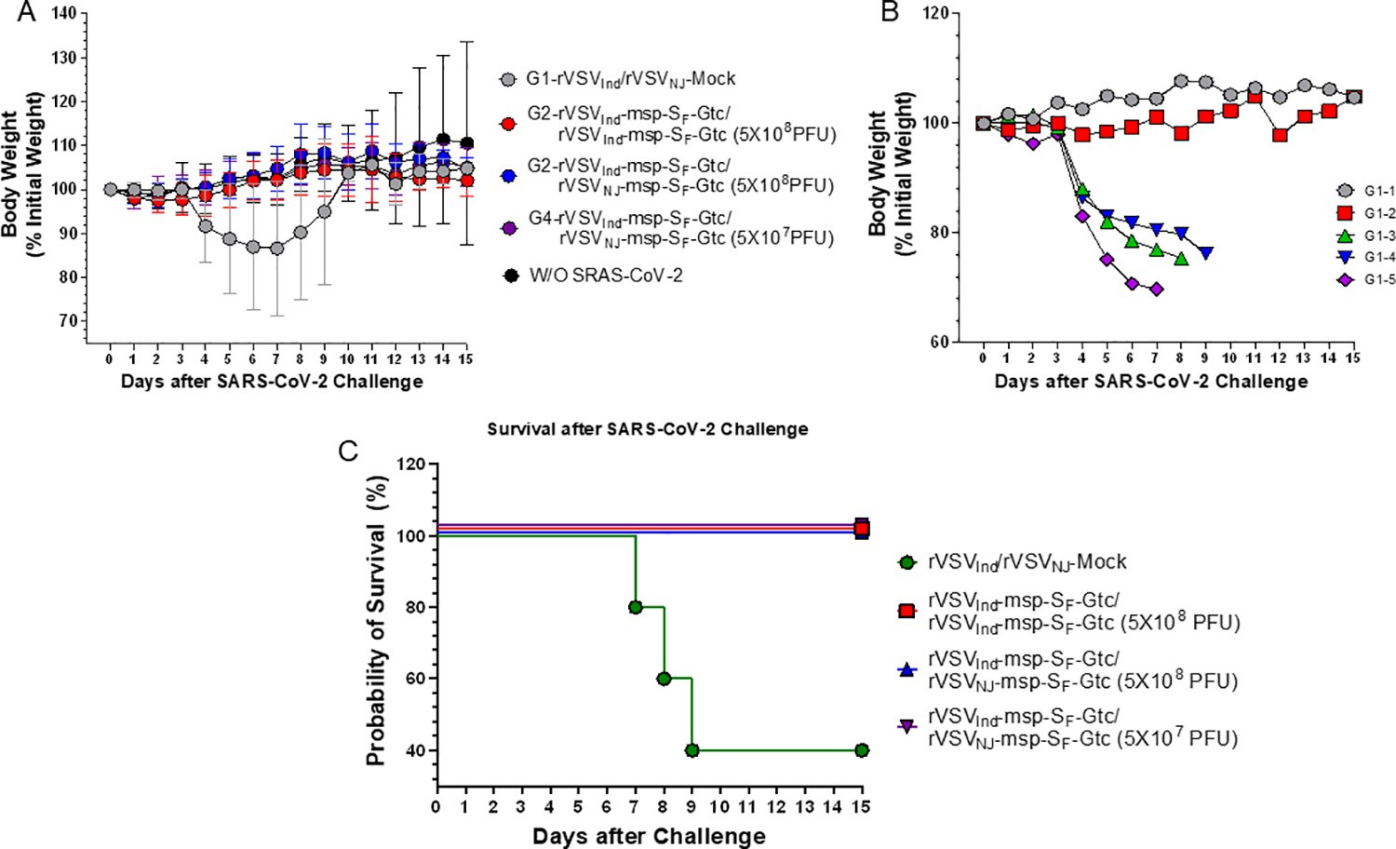
Bodyweight and survival of the vaccinated and SARS-CoV-2 challenged hACE2 transgenic mice. Six-week-old female hACE2 transgenic mice (n = 5 per group) were prime-vaccinated with rVSV_Ind_-msp-S_F_-Gtc and boost vaccinated with rVSV_Ind_-msp-S_F_-Gtc or rVSV_NJ_-msp-S_F_-Gtc two weeks after prime-vaccination. Four weeks after boost-vaccination (S6 Table), mice were challenged intranasally with 1x10^5^ PFU of SARS-CoV-2. The survival and body weight of each mouse was monitored daily. **(A)** Average bodyweights of mice in each vaccinated group. **(B)** Individual body weights for mice vaccinated with rVSV-Mock and challenged with SARS-CoV-2. **(C)** Mouse survival after SARS-CoV-2 challenge. VSV-Mock denotes VSV vector alone without any gene insert.

**Fig 10:**
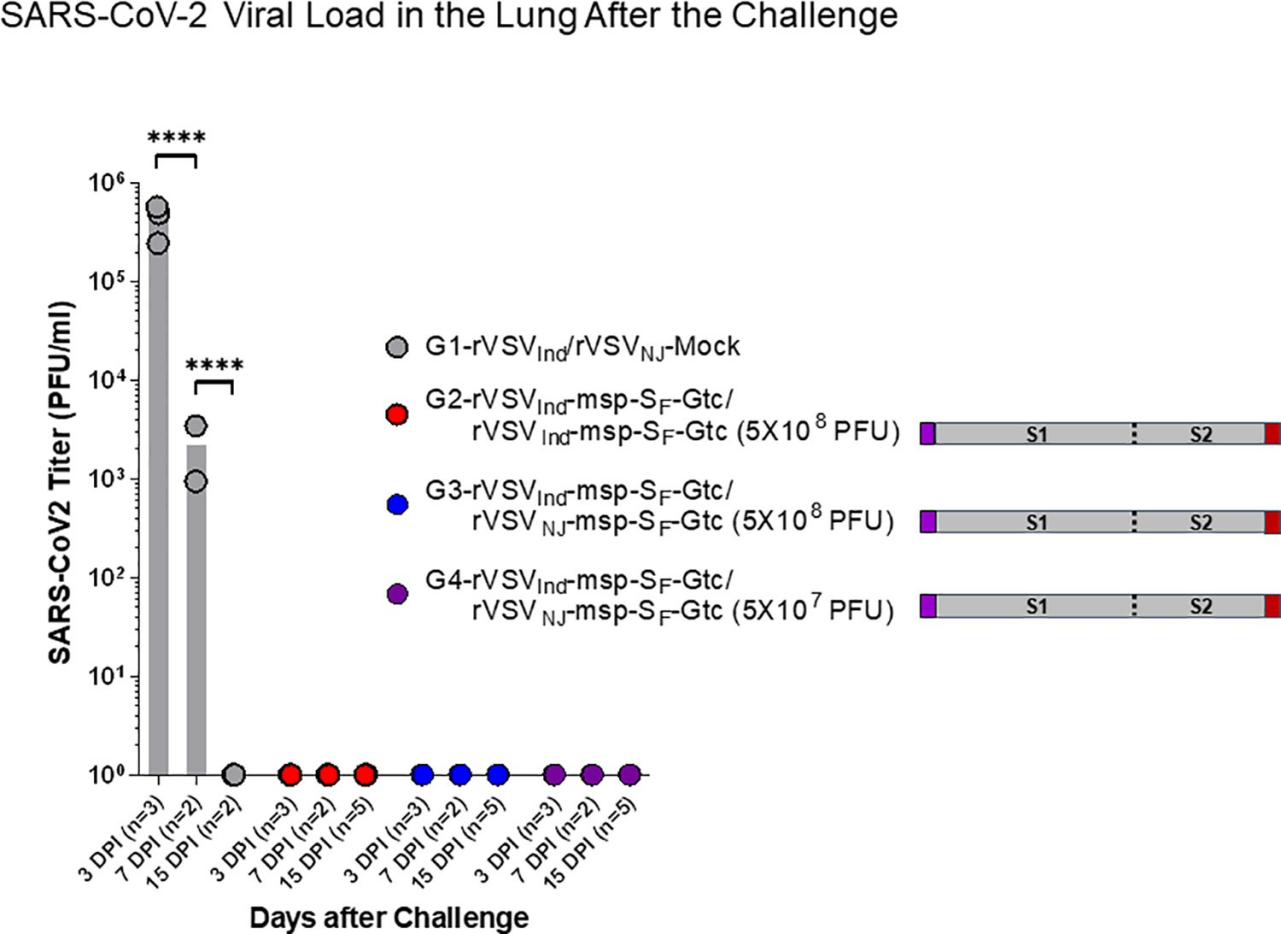
SARS-CoV-2 viral loads in the lungs of vaccinated and challenged hACE2 transgenic mice. Human ACE2 transgenic mice were vaccinated and challenged with SARS-CoV-2 as described in Fig 9. Right lobes of mice lungs were aseptically removed from the mice on day 3, day 7, and day 15 after SARS-CoV-2 challenge. Infectious SARS-CoV-2 was quantified by plaque assay on Vero E6 cells. Statistical significance was determined by two-way ANOVA with Tukey’s correction (****, p< 0.0001). VSV-Mock denotes VSV vector alone without any gene insert.

Since S_F_ with msp and Gtc modifications yielded the highest level of neutralizing antibodies in C57BL/6 mice, we selected it for use in a SARS-CoV-2 challenge study. We prime vaccinated hACE2 transgenic mice with Indiana serotype of rVSV_Ind_-msp-S_F_-Gtc (5x10^8^ PFU). Two weeks later, we boost vaccinated with either 5x10^8^ PFU rVSV of the Indiana serotype (group 2) or New Jersey serotype (group 3) harboring the same S protein insert (rVSV_Ind_-msp-S_F_-Gtc or rVSV_NJ_-msp-S_F_-Gtc) (S6 Table). Group 4 was the rVSV_Ind_ prime and rVSV_NJ_ boost with the same S insert, using 5x10^7^ PFU. All vaccine constructs induced Spike(ΔTM)-specific IgG antibodies (as measured by ELISA) with 100-fold higher levels following boost vaccination (Fig 7, day 27). The 5x10^8^ PFU dose yielded slightly elevated antibody levels as compared to the 5x10^7^ PFU dose (Fig 7). The binding and neutralizing antibody findings were directly correlative. The neutralizing antibody levels also increased by over 100-fold after prime and boost vaccinations and were slightly increased at the higher vaccine dose (Fig 6). Four weeks after boost-vaccination, we challenged the mice intranasally with 1x10^5^ PFU of SARS-CoV-2. We monitored the survival and body weight of each mouse daily (Fig 9). All vaccinated hACE2 transgenic mice survived (Fig 9C) and no significant weight loss was observed (Fig 9A). Non-vaccinated control mice lost weight and three out of five animals died within nine days after the challenge (Fig 9B and 9C). Non-vaccinated control mice showed detectable SARS-CoV-2 in the lungs via plaque assay seven days after challenge. In contrast, no SARS-CoV-2 was detectable in the lungs of vaccinated mice (Fig 10). These results showed that the rVSV-SARS-CoV-2-msp-S_F_-Gtc vaccine can prevent detectable SARS-CoV-2 infection in the lungs of hACE2 transgenic mice. Moreover, a prime-boost vaccination regimen with the same or different rVSV serotype generates comparable levels of neutralizing antibodies. Next, we repeated the challenge studies with a 5-fold higher amount of SARS-CoV-2 to determine if vaccinated hACE2 transgenic mice can be protected from a lethal dose of the challenge virus. We found that the rVSV-SARS-CoV-2-msp-S_F_-Gtc vaccine protects animals from a lethal dose of SARS-CoV-2 with a minimum amount of weight loss and lung damage (S8 and S9 Figs). In contrast, the rVSV_Ind_ vector alone immunized mice died with severe lung damage. These results demonstrate that the rVSV-SARS-CoV-2-msp-S_F_-Gtc vaccine protects transgenic mice from lethal infection.

### Histopathology of the lungs after the SARS-CoV-2 challenge

**Fig 11:**
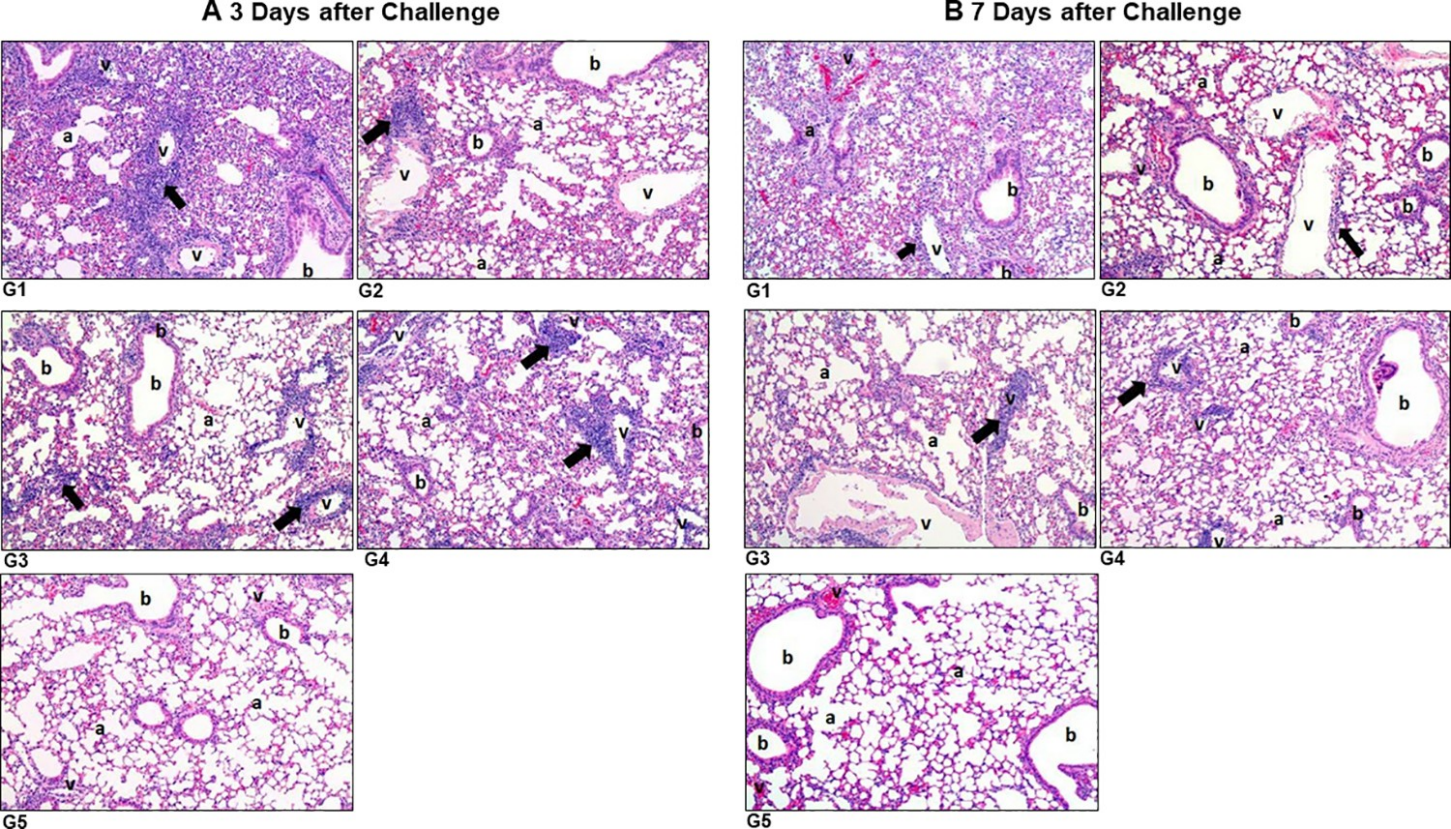
Histopathological findings in the lungs of hACE2 mice. Human ACE2 transgenic mice were vaccinated and challenged with SARS-CoV-2 as described in Fig 9. Left lobes of mice lungs were fixed in 10% buffered formalin on day 3 and day 7 after the SARS-CoV-2 challenge. Lung tissues were processed and embedded in low-melting paraffin, sectioned to a thickness of 3 μm, and stained with hematoxylin and eosin. Stained tissues were examined under a light microscope (Olympus CS41, Japan) with 100X magnification. Note: a, alveolus; b, bronchiole; v, blood vessels. **(A)** Lung tissue 3 days after the SARS-CoV-2 challenge. **(B)** Lung tissue 7 days after SARS-CoV-2 challenge. Arrows show infiltration of inflammatory cells (lymphocytes and macrophages). G1: empty vector infected mice, G2: 5X10^8^ of rVSV_Ind_-msp-S_F_-Gtc/ rVSV_NJ_-msp-S_F_-Gtc vaccinated mice, G3: 5X10^8^ of rVSV_Ind_-msp-S_F_-Gtc/ rVSV_Ind_-msp-S_F_-Gtc vaccinated mice, G4: 5X10^7^ of rVSV_Ind_-msp-S_F_-Gtc/ rVSV_NJ_-msp-S_F_-Gtc vaccinated mice, G5: uninfected mice.

We then performed histopathological examination of lungs from control and vaccinated mice groups at 3, 7, and 15 days post-infection (dpi). We observed no abnormal findings in the uninfected control vehicle mice at all time points (Fig 11 and S7 and S8 Tables). We screened for inflammatory foci characterized by infiltration of inflammatory cells around blood vessels and interstitial alveolar inflammation in the lungs, which are indicators of active SARS-CoV-2 infection, in the challenged hACE2 mice [35]. In the case of inflammatory foci, we graded inflammation based on the criteria described in the materials and methods. The pathologist was blinded to mice in specific vaccination groups. Most blood vessels in the lung sections of empty vector infected mice (group 1) had prominent and heavy perivascular infiltration of inflammatory cells, which was considered to be related to the virus infection. In comparison, vaccinated mice (groups 2–4) showed very weak cell infiltration, close to normal, but there was recognizable inflammatory cell infiltration around some vessels three days after challenge (Fig 11 and S7 and S8 Tables). No specific abnormal findings were observed in the lungs of the control vehicle (uninfected) mice (group 5).

### Neutralization of SARS-CoV-2 variants-of-concern with sera of macaques immunized with rVSV_Ind_-SARS-CoV-2-msp-S_F_-Gtc

With preliminary findings (Figs 8 and S5) of high neutralizing antibody titers in mice immunized in particular with rVSV_Ind_-SARS-CoV-2-msp-S_F_-Gtc, we wanted to confirm these observations in other animal models including rabbits and macaques. Rabbit studies are ongoing. Macaques were immediately available from a terminated HIV vaccine study and three animals were IM immunized with 10^9^ PFU rVSV_Ind_-SARS-CoV-2-msp-S_F_-Gtc as a prime vaccine and boost vaccinated with the same immunogen and dose at 20 days. Details of the protocols are provided in the Supplementary data. Sera utilized in these neutralization assays were collected prior to prime immunization and at 40 days following boost immunization. These three immunized macaques provided sufficient sera, unlike the limited sera availability from mice, for an extensive series of neutralizing titer analyses on sera with multiple VOCs and for subsequent isolation and analyses of neutralizing antibodies. Macaques were not challenged with SARS-CoV-2 due to limited animals and no control group.

Sera from the three macaques were all effective at neutralizing wild-type SARS-CoV-2 expressing Nano-Luciferase (nLuc activity used for quantitation) and wild-type SARS-CoV-2 in which production was measured by qRT-PCR. The neutralizing titer for 50% inhibition of the wild-type nLuc was slightly higher with sera A (1/320) and less potent than that of sera B and C at ~1/640 based on duplicate analyses (S4 Table). Sera B and C were then tested for neutralization of wild-type Wuhan SARS-CoV-2 as well as Alpha, Beta, and Gamma VOCs. Based on the qRT-PCR analyses of quadruplicate diluted sera assays, the wild-type SARS-CoV-2 was inhibited with slightly less sera B (1/640 to 1/1280) than observed with the wild-type nLuc. The Alpha/UK/B.1.1.7 SARS-CoV-2 showed similar sensitivity as the wild-type SARS-CoV-2 to inhibition by sera B and C of macaques prime-boost immunized with rVSV_Ind_-SARS-CoV-2-msp-S_F_-Gtc.

There was slight reduction of inhibition of the Beta/South Africa/B.1.351 and of the Gamma/Brazil/P.1 SARS-CoV-2 by sera B and C when compared to inhibition of wild-type. In S10 Fig, we show comparable levels of SARS-CoV-2 neutralization by the monoclonal Sino-R001 anti-S NAb (panels A) with neutralization by sera of the immunized macaque B (panel E). However, whereas the monoclonal antibody had minimal cross neutralization of the variants alpha, beta and gamma (S10F, S10G, and S10H Fig), it is likely that multiple and/or broadly neutralizing antibodies were present in the sera of immunized macaques (panels F, G, and H). The grey box symbols in panels E to H show the minimal neutralization of the wild-type and VOCs by pre-immunization sera of macaque B.

These analyses suggest a very high level of cross neutralizing antibodies to Alpha, Beta and Gamma VOCs in sera of macaques immunized with SARS-CoV-2-msp-S_F_-Gtc. As discussed below, these rVSV constructs, particularly rVSV_Ind_-SARS-CoV-2-msp-S_F_-Gtc generated higher neutralization activity from sera of both vaccinated mice and macaques than observed with the adenovirus vaccine.

### Cytokine/chemokine response in human PBMCs exposed to and in macaques vaccinated with rVSV_Ind_-SARS-CoV-2-msp-S_F_-Gtc

Using a multiplex Luminex kit and Magpix, we measured the release of 24 different cytokine/chemokine in human PBMCs exposed to rVSV_Ind_-SARS-CoV-2-msp-S_F_-Gtc (S5A Table). At 37°C and the more permissive temperature of 31°C, there was minimal release of both Th1 and Th2-type cytokines/chemokines from PBMCs exposed to a low MOI of 0.01 despite replication of the rVSV_Ind_-SARS-CoV-2-msp-S_F_-Gtc vector and in comparison to the mitogen PMA/Ionomycin treatment (S5A Table). Only at the high MOI of 0.1 and the more permissive 31°C did we observe a low level release of IFN-γ, IL-12, IL-5, IL-18, and IL-28A, or mostly low levels of Th1 cytokines as expected. In the sera of macaques pre- and post-prime-boost immunization, we observed no elevations, significant differences or even trends for differences in the panel of 26 cytokines/chemokines (S5B Table). Based on the high level antibody responses and neutralization activity described in Table 1 and S5 Fig, we suspect an immune activation in the primary and secondary lymphoid tissues during prime-boost vaccinations but this did not lead to systemic inflammation.

## Discussion

This study was designed to: a) develop an effective SARS-CoV-2 vaccine for sustainable and prolonged delivery of antigenic forms of the S protein, and b) to induce effective adaptive immunity for protection against a SARS-CoV-2 challenge. The use of live attenuated rVSV as a vaccine vector has several advantages. Replication-competent forms of rVSV have demonstrated safety and immunogenicity in multiple clinical trials [36,37]. Vaccination with live rVSV stimulates both humoral and cellular immunity to generate long-lasting immunity [38–42]. Human VSV infections are extremely rare, therefore pre-existing immunity to the rVSV vector will be low. In addition, rVSV can be grown to high titers in cell culture, allowing for rapid and efficient biomanufacturing of large vaccine stocks. The VSV vector is nonpathogenic in humans and has been previously employed in a vaccine that delivered the Ebola Glycoprotein (GP) as an immunogen to induce protective responses (reviewed in [43]). VSV lacking its own G glycoprotein is able to replicate in vaccinated humans due to the expression and pseudotyping of the virus particles with Ebola GP. However, unlike the Ebola GP, which utilizes a variety of host cell attachment factors (i.e., C-type lectins, T-cell immunoglobulin and mucin domain 1, and Tyrosine kinase receptor Axl) and macropinocytosis for entry (reviewed in [44]), SARS-CoV-2 primarily uses the cell surface ACE2 protein that is expressed differentially in many different cells of the body [45–49]. The recombinant VSV-ΔG-spike vaccine developed by Yahalom-Ronen [19] showed protection against SARS-CoV-2 challenge. This vaccine uses a G protein gene deleted single serotype VSV vector that resembles Merck’s Ebola virus vaccine. The ΔG VSV vector-based vaccine produces a low yield of virus which poses a major challenge for large quantity vaccine production. Our VSV-based SARS-CoV-2-msp-S_F_-Gtc vaccine utilizes the VSV G protein for vaccine production on an industrial scale. VSV G also allows for vector replication following vaccination, which is necessary for effective and prolonged antigen presentation. Our rVSV vectors (described below) contain mutations that we and others have shown to attenuate cytopathogenicity and neurovirulence [20,38,42,50–54]. In our studies herein measuring 24 cytokine/chemokines, human PBMCs exposed to or macaques vaccinated with replicating VSV-based SARS-CoV-2-msp-S_F_-Gtc did not induce considerable PBMC activation or systemic immune activation in non-human primates. Thus, in addition to the vector attenuation and eventual immune clearance, the VSV-based SARS-CoV-2-msp-S_F_-Gtc will induce a robust S-specific immune response but is unlikely to result in systemic immune activation. In addition, our results showed homologous and heterologous boost produce equally robust immune responses. This suggests that anti-vector immunity induced by prime vaccination did not limit the boost induced by homologous rVSV. It is also possible that anti-spike immunity in vaccinated or COVID-19 convalescent individuals could neutralize rVSV infectivity resulting in reduced immunogenicity. However, our results (S6 Fig) indicate that sera containing high levels of anti-spike neutralizing antibodies were unable to neutralize our rVSV-SARS-CoV-2-msp-S_F_-Gtc vaccine. This suggests that anti-spike immunity is unlikely to present a barrier to efficacy of the rVSV vaccine, likely due to sufficient VSV G protein-mediated entry.

We have used avirulent M gene mutants of two different serotypes of VSV for our COVID-19 vaccine development. We have recently demonstrated the efficacy of prime-boost vaccination against the Zika virus [21]. We demonstrated that rVSV_Ind_-ZIKV-E prime vaccination followed by rVSV_NJ_-ZIKV-E boost vaccination induced highly potent neutralizing antibodies and T cell responses, and protected type 1 interferon receptor knockout (*Ifnar*^*-1-*^*)* mice from a lethal dose of the ZIKV challenge. We used the same VSV vectors for this present study. We show that the addition of honeybee melittin signal peptide (msp) and the addition of the transmembrane domain of the VSV G protein (Gtc) to the spike protein of SARS-CoV-2, made pseudotype VSV that carry the spike protein more efficiently, and triggered strong immune responses. In this study we also analyzed the safety of our M gene mutants of VSV_Ind_(GML) and VSV_NJ_(GMM). We found these two M gene mutants are non-cytolytic and non-lethal when we injected highly sensitive type 1 interferon receptor knockout transgenic (*Ifnar*^*-1-*^) mice. All VSV_Ind_(GML) and VSV_NJ_(GMM) injected transgenic mice have survived and had normal weight gain [21].

Regardless of the levels of the S protein derivatives expressed on the rVSV particles, our vaccine is dependent on the quality and quantity of the elicited immune response towards the S protein. In C57BL/6 as well as hACE2-transgenic mice, rVSV expressing the msp-S_F_-Gtc induced the most significant level of anti-S protein antibodies after priming and boosting immunization. Anti-S antibody titers of over 10^5^ and nearly 10^6^ were observed with low (5x10^7^ PFU) and high (5x10^8^ PFU) dose priming and boosting immunizations, which appears to be among the highest reported in mice as compared to other published SARS-CoV-2 vaccines. Likewise, we observed strong IFN-γ lymphocyte responses to pooled S peptides, the highest with the 5x10^8^ PFU priming and boosting immunization dose. The high levels of anti-S antibody response in immunized mice recapitulated in the hACE2 transgenic mice vaccinated with a prime-boost of the rVSV msp-S_F_-Gtc construct. More importantly, this vaccine also provided protection from SARS-CoV-2 challenge as discussed below. We suspect that the high level expression and processing of S_F_ driven by an infectious replication-competent virion in an infected antigen-presenting cell (APC) will lead to good MHC I presentation. Furthermore, because these virus vectors are displaying the S_F_ protein on uptake by APC as well as taking up secreted S_F_ protein, this should lead to cross-presentation of T cell epitopes by APC on MHC I. Thus, this should further enhance a CTL response. The display of S_F_ on the virion, as well as the uptake of secreted S_F_ will also lead to a stronger more efficient MHC II presentation by APC. The CD4 helper responses augment CTL, but more importantly provide robust support for B cell responses that include augmented production of neutralizing antibody, especially after the boost. Of note, the S_F_ protein with the msp and Gtc induced higher neutralizing antibody titers and a higher IFN-γ response when compared with the same S_F_ protein without exogenous signal peptides and transmembrane domain.

A boost with the same vaccine vector can potentially lead to activation of a VSV vector-based immune response that eliminates or reduces effectiveness of the boost. One possibility for the higher efficacy and protection from SARS-CoV-2 with the “unintentional arm” of the Oxford/AstraZeneca vaccine trial may relate to the lower dose SARS-CoV-2 S vaccine used in the priming immunization. The lower dose of priming may have resulted in a reduced anti-vector immune response that in turn did not reduce the boost vaccination with the same adenovirus vector. The Gameleya Institute vaccine (Sputnik V) approach involved the use of adenovirus 5-SARS-CoV-2 S priming immunization followed by a boost with adenovirus 26-SARS-CoV-2 S in attempts to reduce vector-mediated immunity. Published reports suggest that the dual Adenovirus vector approach of Gameleya was more effective at preventing infection than the Oxford/AstraZeneca standard dose priming/standard dose boosting approach (91.6% versus 62.1%) using the higher dose prime/boost [11,55]. Prior to the results of these trials, we optimized S protein antigen expression from a dual VSV serotype vaccine vector system involving rVSV_Ind_ and rVSV_NJ_, different serotypes that do not result in cross neutralization by antibodies. In the vaccination studies of hACE2 transgenic mice, a high titer prime with the rVSV_Ind_ followed by a high titer boost with the rVSV_NJ_ induced the same production of anti-S_F_ binding antibodies and same levels of sera neutralization of SARS-CoV-2 as did prime-boost with just the rVSV_Ind_ expressing the same msp-S_F_-Gtc. Likewise heterologous prime-boost with rVSV_Ind_/rVSV_NJ_ also resulted in the complete survival of the SARS-CoV-2 challenged hACE2 transgenic mice, with limited weight loss, and lacking infectious virus recovery from the lungs, and significantly reduced abnormal pathology in lung tissue. A 10-fold decrease in neutralizing antibodies by reducing the heterologous prime-boost dose (5x10^7^ PFU versus 5x10^8^ PFU) still resulted in complete protection against SARS-CoV-2 challenge. These findings suggest that heterologous versus homologous rVSV prime-boost may not be superior for SARS-CoV-2 protection in hACE2-transgenic mice. However, based on the Gameleya Institute and Oxford/AstraZeneca vaccine Phase III trials, there may be concern of vector-mediated immunity reducing protective efficacy that was not borne out in the small animal studies.

Many countries are experiencing a fourth wave of SARS-CoV-2 infection, mainly due to the delta variant of SARS-CoV-2 and a few vaccine makers have proposed to provide a second boost (third vaccination) in order to protect vaccinees from the variant coronavirus infection. Because our rVSV-SARS-CoV-2-msp-S_F_-Gtc vaccine induces strong neutralizing antibodies, our vaccine can be used as an effective boost vaccine as well after immunization with other vaccines. Sera from prime/boost immunized macaques with VSV-based SARS-CoV-2-msp-S_F_-Gtc showed strong neutralization of the “wild-type” Wuhan SARS-CoV-2, as observed with the sera obtained from vaccinated mice. Since more sera were available from immunized macaques, we also showed strong cross neutralization of the alpha, beta and gamma variants. Studies are now underway with the delta variant. Although there are differences between immunization protocols and immunogens, our prime/boost with rVSV-SARS-CoV-2-msp-S_F_-Gtc had higher levels of binding antibodies to S and higher neutralization titers from sera in mice and macaques than did the prime/boost vaccination studies performed with mice and macaques using the adenovirus vector vaccine, ChAdOx1-S [56]. However, these vaccines were not compared head-on in the same study. Finally, we believe that there would be no problem using our rVSV-SARS-CoV-2-msp-S_F_-Gtc as a boost vaccine following previous vaccination. Our vaccine may be very useful to increase the level of neutralizing antibodies in people who have been vaccinated with other vaccines.

In summary, we have developed a dual rVSV serotype vaccine carrying modified full-length SARS-CoV-2 S protein as a candidate vaccine that induces robust neutralizing antibodies and cell-mediated immune responses towards wild-type SARS-CoV-2 and completely protected vaccinated animals from the SARS-CoV-2 challenge. In addition, homologous rVSV priming and boosting vaccination yielded similar levels of protection from infection compared to heterologous priming, suggesting that vector-induced immunity to the priming vaccination is not a major concern in animals. We believe our research has proven that rVSV-SARS-CoV-2-msp-S_F_-Gtc is a great candidate vaccine, not only for use as a prime and boost vaccination for unvaccinated individuals, but also as a potential boost vaccine following previous vaccination with other COVID-19 vaccines. This vaccine not only increased the immunity of animals from SARS-CoV-2 infection, but also completely protected vaccinated animals from SARS-CoV-2 challenge, increasing the production of neutralizing antibodies approximately tenfold more than what is currently commercially available. In conclusion, we developed rVSV-SARS-CoV-2-msp-S_F_-Gtc to be a dual rVSV serotype vaccine carrying a modified full-length SARS-CoV-2 S protein to induce robust neutralizing antibodies and cell-mediated immune responses towards wild-type SARS-CoV-2 as an effective vaccine candidate in the ongoing fight against the COVID-19 pandemic.

## Materials and methods

### Ethics statement

All procedures for mice vaccination studies were approved by the Institutional Animal Care and Use Committee at the International Vaccine Institute (IACUC Approval No. 2020–018). For non-human primate (NHP) experiments, a formal approval for “Immunogenicity of a VSV based SARS-CoV-2 vaccine in rhesus macaques (protocol 2021–2119 submitted by Andrew Winterborn)” was obtained from Research Ethics Coordinator, University Animal Care Committee, Queen’s University, Kingston, Ontario. Western University REB operates in compliance with, and is constituted in accordance with, the requirements of the Tri-Council Policy Statement: Ethical Conduct for Research Involving Humans (TCPS 2); the International Conference on Harmonisation Good Clinical Practice Consolidated Guideline (ICH GCP); Part C, Division 5 of the Food and Drug Regulations; Part 4 of the Natural Health Products Regulations; Part 3 of the Medical Devices Regulations and the provisions of the Ontario Personal Health Information Protection Act (PHIPA 2004) and its applicable regulations. The REB is registered with the U.S. Department of Health & Human Services under the IRB registration number IRB 00000940. Project ID: 115793, Study Title: In-vitro Analyses of the 2019 novel coronavirus SARS-CoV-2 circulating in London, Ontario for the betterment of drug and vaccine development and to understand CoV-2 pathogenesis Application Type: Continuing Ethics Review (CER) Form.

### Viruses, reagents, and propagation of virus

The following reagents were obtained through BEI Resources, NIAID, NIH: SARS-Related Coronavirus 2, Isolate USA-WA1/2020, NR-52281, contributed by the Centers for Disease Control and Prevention; SARS-Related Coronavirus 2, Isolate hCoV-19/England/204820464/2020, NR-54000, contributed by Bassam Hallis; SARS-Related Coronavirus 2, Isolate hCoV-19/USA/MD-HP01542/2021 (Lineage B.1.351), in *Homo sapiens* Lung Adenocarcinoma (Calu-3) Cells, NR-55282, contributed by Andrew S. Pekosz; and SARS-Related Coronavirus 2, Isolate hCoV-19/Japan/TY7-503/2021 (Brazil P.1), NR-54982, contributed by National Institute of Infectious Diseases.

For SARS-CoV-2 propagation, 10^7^ Vero E6 cells were infected with passage 1 virus of each SARS-CoV-2 variant and “wild-type” SARS-CoV-2 for 3 days. The supernatant was harvested, cell debris was pelleted, and serial dilutions of virus-containing supernatants were used to infect Vero E6 to determine TCID_50_. This passage 2 virus was used in all infections.

### Construction of rVSV-SARS-CoV-2 vaccines

To construct SARS-CoV-2 vaccines using our VSV vectors, we synthetically generated sequences to express the human codon-optimized receptor-binding domain (RBD) plus the envelope (E) protein, the N-terminal containing half of the spike protein (S1 subunit), and the full-length S (S_F_) protein of SARS-CoV-2 (GenBank: JX869059.2) (Genscript USA Inc., Piscataway, NJ, USA). We added honeybee melittin signal peptide (msp) to the amino terminus of the recombinant protein and the VSV G protein transmembrane domain and cytoplasmic tail (Gtc) to the carboxy terminus of some of the recombinant proteins. We independently inserted both modified and unmodified *S*_*F*_, *S1*, and *RBD+*E between the glycoprotein (*G*) and polymerase (*L*) genes of VSV_Ind_ and VSV_NJ_ at *Pme I* and *Mlu I* restriction sites to yield six different recombinant virus constructs for VSV_Ind_ and six for rVSV_NJ_ (Fig 1). We recovered each rVSV virus by VSV reverse genetics and purified the virus with three consecutive rounds of plaque picking and amplification in baby hamster kidney (BHK-21) cells [20]. We previously analyzed the safety of our M gene mutants of VSV_Ind_(GML) and VSV_NJ_(GMM) [20,21]. We found these two M gene mutants are non-cytolytic and non-lethal when we injected highly sensitive type 1 interferon receptor knockout (*Ifnar*^*-1-*^) mice. All VSV_Ind_(GML) and VSV_NJ_(GMM) injected *Ifnar*^*-1-*^ mice survived and had normal weight gain [21].

### Cells and media

We maintained BHK-21 cells (ATCC, CCL-10) in Dulbecco’s Modified Eagle Medium (DMEM, Gibco) containing 5% fetal bovine serum (FBS), 2 mM L-glutamine, 100 units/ml penicillin, and 100 μg/ml of streptomycin. The BHK-21 cell line expressing bacteriophage T7 RNA polymerase, BSR T7/5 cells, was provided by Drs. Buchholz and Conzelmann (Federal Research Center for Virus Diseases of Animals, Germany) [57] and cultured in DMEM containing 5% FBS and 500 μg/ml Geneticin (Gibco). Vero E6 cells were obtained from ATCC (ATCC, CRL-1586) and cultured in Minimum Essential Medium (MEM, Gibco) with 10% FBS, 2 mM L-glutamine, 1 mM sodium pyruvate, 100 units/ml penicillin, and 100 μg/ml of streptomycin.

### Mice and non-human primates

We purchased 6-week-old female C57BL/6 mice from Koatech (Pyeongtaek, Korea) and 6-week-old female human ACE2 Transgenic mice, tg(K18-ACE2)2Prlmn, from The Jackson Laboratory (ME, USA). We housed the mice in a certified A/BSL-3 facility at the International Vaccine Institute. Before the start of each animal experiment, all mice had an acclimation period of one week.

### Western blot analysis

We infected BHK-21 cells with recombinant VSV-SARS-CoV-2 at a multiplicity of infection (MOI) of 6. At six hours post-infection, the supernatant containing the released virions was centrifuged over a 25% sucrose cushion at 150,900 x *g* for 3 hours and resuspended in 100 μl of PBS (1/100 volume of the original volume of culture media). We ran 5 μl of the pelleted virus particles on a SDS-PAGE gel. We also lysed VSV-SARS-CoV-2 infected cells in 100 μl of lysis buffer (10 mM Tris-HCl, pH 7.4, 1% Nonidet P-40, 0.4% Na-deoxycholate, 10 mM Na_2_EDTA). Five μg of the cell lysates were used for the Western blot analysis. SARS-CoV-2 RBD, S1, and S_F_ were detected by a rabbit antibody against SARS-CoV-2 RBD (Sino Biological, 40592-T62). S2 protein was detected by another rabbit antibody against SARS-CoV-2 S2 (Sino Biological, cat# 40590-T62). VSV proteins were detected using rabbit antiserum against lysed VSV_Ind_ or VSV_NJ_. We performed Western blot analysis with an ECL Western blotting analysis kit (GE Healthcare). The protein bands on the PVDF membrane were visualized using a ChemiDoc XRS system (Bio-Rad). E protein was detected by rabbit anti-E antibodies, which were raised by immunizing New Zealand White rabbits with two mixed SARS-CoV-2 envelope protein peptides (GenScript, Peptide 1: NH_2_-MYSFVSEETGTLIVC-COOH, Peptide 2: NH_2_-RVKNLNSSRVPDLLC-COOH). The peptides were conjugated with keyhole limpet hemocyanin (KLH) for the rabbit immunization.

### Analyses of rVSV containing SARS-CoV-2 proteins

We infected BHK-21 cells with rVSV_Ind_ or rVSV_NJ_ expressing the indicated SARS-CoV-2 proteins at an MOI of 3. We incubated cells infected with rVSV_Ind_ at 31°C for 6 hrs and cells infected with rVSV_NJ_ at 37°C for 6 hrs [20]. We centrifuged culture media from the infected cells for 10 minutes at 500 x *g* and filtered through a 0.45-μm filter to remove cell debris. We loaded the filtered culture media onto 1 ml of 25% sucrose cushion and centrifuged at 150,900 x g for 3 hours. We collected the supernatant on top of 25% sucrose to analyze soluble proteins in the media that had not been incorporated into the VSV particles. The collected supernatant was concentrated to 200 μl from a 10 ml original volume using an Amicon Ultra-15 10K molecular weight cut-off size centrifugal filter unit (Merck Millipore Ltd, UFC901024). The pellet was resuspended in 200 μl of PBS and used to analyze proteins incorporated into the VSV particles. SARS-CoV-2 proteins in the cell lysates, concentrated supernatant, and pellets were analyzed by Western blot analysis as described above.

### Immuno-electron microscopy to detect SARS-CoV2 S proteins on the rVSV-SARS-CoV-2 pseudotype particles

Viruses were prepared by infecting BHK-21 cells in three T75 cell culture flasks with an MOI of 0.1 of rVSV_Ind_, rVSV_Ind_-msp-S_F_-Gtc, rVSV_NJ_, and rVSV_NJ_-msp-S_F_-Gtc separately. BHK-21 cells infected with rVSV_Ind_ and rVSV_Ind_-msp-S_F_-Gtc were incubated at 31°C. BHK-21 cells infected with rVSV_NJ_, rVSV_NJ_-msp-S_F_-Gtc were incubated at 37°C. The culture media was collected following 20 hrs of infection, and centrifuged at 2,756 x *g* for 5 minutes. The supernatant was filtered through a 0.4 μm pore size syringe filter to remove the cellular debris. We fixed the viruses by treating them with 0.1% glutaraldehyde (Merk, EM grade, 25% solution) for 1 hr. We concentrated the viruses by ultra-centrifugation of the collected culture media through the 25% sucrose cushion for 3 hrs at 110,880 x *g* at 4°C. The pelleted viruses were resuspended in 200 μl of TNE buffer (10 mM Tris-HCl, pH 7.4, 100 mM NaCl, 1 mM EDTA). Ten μl of virus samples were applied onto nickel grids (FCF 400-Ni-TC, Electron Microscopy Sciences). They were kept on the grid for 5 minutes, and extra samples were removed from the grid with filter paper. The samples were dried for 5 minutes. To inactivate residual aldehyde groups present after aldehyde fixation, grids were incubated on 0.05 M glycine in a PBS buffer for 15 minutes. The grids were washed on drops of incubation solution (PBS w/ 0.1% BSA-C) for 2 x 5 minutes. The samples were treated with 10 μl of primary antibodies that were diluted 10-fold with PBS w/ 0.1% BSA-C. Mouse anti-VSV_Ind_ G (Kerafast, EB0010) was used for the VSV G protein. Rabbit anti-SARS-CoV-2 S (Sino Biological, 40592-T62) was used for SARS-CoV-2 S. They were incubated for 1 hour at room temperature. The grids were washed with drops of incubation solution six times for 5 minutes each. The samples were treated with an appropriate gold conjugate reagent for 2 hrs. The gold conjugate reagent was diluted tenfold in the incubation solution. Goat-anti-mouse IgG (Electron Microscopy Sciences, #25133) was used for anti-VSV G treated samples. Goat-anti-rabbit IgG (Electron Microscopy Sciences, #25113) was used for anti-SARS-CoV-2 S treated samples. The grids were washed with drops of incubation solution six times for 5 minutes each. The grids were washed two additional times with TNE buffer for 5 minutes each. The samples were stained with 0.5% phosphotungstic acid (Sigma-Aldrich, HT152-250ML) for 30 seconds and were examined with the transmission electron microscope and an imaging system (Philips CM10).

### VSV-SARS-CoV-2 vaccine preparations by anion exchange chromatography

BHK-21 cells grown in T75 flasks were infected with an MOI of 0.1 rVSV-SARS-CoV-2 for 18 hrs in the 31°C incubator for rVSV_Ind_-SARS-CoV-2 and in the 37°C incubator for rVSV_NJ_-SARS-CoV-2. To remove cellular debris, the culture medium was centrifuged at 4,500 rpm for 5 minutes, and the supernatant was filtered through a 0.45 μm pore size bottle-top filter. The viruses were mixed with 10X SPG buffer to have 2% sucrose, 10 mM potassium phosphate, 0.00376 M KH_2_PO_4_, 0.0071 M K_2_HPO_4_, 5 mM glutamate L-glutamic potassium salt monohydrate. The same volume of HNS buffer (10 mM HEPES, 0.465 M NaCl, 2% sucrose) was mixed to prepare the final volume of conditioned virus samples. The conditioned virus samples were purified by anion exchange chromatography using NGC Chromatography System (Bio-Rad) and Mustang Q XT5 membrane column (Pall Canada). The viruses were eluted using a salt gradient formed by mixing equilibration buffer (low salt buffer, pH 7.5: 10 mM HEPES, 0.29 M NaCl, 2% sucrose) and 0% to 70% of elution buffer (high salt buffer, pH 7.0: 10 mM HEPES, 1.5 M NaCl, 2% sucrose). The eluted virus samples were buffer-exchanged with PBS. They were concentrated to about 1 ml volume using a Centricon Plus-70 centrifugal filter device (10K MW cut-off, Millipore). The concentrated viruses were mixed with freezing buffer to have 4% sucrose and 1% human serum albumin or 1% bovine serum albumin. Virus aliquots were prepared and stored at -80°C. Virus titers were determined by plaque assay prior to vaccination.

### Immunization and virus challenge

For immune response studies, we prime-immunized five C57BL/6 mice (six weeks old) per group with rVSV_Ind_ constructs and boost-immunized with rVSV_NJ_ or rVSV_Ind_ constructs two weeks after prime immunization. Immunizations were performed intramuscularly on the hind leg. Mice were vaccinated with two different doses, either 5x10^7^ PFU or 5X10^8^ PFU. Two weeks after each immunization, we collected blood from the retro-orbital plexus, and isolated serum from the clotted blood after centrifugation. We incubated serum at 56°C for 30 minutes and then stored it at -80°C until further analysis. For the challenge studies, we prime- and boost-immunized ten 6-week-old female hACE2 transgenic mice [tg(K18-ACE2)2Prlmn] per group intramuscularly with 5x10^8^ PFU or 5x10^7^ PFU of rVSV-msp-S_F_-Gtc 14 days apart. We collected mouse sera after 14 days of each immunization under anesthesia. We challenged mice four weeks after the last immunization with 1x10^5^ PFU of SARS-CoV-2 (S clade, National Culture Collection for Pathogens (NCCP #43326 Korea Disease Control and Prevention Agency) in a 50 μl volume intranasally under anesthesia. We monitored the survival and body weight of each mouse daily.

### Enzyme-linked immunosorbent assay (ELISA)

We determined the SARS-CoV-2 spike protein-specific IgG levels in mouse sera by indirect ELISA. We coated a 96-well ELISA plate (Thermo Fisher Scientific, Waltham, MA, USA) with 200 ng of S-ΔTM (LakePharma, CA, USA). We washed the plates with PBST (PBS with 0.05% tween-20) three times and then incubated with blocking buffer (PBS with 2% BSA and 0.05% tween-20) for 1 h at 37°C. We serially diluted serum samples five-fold, starting with a 1:30 dilution in the blocking buffer. We added the diluted serum to the plate and incubated it for two hrs at 37°C and then washed it three times with PBST. We incubated the plates with 1:3000 diluted HRP-conjugated goat anti-mouse IgG (Southern Biotech, Birmingham, AL, USA) for 1 h at 37°C and then washed with PBST. We added peroxidase substrate (TMB) solution (Millipore, Billerica, MA, USA) and incubated it for 3 to 5 minutes at room temperature. We stopped the reaction by adding 0.5 N HCl (Merck, Darmstadt, Germany) and OD values were measured at 450 nm using an ELISA plate reader (Molecular Devices, San Jose, CA, USA). We expressed antibody titer as a reciprocal log_10_ titer of serum dilution showing an OD value of 0.2.

### Focus Reduction Neutralization Test 50 (FRNT_50_) assays using animal sera

For focus reduction assays, immune sera were heat-inactivated at 56°C for 30 minutes and then serially diluted 2-fold in 25 μl of DMEM with 2% FBS. We mixed the diluted sera with 500 PFU of wild-type SARS-CoV-2 (S clade, NCCP. 43326, Korea Disease Control and Prevention Agency), in 25 μl per well. We incubated the serum-virus mixture at 37°C for 30 minutes, after which we added 50 μl of the mixture to Vero cells in a 96-well microplate. We incubated the infected cells at 37°C with 5% CO_2_ for 4 hours and then fixed them with 4% formaldehyde. We treated cells one day after fixation with cold 100% methanol for 10 minutes to increase permeability. We added 100 μl of blocking buffer (1% BSA, 0,5% goat serum, 0.1% tween-20 in PBS) to the wells and mixed the cells with a 1:3000 dilution of primary anti-SARS-CoV-2 NP rabbit mAb (43143-R001, Sino Biological, PA, USA). We incubated the cells at 37°C for 1 hour. We washed the cells three times with 200 μl of washing buffer (0.1% tween-20 in PBS). We then added goat anti-rabbit IgG-HRP secondary antibody (1721019, Bio-Rad, CA, USA) diluted 1:2000 in blocking buffer to the cells and incubated them for 1-hour at 37°C. We washed the cells three times with wash buffer and added 30 μl of KPL TrueBlue Peroxidase Substrate (Seracare, MA, USA) to the cells for 30 minutes at room temperature. We then counted the developed foci with a spot reader (ImmunoSpot S5, CTL). We counted the number of foci developed in the presence or absence of the diluted serum and then we determined the highest serum dilutions resulting in a 50% reduction in the number of foci.

For measuring NAb in sera of NHP immunized with prime-boost with rVSV-SARS-CoV-2-msp-S_F_-Gtc, sera were heat-inactivated as described above. Vero E6 cells were plated at 15,000/well two days prior to assay in a 96-well plate. At 48 hours post-seeding, the sera were serially diluted two-fold starting at 1:20. Diluted sera were incubated with 60 PFU SARS-CoV-2-Nano-Luciferase (nLuc) [58]; or 100 PFU of SARS-CoV-2 wild type, SARS-CoV-2 Alpha variant, SARS-CoV-2 Beta variant, or SARS-CoV-2 Gamma variant in a 96-well round-bottom plate (serum + virus mix) at a total of 100 ul and incubated at 37°C/5% CO_2_. After 1 hour, media was removed from the Vero E6 cells and 100 μl of the incubated serum + virus mix was added to the appropriate wells in duplicate (nLuc) or quadruplicate for SARS-CoV-2 variants. Cells were incubated with serum + virus for 1 hour with agitation every 10 min. SARS-CoV-2 variant infections were then incubated for 48 h at 37°C/5% CO_2_ prior to processing for qRT-PCR. Media + virus mixture was removed from SARS-CoV-2 nLuc infected cells and replaced with DMEM + 2% FBS then incubated for 24 hours at 37°C/5% CO_2_ until luciferase readout.

For readout of the SARS-CoV-2-nLuc neutralization assay, media was removed from cells and replaced with 50 μl PBS. An equal volume of 50 μl Nano Luciferase Assay Substrate (Promega) was added to each well and mixed before transfer to a black 96-well plate. Luciferase signal was measured using Synergy LX multi-mode reader (BioTek) following the manufacturer’s instructions. Neutralization was indicated by greater than 50% reduction in luciferase signal compared to serum from the same animals prior to vaccination (non-immune controls).

For the neutralizing assays using SARS-CoV-2 wild-type and variants, qRT-PCR was performed on the viral RNA released into the supernatant from the cells exposed to the sera as described below.

### SARS-CoV-2 viral RNA extraction and qRT-PCR analysis

All RNA extracts were collected using a QIAamp 96 Viral RNA Kit (Qiagen, MD, US) following the manufacturers protocol with centrifugations performed on an Allegra X-14R Benchtop Centrifuge (Beckman Coulter, Brea, CA, US). First, 560 μl of lysis buffer (AVL) containing carrier RNA was added to 80 μl of sample, thoroughly mixed, and allowed to incubate for 10 minutes at room temperature. 560 μl of 100% ethanol was then added to each sample. The lysate was added to the QIAamp 96-well plate and centrifuged at 4000 x g for 4 minutes. The remaining lysate was added in a subsequent round of centrifugation to ensure all lysate had passed through the column for maximum RNA binding. The samples were then washed with 500 μl of buffer AW1 and AW2 followed by a 10-minute drying spin. Finally, the bound RNA was eluted in 80 μl of buffer AVE. All RNA extracts were stored at -80°C for qRT-PCR analysis. All reactions were performed on a QuantStudio 5 Real-Time PCR system (Applied Biosystems, Waltham, MA, US) with TaqMan Fast Virus 1-Step reagents (Applied Biosystems, Waltham, MA, US) following the manufacturer’s protocol. A final reaction volume of 10 μl containing 2.5 μl of template was used under the following thermocycling conditions: cDNA synthesis (50°C / 5 minutes); a hold step (95°C / 20 seconds); 45 cycles of denaturation (95°C / 15 seconds) and annealing/elongation (60°C / 40 seconds). The primer-probe pair used for N gene detection are nCOV_N1 Forward Primer (5´-GACCCCAAAATCAGCGAAAT-3´), nCOV_N1 Reverse Primer (5´-TCTGGTTACTGCCAGTTGAATCTG-3´), and nCOV_N1 Probe FAM-(5´-ACCCCGCATTACGTTTGGTGGACC-3´)-BHQ1. Nuclease-free water was used as a negative control.

### ELISpot and MAGPIX cytokine/chemokine assay

To measure the cytokine production capacity of T cells after vaccination, we conducted an IFN-γ ELISpot assay using the Mouse IFN-γ ELISpot Kit (BD Biosciences, San Jose, CA, USA) according to the manufacturer’s instructions. Briefly, we removed the spleen from each mouse two weeks after boost immunization. We then obtained cellular suspensions of splenocytes by passing spleens through a 70-μm cell strainer (BD Bioscience, San Jose, CA, USA). We removed red blood cells by hemolysis using ACK Lysing Buffer (Thermo Fisher Scientific, Waltham, MA, USA). We then added 2x10^5^ cells into pre-coated BD ELISPOT plates in the presence or absence of stimulatory peptides for 16 hours at 37°C in a 5% CO_2_ incubator. We then used a pool of SARS-CoV-2 S protein-specific peptides (S7 Fig; PepTivator SARS-CoV-2 Prot_S (sequence domains aa 304–338, 421–475, 492–519, 683–707, 741–770, 785–802, and 885–1273; 15-mer sequences with 11 amino acids overlap), Cat # 130-126-701, Miltenyi Biotec, Bergisch Glasdbach, North Rhine-Westphalia, Germany. An HIV-1 Gag peptide (AMQMLKETI) was used as a negative control for non-specific stimulation. After stimulation, we discarded cells and peptides and soaked each well in distilled water for 3–5 minutes. After washing three times with Washing Buffer (BD Bioscience, San Jose, CA, USA), we added 100 μl of diluted detection antibody [Biotinylated anti-mouse IFN-γ (BD Bioscience, San Jose, CA, USA)] in Assay Diluent (BD Biosciences, San Jose, CA, USA) to each well and incubated for 2 hours at RT. After washing, we added 1:100 diluted streptavidin-HRP (BD Bioscience, San Jose, CA, USA) in Assay Diluent to each well and then incubated plates within 1 hour at room temperature. We washed the plates four times with Wash Buffer and then with PBS twice. After washing, we added 1:50 diluted AEC chromogen with AEC Substrate Buffer (BD Bioscience, San Jose, CA, USA) to each well. We stopped the spot development by washing with distilled water in each well. After air-drying the plates, we used an ELISpot reader (Cellular Technology Limited, Cleveland, OH, USA) to count the number of spots. ConA was used as positive controls in ELISpot assays.

We used a multiplex instrument MAGPIX to quantify the cytokine/chemokine profiles within the serum of VSV-SARS-CoV-2 vaccinated NHP and cytokine/chemokine profiles in the cell culture supernatant of VSV-SARS-CoV-2 infected human PBMCs. Serum samples or cell culture supernatant samples were prepared following the manufacturer’s instructions (Milliplex MAP kit, Millipore) and 25 μl of undiluted samples was added to 25 μl of assay buffer. Then, 25 μl of magnetic beads coated with specific antibodies (HT17MG-14K-PX25 combined with HIL18MAG-66K, Milliplex MAP Kit, Millipore) was added to this solution and the reaction was incubated at 4°C for 16 h. Next, the beads were washed and incubated with 25 μl of biotinylated detection antibody at room temperature (RT) for 1 hour. To complete the reaction, 25 μl of Streptavidin–Phycoerythrin conjugate compound was added and allowed to incubate for 30 min at RT. The beads were then washed and incubated with 50 μl of sheath fluid for 5 min at RT. The samples were analyzed on MAGPIX instruments. The concentration of the analytes was then determined by MAGPIX xPONENT software. The assays were run in duplicate to confirm the results. Analytes were normalized to total protein concentration.

Twenty six analytes were studied: interleukin 1 beta (IL-1β), interleukin-2 (IL-2), interleukin-4 (IL-4), interleukin-5 (IL-5), interleukin-6 (IL-6), interleukin-9 (IL-9), interleukin-10 (IL-10), interleukin-12p70 (IL-12p70), interleukin-13 (IL-13), interleukin-15 (IL-15), interleukin-17A (IL-17A), interleukin-17E/ interleukin-25 (IL-17E/25), interleukin-17F (IL-17F), interleukin-18 (IL-18), interleukin-21 (IL-21), interleukin-22 (IL-22), interleukin-23 (IL-23), interleukin-27 (IL-27), interleukin-28A (IL-28A), interleukin-31 (IL-31), interleukin-33 (IL-33), granulocyte-macrophage colony-stimulating factor (GM-CSF), interferon gamma (IFNγ), macrophage inflammatory protein 3 alpha (MIP-3α), tumor necrosis factor alpha (TNFα), and tumor necrosis factor beta (TNF β).

### Viral load assay

To examine SARS-CoV-2 viral loads in the challenged transgenic mice, we aseptically removed the lungs from each mouse and washed them with Hank’s balanced salt solution (HBSS, Thermo Fisher Scientific, MA, USA) containing 1% penicillin/streptomycin (Thermo Fisher Scientific, MA, USA). We homogenized the lung and strained it through a 70 μm cell strainer (Becton Dickinson, NJ, USA). We collected the supernatant and stored it at -80°C. We serially diluted the lung samples four fold in DMEM and added it to Vero E6 cells in a 12-well plate. After rocking the plate back and forth for 30 min, we removed the inoculum from the plate and added agar-overlay media (DMEM with 1% (w/v) low-melting agarose (Lonza, Basel, Switzerland) and 2% FBS. We incubated the plates at 37°C for three days until plaques developed. We then fixed the cells with 4% (v/v) formaldehyde solution for 30 minutes, rinsed them with water, and stained with 0.05% crystal violet solution (Sigma, Darmstadt, Germany) for five minutes. Then we rinsed the plates with water and counted the number of plaques per well.

### Histopathology of the lungs from the virus challenged mice

We fixed the left lobe of each mouse lung in 10% buffered formalin. We then processed and embedded the lung tissues in low-melting paraffin, sectioned to a thickness of 3 μm, and stained with hematoxylin and eosin. We examined the stained tissues under a light microscope (Olympus CS41, Japan) and graded lesions in the tissues semi-quantitatively depending on their severity. SARS-CoV-2 infection-related inflammatory lesions included inflammatory foci, characterized by infiltration of inflammatory cells around blood vessels and interstitial alveolar inflammation. In the cases of inflammatory foci, we graded inflammation based on the following criteria: 1) cases with very weak cell infiltration, close to normal, but there is recognizable inflammatory cell infiltration around some vessels (inflammatory score = 0.5), 2) some blood vessels with mild perivascular inflammatory cell infiltration in the lung section (minimal, inflammatory score = 1), 3) some blood vessels in the lung section with prominent infiltration of inflammatory cells (mild, inflammatory score = 2), 4) more than 50% of blood vessels in the lung section have prominent perivascular infiltration of inflammatory cells (moderate, inflammatory score = 3), 5) most blood vessels in the lung section have prominent and heavy perivascular infiltration of inflammatory cells (severe, Inflammatory score = 4). We scored the level of inflammation in each case by combining the two different inflammation scores of inflammatory foci and alveolitis. We then calculated the mean inflammatory score of each group from the individual inflammatory scores.

## Supporting information

S1 Fig Expression of SARS-CoV-2 proteins from rVSV_NJ_-SARS-CoV-2. To check the expression of SARS-CoV-2 RBD, S1, and S_F_ from rVSV_NJ_-SARS-CoV-2 infected cells, BHK-21 cells were infected with the virus at an MOI of 6. After six hours incubation at 37°C, cell lysates were prepared and protein expression was determined by Western blot. Cell lysates were loaded in 5 μg quantity for SDS-PAGE. RBD, S1, and S_F_ proteins were detected by rabbit antibody against SARS-CoV-2 RBD. S2 protein was detected by rabbit antibody against SARS-CoV-2 S2. E protein was detected by rabbit antibody against SARS-CoV-2 E peptides. **(A)** Expression of RBD, S1, and S_F_ with and without msp and Gtc. **(B)** Expression of S2 with and without Gtc. **(C)** Expression of E protein. **(D)** Expression of VSV_NJ_ N, P, M, and G proteins. Purple box: honeybee msp, red box: VSV Gtc.
(TIF)

S2 Fig SARS-CoV-2 RBD, S1, and S_F_ from recombinant VSV are highly glycosylated. To assess protein glycosylation, 20 μg of infected cell lysates from rVSV_Ind_(GML) infection (Fig 2) were treated with 10 units of Peptide N-Glycosidase F (PNGase F, Sigma-Aldrich, G5166) and incubated at 37°C for 3 hrs. according to the manufacture’s protocol. The migratory pattern of the proteins was examined by Western blot analysis. Five μg of the PNGase F treated and untreated cell lysates were loaded on the SDS-PAGE. RBD, S1, and S_F_ were detected by an antibody against SARS-CoV-2 RBD and S2 was detected by an antibody against SARS-CoV-2 S2. **(A)** Detection of RBD, S1, and S_F_ proteins with and without Gtc in the PNGase F untreated (-) and treated (+) cell lysates. **(B)** Detection of S2 and S_F_ proteins with and without Gtc in the PNGase F untreated (-) and treated (+) cell lysates. Purple box: honeybee msp, red box: VSV Gtc.
(TIF)

S3 Fig Incorporation of S1, S2, S_F_, and RBD proteins with VSV Gtc into rVSV_NJ_. Incorporation of SARS-CoV-2 S1, S2, S_F_, and RBD with or without VSV Gtc into rVSV_NJ_ particles was examined by infecting BHK-21 cells with rVSV_NJ_-SARS-CoV-2 at an MOI of 3. The rVSV_NJ_-SARS-CoV-2 infected cells were incubated at 31°C for 6 hrs. Infected cell lysates were prepared in lysis buffer (lanes 1, 2, and 5). Culture media from the infected cells was centrifuged at 500 x g for 10 minutes and supernatant was filtered through a 0.45 μm filter to remove cell debris. The filtered culture media was loaded onto 1 ml of 25% sucrose cushion and ultra-centrifuged at 150,900 x g for 3 hrs. Supernatant on top of the 25% sucrose cushion was collected to check the soluble proteins in the media (lanes 3 and 6). Pelleted samples were checked for proteins incorporated into VSV particles (lanes 4 and 7). We detected RBD, S1, and S_F_ proteins by Western blot using an antibody against SARS-CoV-2 RBD protein. S2 and S_F_ proteins were detected by the rabbit antibody against SARS-CoV-2 S2. **(A)** Detection of S_F_ and S1 proteins in cell lysate, concentrated culture media, and virus pellet from cells infected with rVSV_NJ_-msp-S_F_-Gtc or rVSV_NJ_-S_F_. **(B)** Detection of S_F_ and S2 proteins in cell lysate, concentrated culture media, and virus pellet from cells infected with rVSV_NJ_-msp-S_F_-Gtc or rVSV_NJ_-S_F_. **(C)** Detection of VSV_NJ_ proteins in cell lysate, concentrated culture media, and virus pellet from cells infected with rVSV_NJ_-msp-S_F_-Gtc or rVSV_NJ_-S_F_. **(D)** Detection of S1 protein in cell lysate, concentrated culture media, and virus pellet from cells infected with rVSV_NJ_-msp-S1-Gtc or rVSV_NJ_-S1. **(E)** Detection of RBD proteins in cell lysate, concentrated culture media, and virus pellet from cells infected with rVSV_NJ_-msp-RBD-Gtc+E-Gtc or rVSV_NJ_-msp-RBD+E. **(F)** Detection of VSV_NJ_ proteins in cell lysate, concentrated culture media, and virus pellet from the cells infected with rVSV_NJ_-msp-RBD-Gtc+E-Gtc or rVSV_NJ_-msp-RBD+E. Purple box: honeybee msp, red box: VSV Gtc.
(TIF)

S4 Fig Immuno-electron microscopy to detect SARS-CoV2 S protein on the VSV-SARS-CoV-2 particles. The recombinant VSVs, rVSV_Ind_, rVSV_Ind_-msp-S_F_-Gtc, rVSV_NJ_, and rVSV_NJ_-msp-S_F_-Gtc were prepared as described in materials and methods. Ten μl of virus samples were applied onto the Nickel grids (FCF 400-Ni-TC, Electron Microscopy Sciences). The samples were treated with 10 μl of blocking solution (PBS w/ 2% BSA-C, Electron Microscopy Sciences) for 15 minutes. The grids were washed on drops of incubation solution (PBS w/ 0.1% BSA-C) for 2 x 5 minutes. The samples were treated with 10 μl of tenfold diluted primary antibodies, which were diluted with PBS w/ 0.1% BSA-C. Mouse anti-VSV_Ind_ G was used for VSV G protein. Rabbit anti-SARS-CoV-2 S was used for SARS-CoV-2 S. The samples were treated with an appropriate gold conjugate reagent for 2 hrs. The gold conjugate reagent was diluted tenfold in the incubation solution. Goat-anti-mouse IgG-gold was used for anti-VSV G treated samples. Goat-anti-rabbit IgG-gold was used for anti-SARS-CoV-2 S treated samples. The samples were stained with 0.5% phosphotungstic acid for 30 seconds. The samples were examined with the transmission electron microscope and an imaging system (Philips CM10). **(A)** rVSV_Ind_ labeled with an antibody against VSV_Ind_ glycoprotein antibody. **(B)** rVSV_Ind_ labeled with an antibody against RBD of SARS-CoV-2 spike protein. **(C)** rVSV_Ind_-msp-S_F_-Gtc labeled with an antibody against VSV_Ind_ glycoprotein antibody. **(D)** rVSV_Ind_-msp-S_F_-Gtc labeled with an antibody against RBD of SARS-CoV-2 spike protein. **(E)** rVSV_NJ_ labeled with an antibody against RBD of SARS-CoV-2 spike protein. **(F)** rVSV_NJ_-msp-S_F_-Gtc labeled with an antibody against RBD of SARS-CoV-2 spike protein.
(TIF)

S5 Fig Full-length S_F_ protein with modifications (rVSV-msp-S_F_-Gtc) induces the strongest neutralizing antibody titer against SARS-CoV-2. Mice were immunized and sera were collected as described in Fig 5. SARS-CoV-2 neutralization was determined by FRNT_50_ assay as has been described in Fig 6. Statistical significance was determined by two-way ANOVA with Tukey’s correction (*, p < 0.05; ns, not significant). The data were presented as means with error bars of standard deviation. Purple box: honeybee msp, red box: VSV Gtc.
(TIF)

S6 Fig Recovered COVID-19 patients’ sera do not neutralize the rVSV-msp-S_F_-Gtc. We diluted rabbit serum against VSV_Ind_ and recovered COVID-19 patients’ sera twofold serially starting at 1: 40 dilutions to 1: 40,960 dilutions. The reciprocal titer of all five COVID-19 patients’ sera for the 50% neutralization of SARS-CoV-2-Wuhan strain was >1/640. SARS-CoV-2 neutralizing antibody (MBS9141964, MyBioSource.com), which 50% inhibitory concentration is 0.496μg/mL, was used as a positive neutralization antibody against SARS-CoV-2. SARS-CoV-2 neutralizing antibody (MBS9141964, MyBioSource.com) was diluted two-fold serially starting at 100 μg/ml concentrations to 0.1 μg/ml concentrations. rVSV_Ind_ and rVSV_Ind_-msp-S_F_-Gtc were diluted to 2X10^4^ PFU/ml, and 80 μl of the diluted VSV and 80 μl of the diluted antisera were mixed and incubated at 37°C for 1hr. Fifty μl of the VSV and antisera mixture was added to HEK293-T or HEK293-T-hACE2 cells in 96 well cell-culture plates for the adsorption of 1 hr. We added 150 μl of complete DMEM to the cells and incubated for three days to determine the antibody titer to neutralize 100% of the rVSV_Ind_ and rVSV_Ind_-msp-S_F_-Gtc in HEK293-T or HEK293-T-hACE2 cells. **(A)** Neutralization antibody titers of rabbit anti-VSV_Ind_, recovered COVID-19 patients’ sera, and SARS-CoV-2 neutralization antibody against rVSV_Ind_. **(B)** Neutralization antibody titer of rabbit anti-VSV_Ind_, recovered COVID-19 patients’ sera, and SARS-CoV-2 neutralization antibody against rVSV_Ind_-msp-S_F_-Gtc. **(C)** Reciprocal neutralizing antibody titers.
(TIF)

S7 Fig SARS-CoV-2 S protein-derived peptides for ELISPOT assays. A pool of peptides (in bold and underlined) were used to stimulate T cells.
(TIF)

S8 Fig Vaccination of hACE2 transgenic mice with rVSV_Ind_-msp-S_F_-Gtc is protective from higher dose SARS-CoV-2 challenge. Six-week-old female hACE2 transgenic mice were prime-vaccinated with rVSV_Ind_-msp-S_F_-Gtc (n = 9) or rVSV_Ind_ (n = 4) and boost vaccinated with rVSV_Ind_-msp-S_F_-Gtc or rVSV_Ind_ two weeks after prime-vaccination. Four weeks after boost-vaccination (S6 Table), mice were challenged intranasally with 5X10^5^ PFU SARS-CoV-2. Body weight and survival of each mouse were monitored daily. **(A)** Individual body weight for mice vaccinated with rVSV_Ind_-Mock and challenged with SARS-CoV-2. **(B)** Individual body weight for mice vaccinated with rVSV_Ind_-msp-S_F_-Gtc and challenged with SARS-CoV-2. **(C)** SARS-CoV-2 viral loads in the lungs of vaccinated and challenged hACE2 transgenic mice. Right lobes of mice lungs were aseptically removed from the mice on day 3 and day 7 after the SARS-CoV-2 challenge. Infectious SARS-CoV-2 was quantified by plaque assay on Vero E6 cells. Statistical significance was determined by two-way ANOVA with Tukey’s correction (**, p< 0.005). **(D)** Mouse survival after the SARS-CoV-2 challenge.
(TIF)

S9 Fig Pictures of lungs from hACE2 mice, which were vaccinated with rVSV_Ind_-msp-S_F_-Gtc or rVSV_Ind_ and challenged with SARS-CoV-2. Six-week-old female hACE2 transgenic mice were prime-vaccinated with rVSV_Ind_-msp-S_F_-Gtc (n = 9) or rVSV_Ind_ (n = 4) and boost vaccinated with rVSV_Ind_-msp-S_F_-Gtc or rVSV_Ind_ two weeks after prime-vaccination. Four weeks after boost-vaccination (S6 Table), mice were challenged intranasally with 5X10^5^ PFU of SARS-CoV-2. Two mice from the rVSV_Ind_-Mock vaccinated groups and three mice from rVSV_Ind_-msp-S_F_-Gtc vaccinated group were euthanized on day 3 and on day 7 after SARS-CoV-2 challenge to check the virus loads in the lung. Before isolation of the SARS-CoV-2 from the infected lungs, pictures were taken. We took pictures of the ventral and dorsal sides of each lung.
(TIF)

S10 Fig Neutralization of wild-type and variants of concern (VOCs) SARS-CoV-2 by a monoclonal anti-S protein antibody and by sera from macaques prime/boost immunized with rVSV-msp-SARS-CoV-2-Gtc. For measuring neutralization by the monoclonal Sino-R001 NAb and in sera of macaques immunized with prime-boost with rVSV-msp-SARS-CoV-2-Gtc, monoclonal Nab and sera from macaque B was serially diluted two-fold starting at 1:40 and added to Vero E6 cells along with 100 plaque-forming units (PFU) of SARS-CoV-2 wild type (panel **A** for NAb and panel **E** for Rhesus macaque **B**), SARS-CoV-2 Alpha variant (panel **B** and **F**), SARS-CoV-2 Beta variant (panel **C** and **G**), or SARS-CoV-2 Gamma variant (panel **D** and **H**). The monoclonal Sino-R001 NAb was only diluted to 1/640. All assays were performed in quadruplicate. Virus production was measured by qRT-PCR as described in the materials and methods, converted to % neutralization based on maximal replication in absence of sera. In panels E through H, the level of neutralization of heat-inactivated preimmunization sera from macaque B was also run and shown on the graph for only the 1/40, 1/80, and 1/160 dilutions.
(TIF)

S1 Table Determination of the ratio of total virus particle (as measured by VSV gRNA) to infectious virus particle (as measured by plaque-forming units). The purified VSV-SARS-CoV-2 were diluted to 5X10^7^ PFU in 200 μl volume. Fifty μl of the diluted viruses was titrated for the infectious virus particles by plaque assay, and 140 μl of the virus was used for genomic RNA isolation. VSV genomic RNA was isolated by using QIAamp Viral RNA kit (Qiagen, Cat. No. 52904). The extracted RNA was resuspended in 60 μl elution buffer. Ten μl of the isolated RNA was used for reverse transcription by SuperScript IV Reverse Transcriptase kit (Invitrogen, Cat. No. 18090200) and yield 20 μl cDNA product. Two μl of the cDNA was used as templates for the qPCR. The SARS-CoV-2 S gene specific qPCR primers and a probe were as follow: Forward primer: 5´-GCCCAGGTGAAGCAAATCTA-3´; Revers primer: 5´-GAACAGCAGGTCCTCGATAAAG-3´; Probe: 5´-/56-FAM/CTGCCTGATCCATCCAAG CCTTCT/3IABkFQ-3´. The qPCR was carried out with QuantStudio 5 real time PCR system (Applied biosystems). The DNA plasmid, pVSV_Ind_-COV-2 msg-S_F_-Gtc was used as a copy number standard and the five different concentrations of the plasmid for the standard curve were 5×10^7^ copies, 5×10^6^ copies, 5×10^5^ copies, 5×10^4^ copies, 5×10^3^ copies.
(TIF)

S2 Table Vaccination with rVSV-SARS-CoV-2 in mice. To analyze humoral immune responses towards SARS-CoV-2 S_F_, S1 and RBD, we vaccinated C57BL/6 mice (n = 5/vaccination group) intramuscularly with rVSV vaccine vectors at 5x10^7^ PFU/mouse or 5x10^8^ PFU/mouse. We prime vaccinated each mouse with rVSV_Ind_ constructs. Two weeks after prime immunization, we boost-immunized the mice with rVSV_NJ_ constructs.
(TIF)

S3 Table Neutralization of the boost vaccines by mice immune sera after the prime vaccination. Mouse immune sera against VSV-SARS-CoV-2 msp-S_F_-Gtc were prepared by vaccinating interferon α and β receptor knock out mice (Ifnar-/- mice, Jackson Laboratory, B6.129S2-*Ifnar1*^*tm1Agt*^/Mmjax) once with 5X10^8^ PFU of rVSV_Ind_-msp-S_F_-Gtc. The immune serum was collected at 28 days after the vaccination. For neutralization, 5X10^7^ PFU of rVSV_Ind_-msp-S_F_-Gtc or rVSV_NJ_-msp-S_F_-Gtc in 100 μl were mixed with 100 μl of serum from PBS injected mice (n = 2) or with 100 μl of serum from VSV-SARS-CoV-2 msp-S_F_-Gtc vaccinated mice (n = 3). The virus and serum mixture was incubated for one hour at 37°C. The titer of viruses in the mixture was determined by plaque assay. **(A)** Neutralization of VSV-SARS-CoV-2-msp-S_F_-Gtc by undiluted immune sera. **(B)** Neutralization of VSV-SARS-CoV-2-msp-S_F_-Gtc by diluted immune sera.
(TIF)

S4 Table Neutralization titers of SARS-CoV-2-nLuc and SARS-CoV-2 variants of concern with sera of macaques prime-boost vaccinated with rVSV-msp-SARS-CoV-2-Gtc. Three macaques were prime-immunized intramuscularly with 10^9^ PFU rVSV_Ind_-SARS-CoV-2-msp-S_F_-Gtc and boost-immunized with 10^9^ PFU of rVSV_Ind_-SARS-CoV-2-msp-S_F_-Gtc 20 days after the prime-immunization. For measuring NAb in the sera of NHP, Vero E6 cells were seeded at 15,000 cells/well in a 96-well plate. At 48 hours post-seeding, the sera were serially diluted two-fold starting at 1:20. Diluted sera were incubated with 60 PFU SARS-CoV-2-Nano-Luciferase (nLuc) [58]; or 100 PFU of SARS-CoV-2 wild-type, SARS-CoV-2 Alpha variant, SARS-CoV-2 Beta variant, or SARS-CoV-2 Gamma variant in a 96-well round-bottom plate (serum + virus mix) at a total of 100 ul and incubated at 37°C/5% CO_2_. After 1 hour, media was removed from the Vero E6 cells and 100 μl of the incubated serum + virus mix was added to the appropriate wells in duplicate (nLuc) or quadruplicate for SARS-CoV-2 variants. Cells were incubated with serum + virus for 1 h with agitation every 10 min. SARS-CoV-2 variant infections were then incubated for 48 h at 37°C/5% CO_2_ prior to processing for qRT-PCR. Media: virus mixture was removed from SARS-CoV-2 nLuc infected cells and replaced with DMEM + 2% FBS then incubated for 24 h at 37°C/5% CO_2_ until luciferase readout. For readout of the SARS-CoV-2-nLuc neutralization assay, media was removed from cells and replaced with 50 μl PBS. An equal volume of 50 μl Nano Luciferase Assay Substrate (Promega) was added to each well and mixed before transfer to a black 96-well plate. Luciferase signal was measured using Synergy LX multi-mode reader (BioTek) following the manufacturer’s instructions. Neutralization was indicated by greater than 50% reduction in luciferase signal compared to serum from the same animals prior to vaccination (non-immune controls). For the neutralizing assays using SARS-CoV-2 wild-type and variants, qRT-PCR was performed on the viral RNA released into the supernatant from the cells exposed to the sera. All RNA extracts were collected using a QIAamp 96 Viral RNA Kit (Qiagen, MD, US) following the manufacturers protocol. The titer resulting in 50% neutralization was based on virus production measured by qRT-PCR. qRT-PCR reactions were performed on a QuantStudio 5 Real-Time PCR system (Applied Biosystems, Waltham, MA, US) with TaqMan Fast Virus 1-Step reagents (Applied Biosystems, Waltham, MA, US) following the manufacturer’s protocol.
(TIF)

S5 Table Cytokine/chemokine in sera pre-/post-immunization of macaques. We used a multiplex instrument MAGPIX to quantify the cytokine/chemokine profiles in the cell culture supernatant of VSV-SARS-CoV-2 infected human PBMCs **(A)** and the cytokine/chemokine profiles within the serum of VSV-SARS-CoV-2 vaccinated NHP **(B)**. Serum samples or cell culture supernatant samples were prepared following the manufacturer’s instructions (Milliplex MAP kit, Millipore) and 25 μl of undiluted samples was added to 25 μl of assay buffer. Then, 25 μl of magnetic beads coated with specific antibodies (HT17MG-14K-PX25 combined with HIL18MAG-66K, Milliplex MAP Kit, Millipore) was added to this solution and the reaction was incubated at 4°C for 16 h. Next, the beads were washed and incubated with 25 μl of biotinylated detection antibody at room temperature for 1 hour. To complete the reaction 25 μl of Streptavidin–Phycoerythrin conjugate compound was added and allowed to incubate at room temperature for 30 minutes. The beads were then washed and incubated with 50 μl of sheath fluid at room temperature for 5 minutes. The samples were analyzed on MAGPIX instruments. The concentration of the analytes was then determined by MAGPIX xPONENT software. The assays were run in duplicate to confirm the results. Analytes were normalized to total protein concentration. Twenty six analytes were studied: interleukin 1 beta (IL-1β), interleukin-2 (IL-2), interleukin-4 (IL-4), interleukin-5 (IL-5), interleukin-6 (IL-6), interleukin-9 (IL-9), interleukin-10 (IL-10), interleukin-12p70 (IL-12p70), interleukin-13 (IL-13), interleukin-15 (IL-15), interleukin-17A (IL-17A), interleukin-17E/ interleukin-25 (IL-17E/25), interleukin-17F (IL-17F), interleukin-18 (IL-18), interleukin-21 (IL-21), interleukin-22 (IL-22), interleukin-23 (IL-23), interleukin-27 (IL-27), interleukin-28A (IL-28A), interleukin-31 (IL-31), interleukin-33 (IL-33), granulocyte-macrophage colony-stimulating factor (GM-CSF), interferon gamma (IFNγ), macrophage inflammatory protein 3 alpha (MIP-3α), tumor necrosis factor alpha (TNFα), and tumor necrosis factor beta (TNF β).
(TIF)

S6 Table The vaccination of hACE2 transgenic mice with rVSV-SARS-CoV-2-msp-S_F_-Gtc and challenge with SARS-CoV-2. Six-week-old female hACE2 transgenic mice (n = 5 per group) were prime-immunized with rVSV_Ind_-msp-S_F_-Gtc and boost-immunized with rVSV_Ind_-msp-S_F_-Gtc or rVSV_NJ_-msp-S_F_-Gtc two weeks after prime-immunization. Four weeks after boost-immunization, mice were challenged intranasally with 1x10^5^ PFU of SARS-CoV-2 (S clade, National Culture Collection for Pathogens (NCCP) #43326 Korea Disease Control and Prevention Agency) in a 50 μl volume intranasally under anesthesia. The survival and body weight of each mouse was monitored daily.
(TIF)

S7 Table Histopathological findings of the lungs on day 3 after challenge. Lesions in the lung tissues were graded semi-quantitatively depending on their severity. SARS-CoV-2 infection-related inflammatory lesions included inflammatory foci, characterized by infiltration of inflammatory cells around blood vessels and interstitial alveolar inflammation. In the cases of inflammatory foci, we graded inflammation based on the following criteria: 1) cases with very weak cell infiltration, close to normal, but there is recognizable inflammatory cell infiltration around some vessels (inflammatory score = 0.5), 2) some blood vessels with mild perivascular inflammatory cell infiltration in the lung section (minimal, inflammatory score = 1), 3) some blood vessels in the lung section with prominent infiltration of inflammatory cells (mild, inflammatory score = 2), 4) more than 50% of blood vessels in the lung section have prominent perivascular infiltration of inflammatory cells (moderate, inflammatory score = 3), 5) most blood vessels in the lung section have prominent and heavy perivascular infiltration of inflammatory cells (severe, Inflammatory score = 4). We scored the level of inflammation in each case by combining the two different inflammation scores of inflammatory foci and alveolitis. We then calculated the mean inflammatory score of each group from the individual inflammatory scores.
(TIF)

S8 Table Histopathological findings of the lungs on day 7 post challenge. Lesions in the lung tissues were graded semi-quantitatively depending on their severity. SARS-CoV-2 infection-related inflammatory lesions included inflammatory foci, characterized by infiltration of inflammatory cells around blood vessels and interstitial alveolar inflammation. In the cases of inflammatory foci, we graded inflammation based on the following criteria: 1) cases with very weak cell infiltration, close to normal, but there is recognizable inflammatory cell infiltration around some vessels (inflammatory score = 0.5), 2) some blood vessels with mild perivascular inflammatory cell infiltration in the lung section (minimal, inflammatory score = 1), 3) some blood vessels in the lung section with prominent infiltration of inflammatory cells (mild, inflammatory score = 2), 4) more than 50% of blood vessels in the lung section have prominent perivascular infiltration of inflammatory cells (moderate, inflammatory score = 3), 5) most blood vessels in the lung section have prominent and heavy perivascular infiltration of inflammatory cells (severe, Inflammatory score = 4). We scored the level of inflammation in each case by combining the two different inflammation scores of inflammatory foci and alveolitis. We then calculated the mean inflammatory score of each group from the individual inflammatory scores.
(TIF)

S1 Data Excel spreadsheet containing, in separate sheets, the underlying numerical data for figure panels 5B, 5C, 6A, 6B, 7, 8, 9A, 9B, 9C, 10, S5, S6A, S6B, S8A, S8B, S8C, S8D, and S10. (XLSX)
