# Correction: A vesicular stomatitis virus-based prime-boost vaccination strategy induces potent and protective neutralizing antibodies against SARS-CoV-2

**DOI:** 10.1371/journal.ppat.1011000

**Published:** 2022-11-29

**Authors:** Gyoung Nyoun Kim, Jung-ah Choi, Kunyu Wu, Nasrin Saeedian, Eunji Yang, Hayan Park, Sun-Je Woo, Gippeum Lim, Seong-Gyu Kim, Su-Kyeong Eo, Hoe Won Jeong, Taewoo Kim, Jae-Hyung Chang, Sang Hwan Seo, Na Hyung Kim, Eunsil Choi, Seungho Choo, Sangkyun Lee, Andrew Winterborn, Yue Li, Kate Parham, Justin M. Donovan, Brock Fenton, Jimmy D. Dikeakos, Gregory A. Dekaban, S. M. Mansour Haeryfar, Ryan M. Troyer, Eric J. Arts, Stephen D. Barr, Manki Song, C. Yong Kang

In the Construction of rVSV-SARS-CoV-2 vaccines subsection of the Materials and methods, there is an error in the first sentence of the first paragraph. The GenBank accession number is incorrect. The correction accession number is GenBank: MN908947.3.
