# Supplementary figures and images for: A vesicular stomatitis virus-based prime-boost vaccination strategy induces potent and protective neutralizing antibodies against SARS-CoV-2
Source: PLoS Pathog. 2021 Dec 16;17(12):e1010092. doi: 10.1371/journal.ppat.1010092 (PMC8675757; doi:10.1371/journal.ppat.1010092)

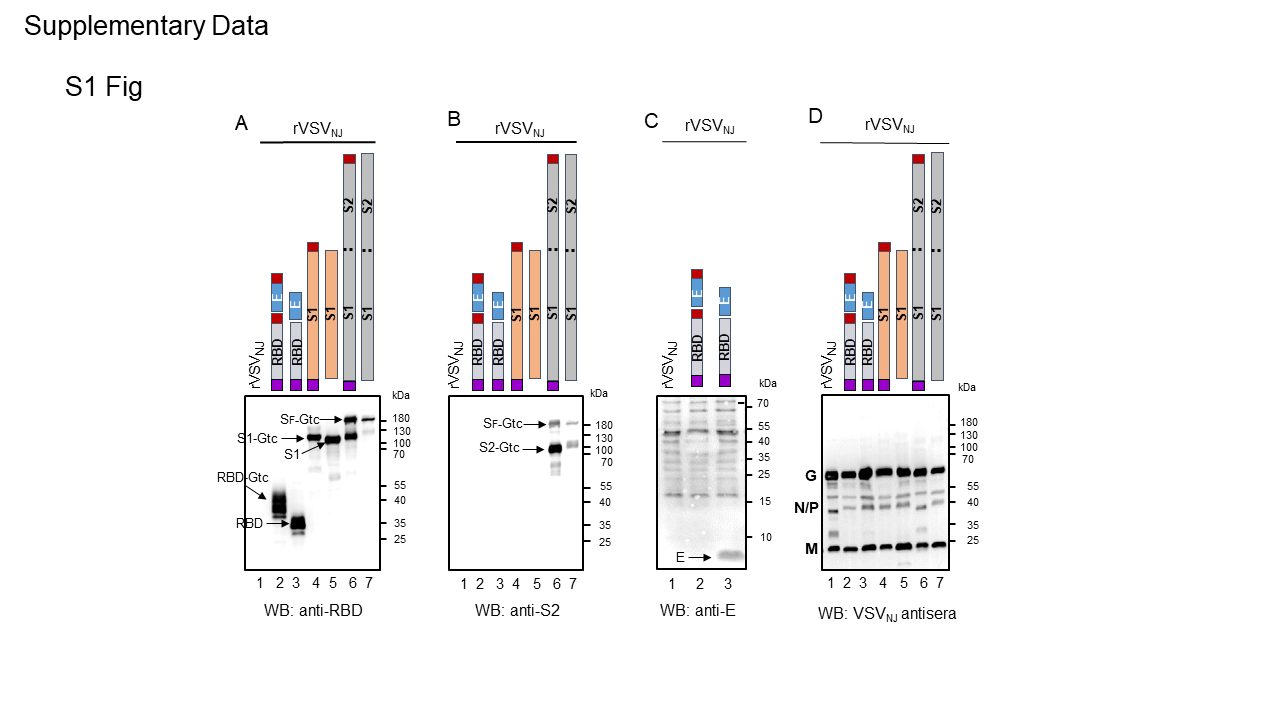

Supplement: S1 Fig — To check the expression of SARS-CoV-2 RBD, S1, and SF from rVSVNJ-SARS-CoV-2 infected cells, BHK-21 cells were infected with the virus at an MOI of 6. After six hours incubation at 37°C, cell lysates were prepared and protein expression was determined by Western blot. Cell lysates were loaded in 5 μg quantity for SDS-PAGE. RBD, S1, and SF proteins were detected by rabbit antibody against SARS-CoV-2 RBD. S2 protein was detected by rabbit antibody against SARS-CoV-2 S2. E protein was detected by rabbit antibody against SARS-CoV-2 E peptides. (A) Expression of RBD, S1, and SF with and without msp and Gtc. (B) Expression of S2 with and without Gtc. (C) Expression of E protein. (D) Expression of VSVNJ N, P, M, and G proteins. Purple box: honeybee msp, red box: VSV Gtc. (TIF) [file ppat.1010092.s001.tif]

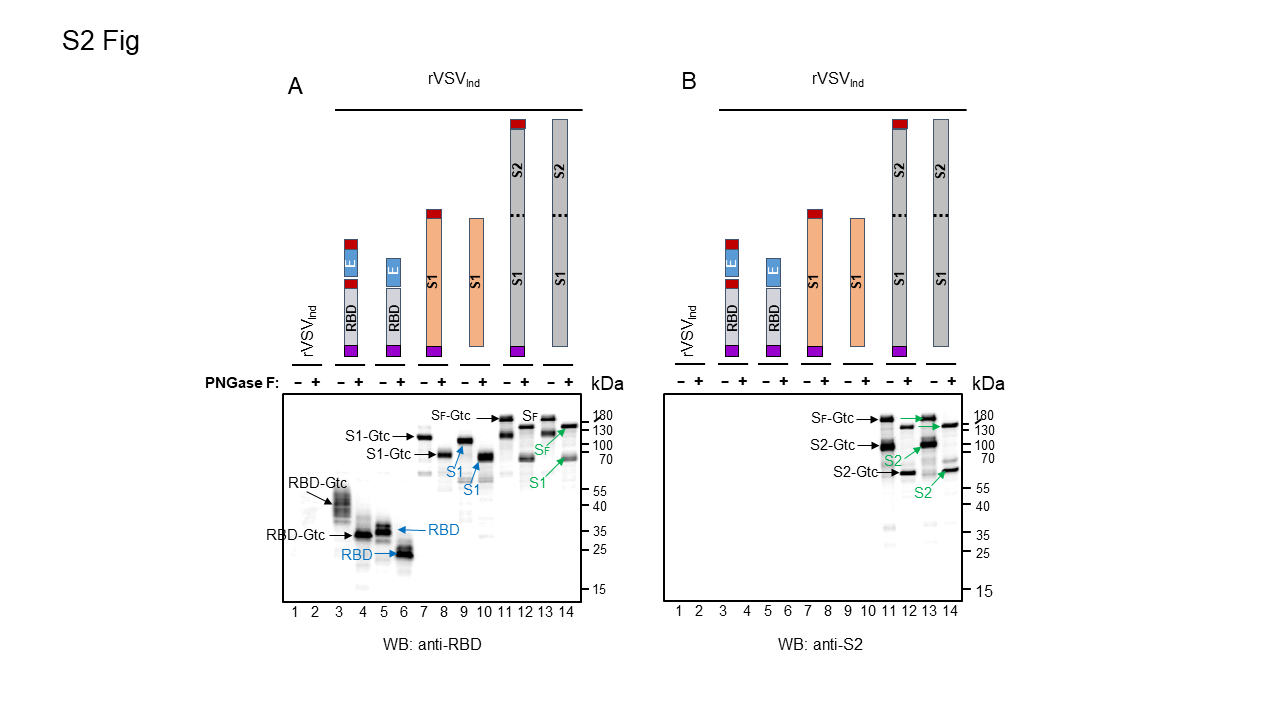

Supplement: S2 Fig — To assess protein glycosylation, 20 μg of infected cell lysates from rVSVInd(GML) infection (Fig 2) were treated with 10 units of Peptide N-Glycosidase F (PNGase F, Sigma-Aldrich, G5166) and incubated at 37°C for 3 hrs. according to the manufacture’s protocol. The migratory pattern of the proteins was examined by Western blot analysis. Five μg of the PNGase F treated and untreated cell lysates were loaded on the SDS-PAGE. RBD, S1, and SF were detected by an antibody against SARS-CoV-2 RBD and S2 was detected by an antibody against SARS-CoV-2 S2. (A) Detection of RBD, S1, and SF proteins with and without Gtc in the PNGase F untreated (-) and treated (+) cell lysates. (B) Detection of S2 and SF proteins with and without Gtc in the PNGase F untreated (-) and treated (+) cell lysates. Purple box: honeybee msp, red box: VSV Gtc. (TIF) [file ppat.1010092.s002.tif]

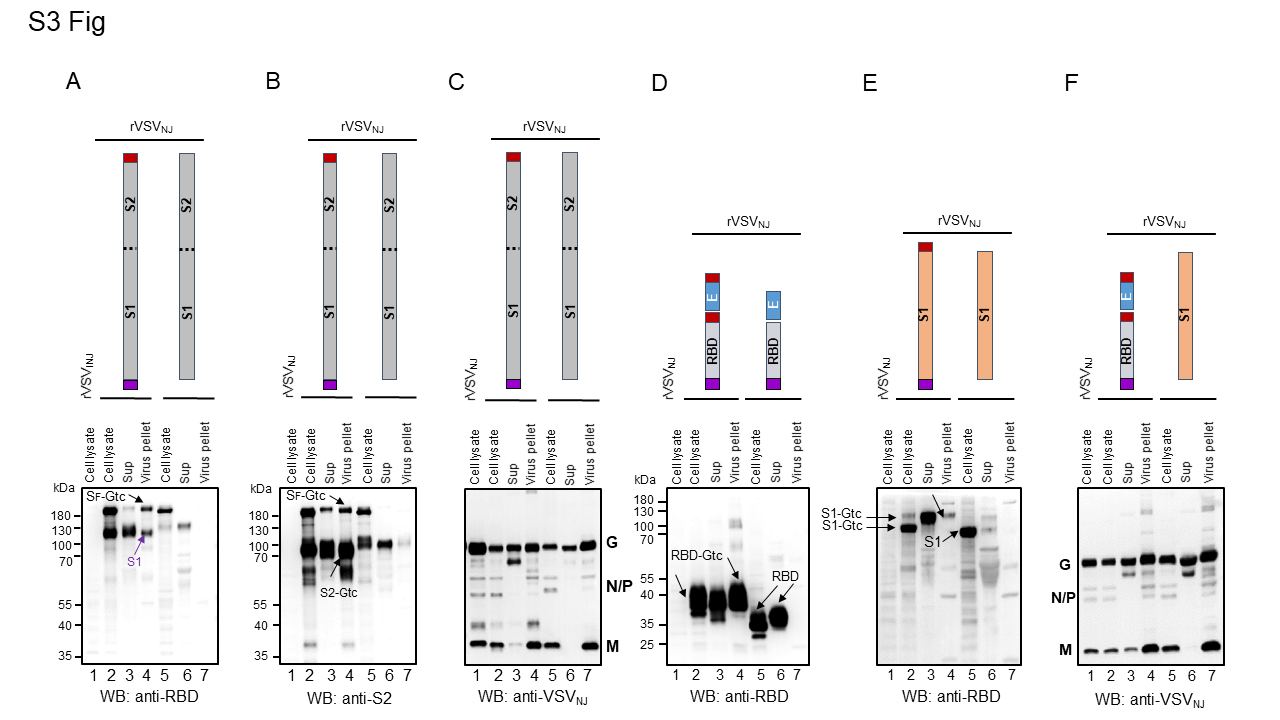

Supplement: S3 Fig — Incorporation of SARS-CoV-2 S1, S2, SF, and RBD with or without VSV Gtc into rVSVNJ particles was examined by infecting BHK-21 cells with rVSVNJ-SARS-CoV-2 at an MOI of 3. The rVSVNJ-SARS-CoV-2 infected cells were incubated at 31°C for 6 hrs. Infected cell lysates were prepared in lysis buffer (lanes 1, 2, and 5). Culture media from the infected cells was centrifuged at 500 x g for 10 minutes and supernatant was filtered through a 0.45 μm filter to remove cell debris. The filtered culture media was loaded onto 1 ml of 25% sucrose cushion and ultra-centrifuged at 150,900 x g for 3 hrs. Supernatant on top of the 25% sucrose cushion was collected to check the soluble proteins in the media (lanes 3 and 6). Pelleted samples were checked for proteins incorporated into VSV particles (lanes 4 and 7). We detected RBD, S1, and SF proteins by Western blot using an antibody against SARS-CoV-2 RBD protein. S2 and SF proteins were detected by the rabbit antibody against SARS-CoV-2 S2. (A) Detection of SF and S1 proteins in cell lysate, concentrated culture media, and virus pellet from cells infected with rVSVNJ-msp-SF-Gtc or rVSVNJ-SF. (B) Detection of SF and S2 proteins in cell lysate, concentrated culture media, and virus pellet from cells infected with rVSVNJ-msp-SF-Gtc or rVSVNJ-SF. (C) Detection of VSVNJ proteins in cell lysate, concentrated culture media, and virus pellet from cells infected with rVSVNJ-msp-SF-Gtc or rVSVNJ-SF. (D) Detection of S1 protein in cell lysate, concentrated culture media, and virus pellet from cells infected with rVSVNJ-msp-S1-Gtc or rVSVNJ-S1. (E) Detection of RBD proteins in cell lysate, concentrated culture media, and virus pellet from cells infected with rVSVNJ-msp-RBD-Gtc+E-Gtc or rVSVNJ-msp-RBD+E. (F) Detection of VSVNJ proteins in cell lysate, concentrated culture media, and virus pellet from the cells infected with rVSVNJ-msp-RBD-Gtc+E-Gtc or rVSVNJ-msp-RBD+E. Purple box: honeybee msp, red box: VSV Gtc. (TIF) [file ppat.1010092.s003.tif]

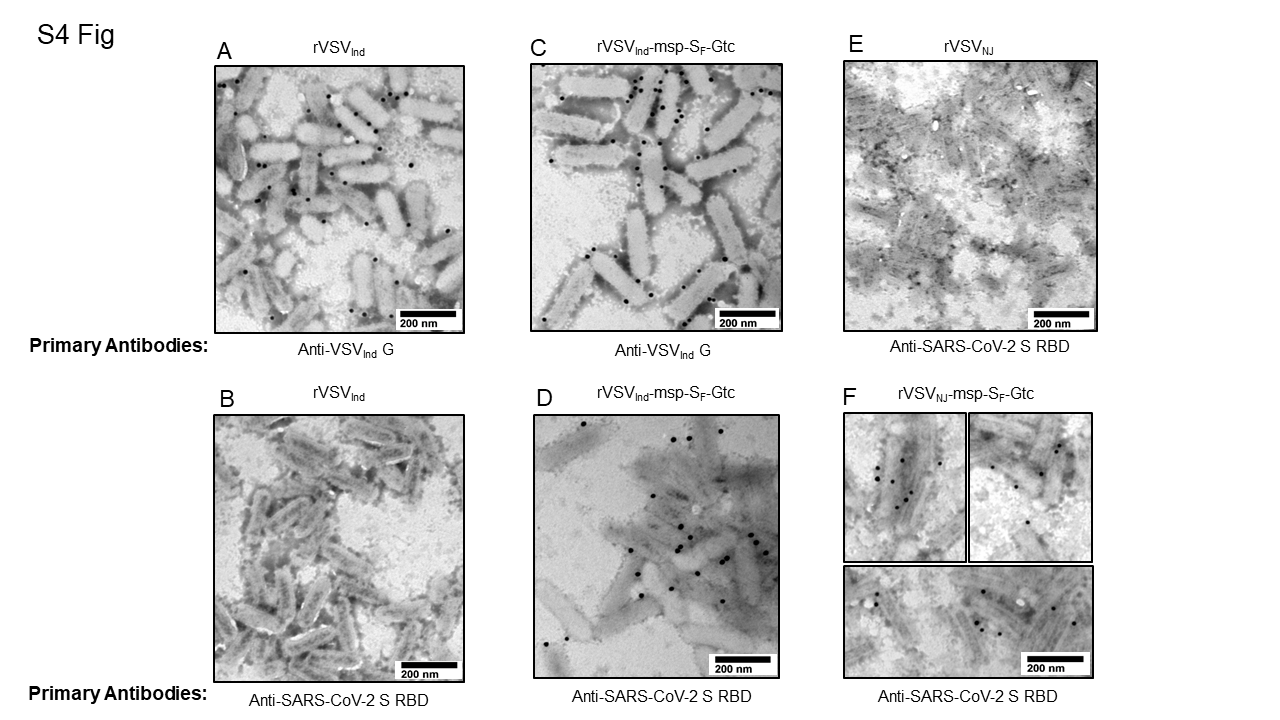

Supplement: S4 Fig — The recombinant VSVs, rVSVInd, rVSVInd-msp-SF-Gtc, rVSVNJ, and rVSVNJ-msp-SF-Gtc were prepared as described in materials and methods. Ten μl of virus samples were applied onto the Nickel grids (FCF 400-Ni-TC, Electron Microscopy Sciences). The samples were treated with 10 μl of blocking solution (PBS w/ 2% BSA-C, Electron Microscopy Sciences) for 15 minutes. The grids were washed on drops of incubation solution (PBS w/ 0.1% BSA-C) for 2 x 5 minutes. The samples were treated with 10 μl of tenfold diluted primary antibodies, which were diluted with PBS w/ 0.1% BSA-C. Mouse anti-VSVInd G was used for VSV G protein. Rabbit anti-SARS-CoV-2 S was used for SARS-CoV-2 S. The samples were treated with an appropriate gold conjugate reagent for 2 hrs. The gold conjugate reagent was diluted tenfold in the incubation solution. Goat-anti-mouse IgG-gold was used for anti-VSV G treated samples. Goat-anti-rabbit IgG-gold was used for anti-SARS-CoV-2 S treated samples. The samples were stained with 0.5% phosphotungstic acid for 30 seconds. The samples were examined with the transmission electron microscope and an imaging system (Philips CM10). (A) rVSVInd labeled with an antibody against VSVInd glycoprotein antibody. (B) rVSVInd labeled with an antibody against RBD of SARS-CoV-2 spike protein. (C) rVSVInd-msp-SF-Gtc labeled with an antibody against VSVInd glycoprotein antibody. (D) rVSVInd-msp-SF-Gtc labeled with an antibody against RBD of SARS-CoV-2 spike protein. (E) rVSVNJ labeled with an antibody against RBD of SARS-CoV-2 spike protein. (F) rVSVNJ-msp-SF-Gtc labeled with an antibody against RBD of SARS-CoV-2 spike protein. (TIF) [file ppat.1010092.s004.tif]

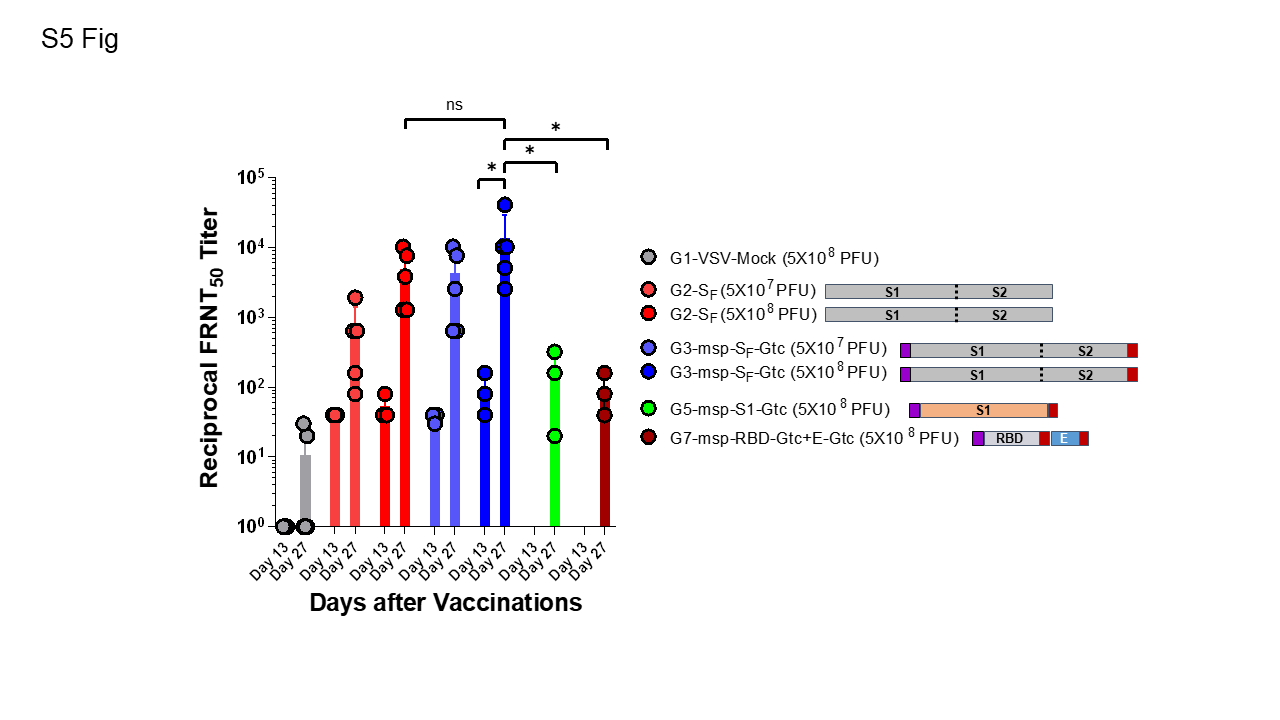

Supplement: S5 Fig — Mice were immunized and sera were collected as described in Fig 5. SARS-CoV-2 neutralization was determined by FRNT50 assay as has been described in Fig 6. Statistical significance was determined by two-way ANOVA with Tukey’s correction (*, p < 0.05; ns, not significant). The data were presented as means with error bars of standard deviation. Purple box: honeybee msp, red box: VSV Gtc. (TIF) [file ppat.1010092.s005.tif]

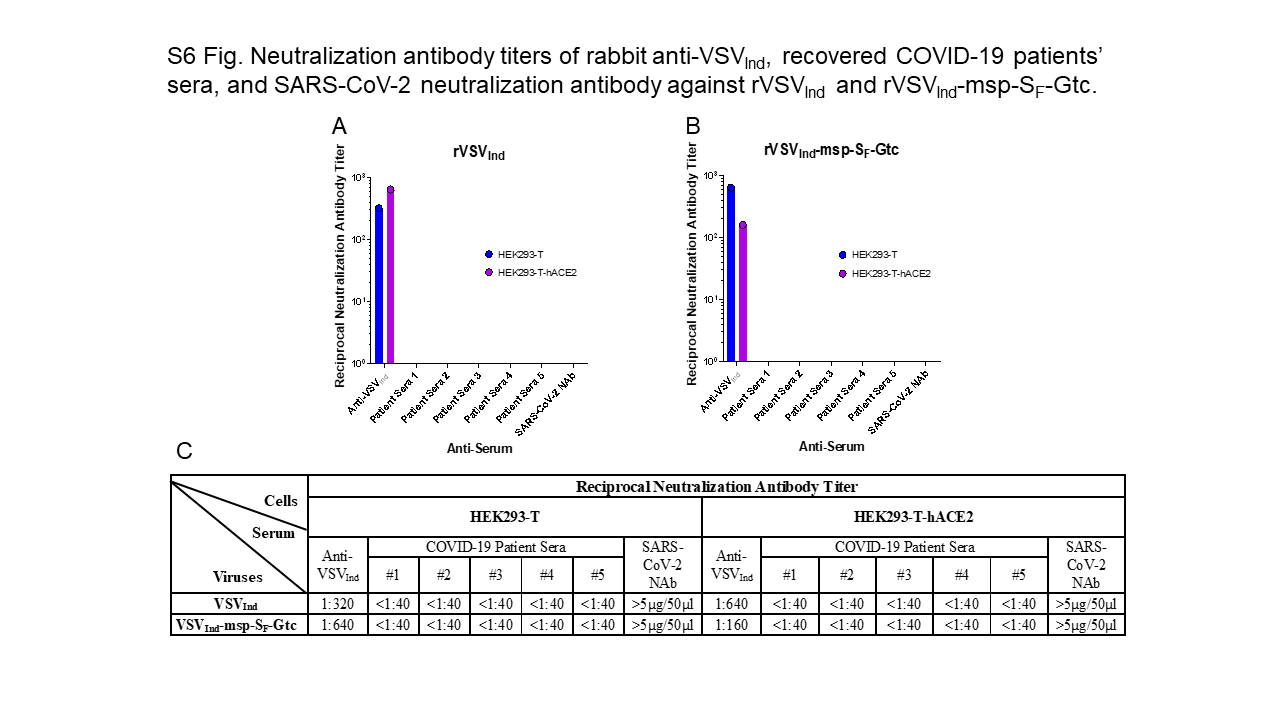

Supplement: S6 Fig — We diluted rabbit serum against VSVInd and recovered COVID-19 patients’ sera twofold serially starting at 1: 40 dilutions to 1: 40,960 dilutions. The reciprocal titer of all five COVID-19 patients’ sera for the 50% neutralization of SARS-CoV-2-Wuhan strain was >1/640. SARS-CoV-2 neutralizing antibody (MBS9141964, MyBioSource.com), which 50% inhibitory concentration is 0.496μg/mL, was used as a positive neutralization antibody against SARS-CoV-2. SARS-CoV-2 neutralizing antibody (MBS9141964, MyBioSource.com) was diluted two-fold serially starting at 100 μg/ml concentrations to 0.1 μg/ml concentrations. rVSVInd and rVSVInd-msp-SF-Gtc were diluted to 2X104 PFU/ml, and 80 μl of the diluted VSV and 80 μl of the diluted antisera were mixed and incubated at 37°C for 1hr. Fifty μl of the VSV and antisera mixture was added to HEK293-T or HEK293-T-hACE2 cells in 96 well cell-culture plates for the adsorption of 1 hr. We added 150 μl of complete DMEM to the cells and incubated for three days to determine the antibody titer to neutralize 100% of the rVSVInd and rVSVInd-msp-SF-Gtc in HEK293-T or HEK293-T-hACE2 cells. (A) Neutralization antibody titers of rabbit anti-VSVInd, recovered COVID-19 patients’ sera, and SARS-CoV-2 neutralization antibody against rVSVInd. (B) Neutralization antibody titer of rabbit anti-VSVInd, recovered COVID-19 patients’ sera, and SARS-CoV-2 neutralization antibody against rVSVInd-msp-SF-Gtc. (C) Reciprocal neutralizing antibody titers. (TIF) [file ppat.1010092.s006.tif]

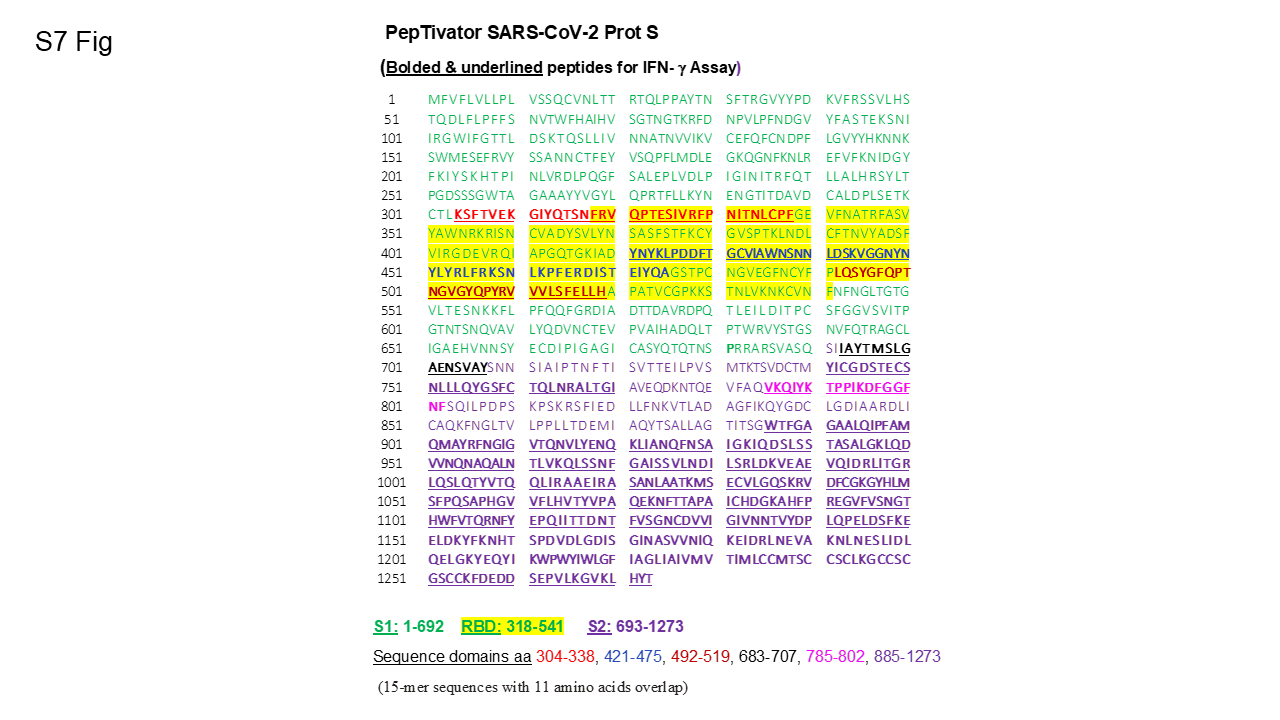

Supplement: S7 Fig — A pool of peptides (in bold and underlined) were used to stimulate T cells. (TIF) [file ppat.1010092.s007.tif]

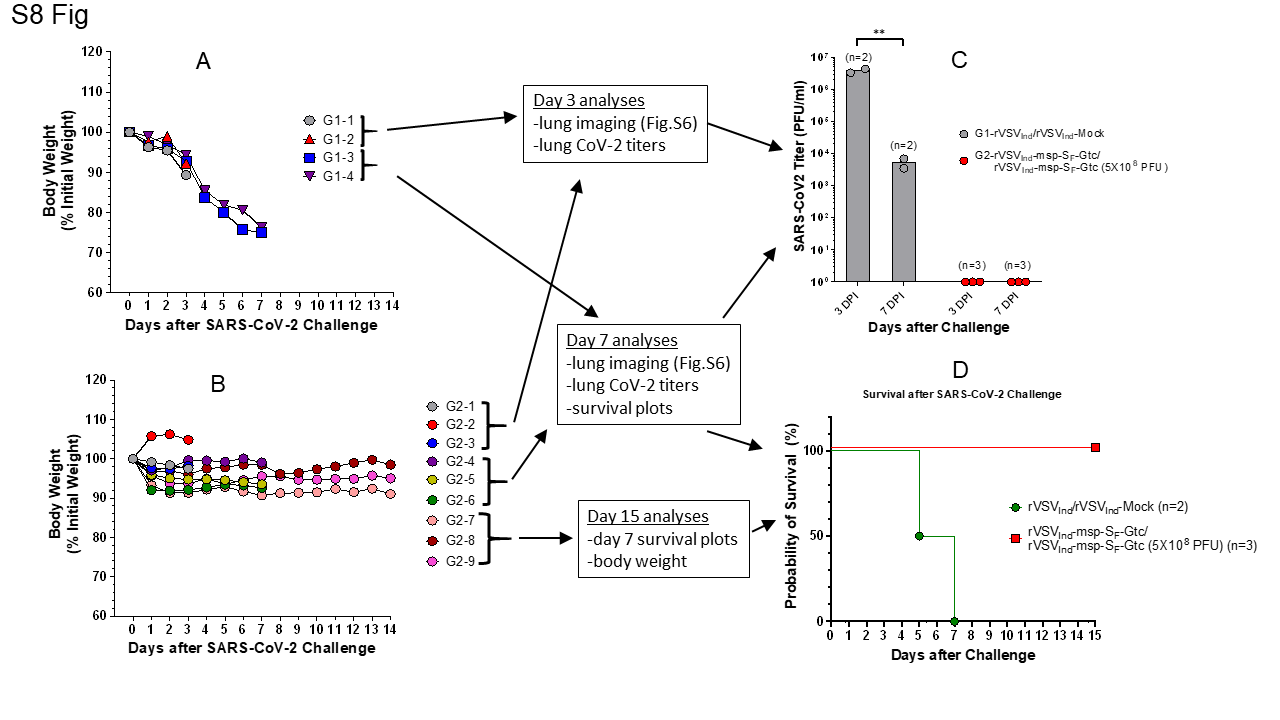

Supplement: S8 Fig — Six-week-old female hACE2 transgenic mice were prime-vaccinated with rVSVInd-msp-SF-Gtc (n = 9) or rVSVInd (n = 4) and boost vaccinated with rVSVInd-msp-SF-Gtc or rVSVInd two weeks after prime-vaccination. Four weeks after boost-vaccination (S6 Table), mice were challenged intranasally with 5X105 PFU SARS-CoV-2. Body weight and survival of each mouse were monitored daily. (A) Individual body weight for mice vaccinated with rVSVInd-Mock and challenged with SARS-CoV-2. (B) Individual body weight for mice vaccinated with rVSVInd-msp-SF-Gtc and challenged with SARS-CoV-2. (C) SARS-CoV-2 viral loads in the lungs of vaccinated and challenged hACE2 transgenic mice. Right lobes of mice lungs were aseptically removed from the mice on day 3 and day 7 after the SARS-CoV-2 challenge. Infectious SARS-CoV-2 was quantified by plaque assay on Vero E6 cells. Statistical significance was determined by two-way ANOVA with Tukey’s correction (**, p< 0.005). (D) Mouse survival after the SARS-CoV-2 challenge. (TIF) [file ppat.1010092.s008.tif]

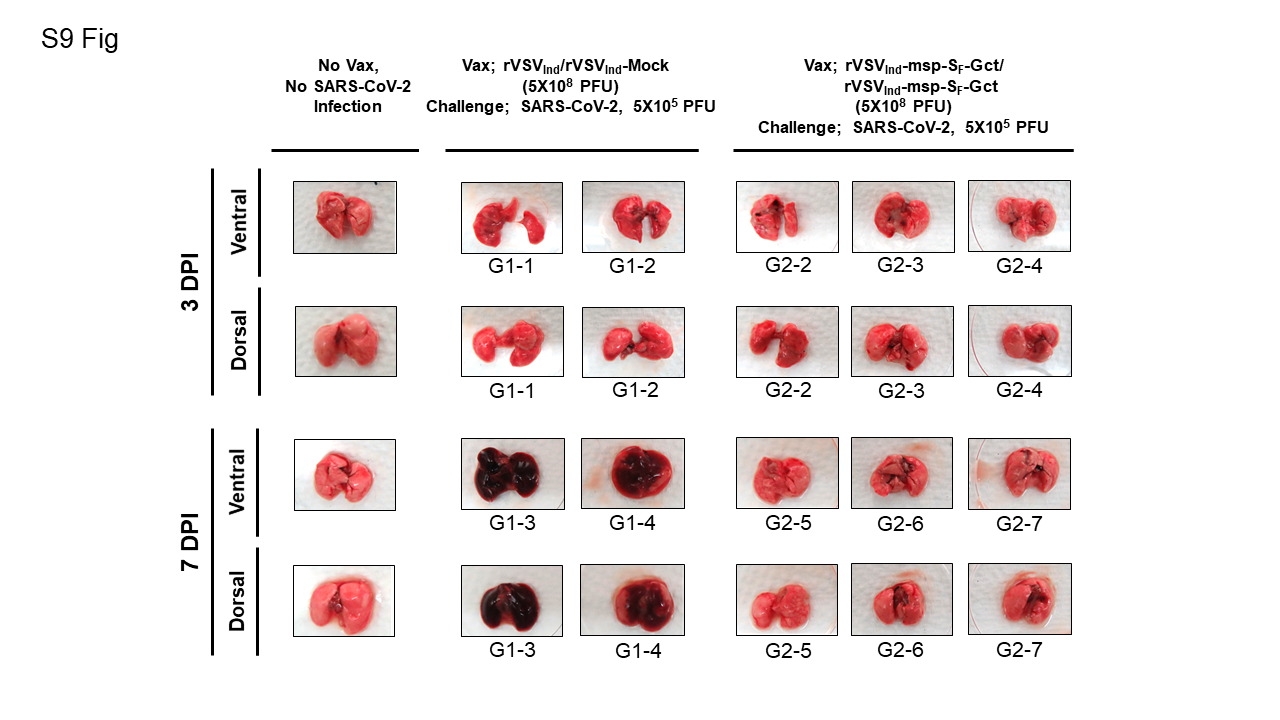

Supplement: S9 Fig — Six-week-old female hACE2 transgenic mice were prime-vaccinated with rVSVInd-msp-SF-Gtc (n = 9) or rVSVInd (n = 4) and boost vaccinated with rVSVInd-msp-SF-Gtc or rVSVInd two weeks after prime-vaccination. Four weeks after boost-vaccination (S6 Table), mice were challenged intranasally with 5X105 PFU of SARS-CoV-2. Two mice from the rVSVInd-Mock vaccinated groups and three mice from rVSVInd-msp-SF-Gtc vaccinated group were euthanized on day 3 and on day 7 after SARS-CoV-2 challenge to check the virus loads in the lung. Before isolation of the SARS-CoV-2 from the infected lungs, pictures were taken. We took pictures of the ventral and dorsal sides of each lung. (TIF) [file ppat.1010092.s009.tif]

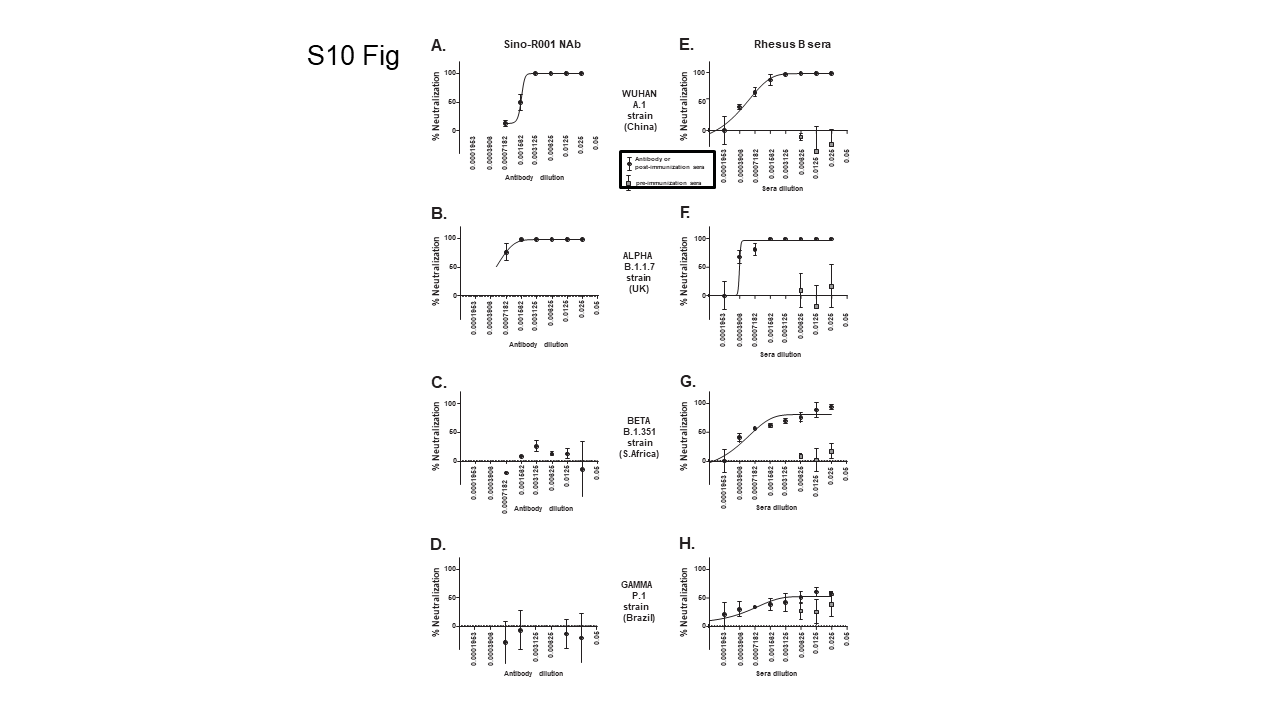

Supplement: S10 Fig — For measuring neutralization by the monoclonal Sino-R001 NAb and in sera of macaques immunized with prime-boost with rVSV-msp-SARS-CoV-2-Gtc, monoclonal Nab and sera from macaque B was serially diluted two-fold starting at 1:40 and added to Vero E6 cells along with 100 plaque-forming units (PFU) of SARS-CoV-2 wild type (panel A for NAb and panel E for Rhesus macaque B), SARS-CoV-2 Alpha variant (panel B and F), SARS-CoV-2 Beta variant (panel C and G), or SARS-CoV-2 Gamma variant (panel D and H). The monoclonal Sino-R001 NAb was only diluted to 1/640. All assays were performed in quadruplicate. Virus production was measured by qRT-PCR as described in the materials and methods, converted to % neutralization based on maximal replication in absence of sera. In panels E through H, the level of neutralization of heat-inactivated preimmunization sera from macaque B was also run and shown on the graph for only the 1/40, 1/80, and 1/160 dilutions. (TIF) [file ppat.1010092.s010.tif]

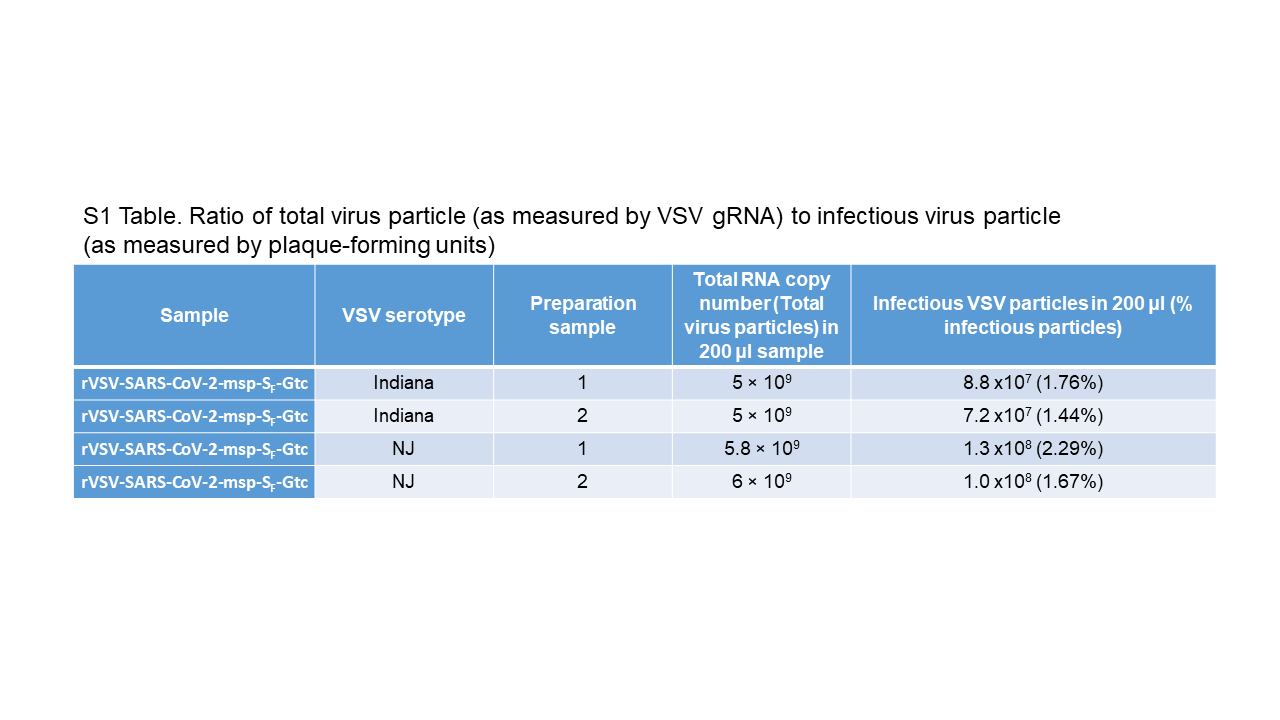

Supplement: S1 Table — The purified VSV-SARS-CoV-2 were diluted to 5X107 PFU in 200 μl volume. Fifty μl of the diluted viruses was titrated for the infectious virus particles by plaque assay, and 140 μl of the virus was used for genomic RNA isolation. VSV genomic RNA was isolated by using QIAamp Viral RNA kit (Qiagen, Cat. No. 52904). The extracted RNA was resuspended in 60 μl elution buffer. Ten μl of the isolated RNA was used for reverse transcription by SuperScript IV Reverse Transcriptase kit (Invitrogen, Cat. No. 18090200) and yield 20 μl cDNA product. Two μl of the cDNA was used as templates for the qPCR. The SARS-CoV-2 S gene specific qPCR primers and a probe were as follow: Forward primer: 5´-GCCCAGGTGAAGCAAATCTA-3´; Revers primer: 5´-GAACAGCAGGTCCTCGATAAAG-3´; Probe: 5´-/56-FAM/CTGCCTGATCCATCCAAG CCTTCT/3IABkFQ-3´. The qPCR was carried out with QuantStudio 5 real time PCR system (Applied biosystems). The DNA plasmid, pVSVInd-COV-2 msg-SF-Gtc was used as a copy number standard and the five different concentrations of the plasmid for the standard curve were 5×10^7^ copies, 5×10^6^ copies, 5×10^5^ copies, 5×10^4^ copies, 5×10^3^ copies. (TIF) [file ppat.1010092.s011.tif]

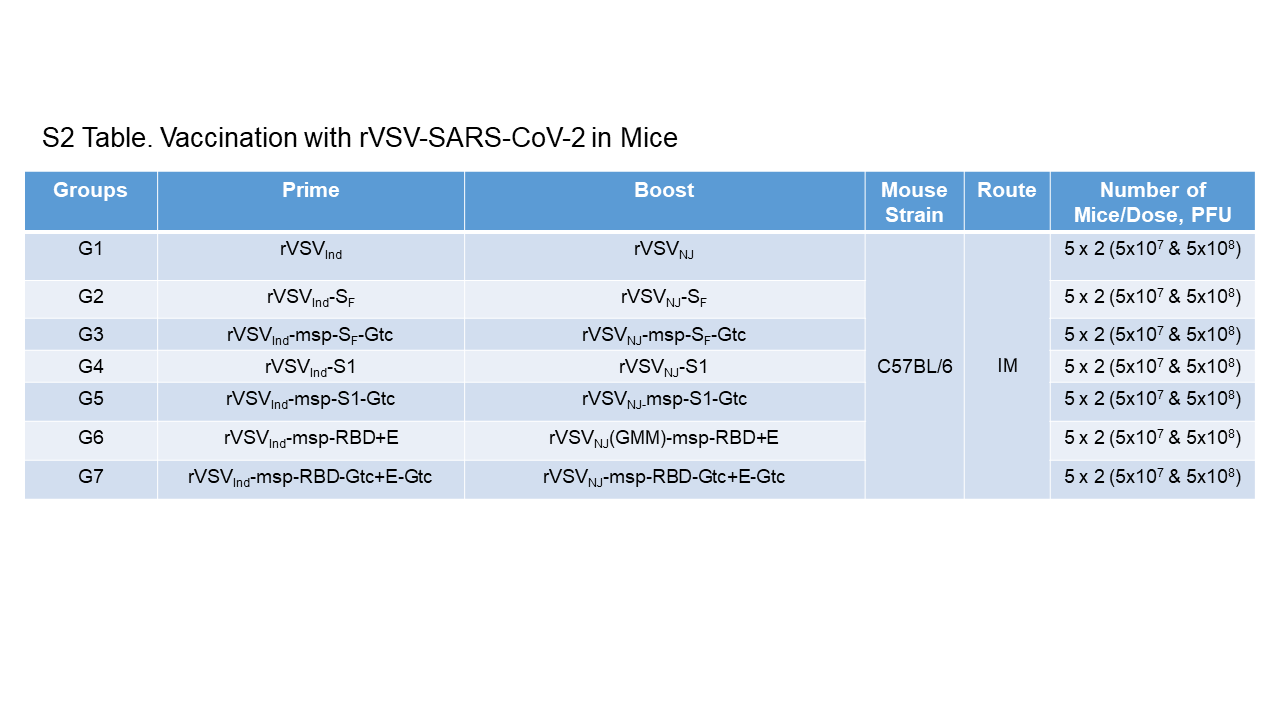

Supplement: S2 Table — To analyze humoral immune responses towards SARS-CoV-2 SF, S1 and RBD, we vaccinated C57BL/6 mice (n = 5/vaccination group) intramuscularly with rVSV vaccine vectors at 5x10^7^ PFU/mouse or 5x10^8^ PFU/mouse. We prime vaccinated each mouse with rVSVInd constructs. Two weeks after prime immunization, we boost-immunized the mice with rVSVNJ constructs. (TIF) [file ppat.1010092.s012.tif]

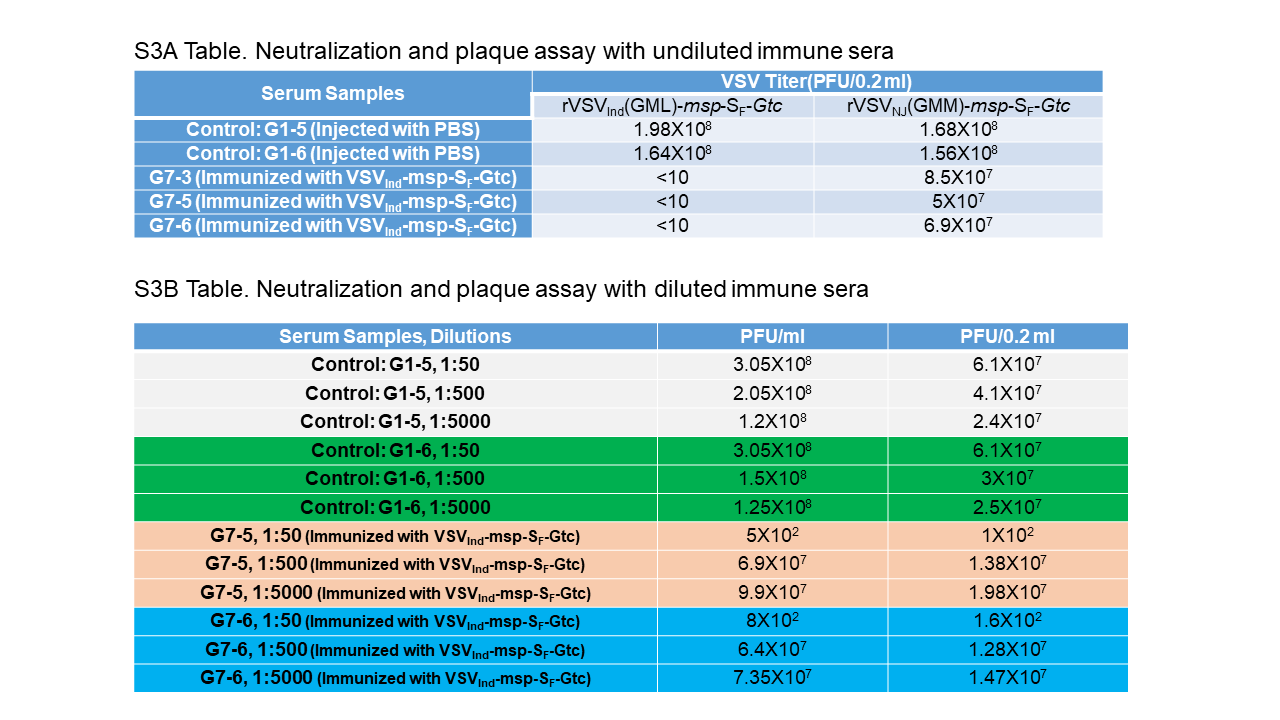

Supplement: S3 Table — Mouse immune sera against VSV-SARS-CoV-2 msp-SF-Gtc were prepared by vaccinating interferon α and β receptor knock out mice (Ifnar-/- mice, Jackson Laboratory, B6.129S2-Ifnar1tm1Agt/Mmjax) once with 5X108 PFU of rVSVInd-msp-SF-Gtc. The immune serum was collected at 28 days after the vaccination. For neutralization, 5X107 PFU of rVSVInd-msp-SF-Gtc or rVSVNJ-msp-SF-Gtc in 100 μl were mixed with 100 μl of serum from PBS injected mice (n = 2) or with 100 μl of serum from VSV-SARS-CoV-2 msp-SF-Gtc vaccinated mice (n = 3). The virus and serum mixture was incubated for one hour at 37°C. The titer of viruses in the mixture was determined by plaque assay. (A) Neutralization of VSV-SARS-CoV-2-msp-SF-Gtc by undiluted immune sera. (B) Neutralization of VSV-SARS-CoV-2-msp-SF-Gtc by diluted immune sera. (TIF) [file ppat.1010092.s013.tif]

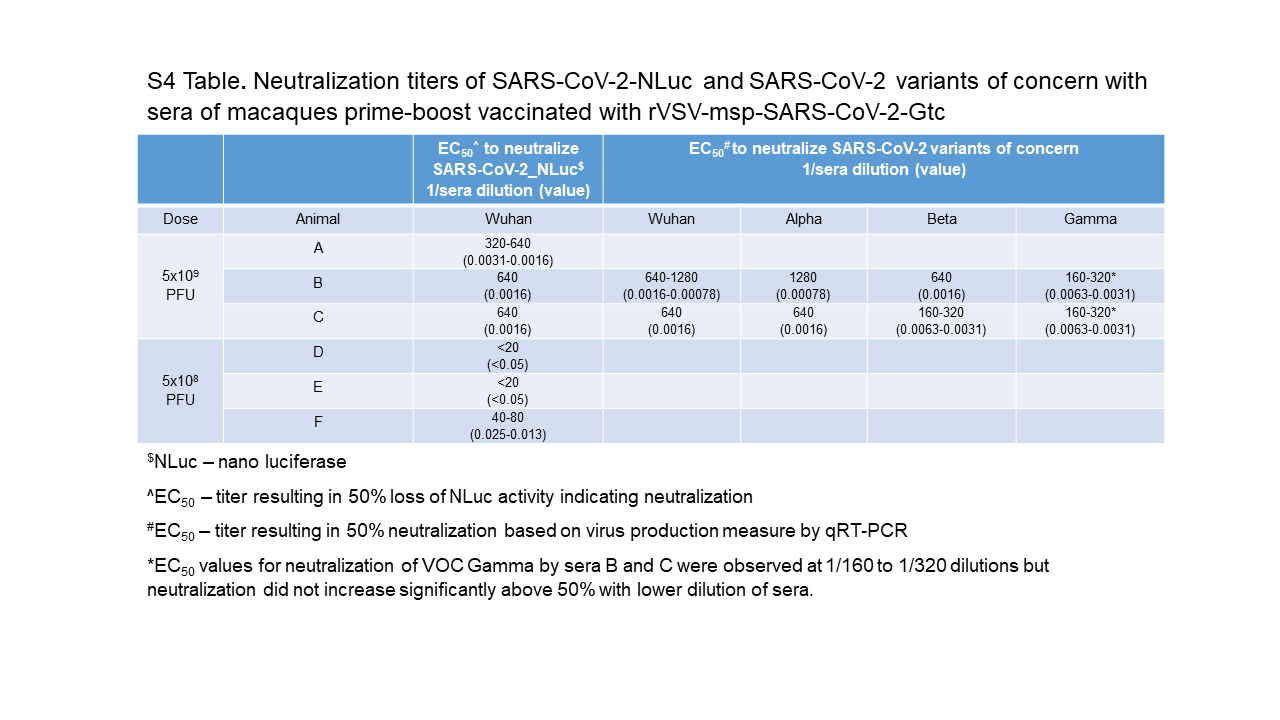

Supplement: S4 Table — Three macaques were prime-immunized intramuscularly with 109 PFU rVSVInd-SARS-CoV-2-msp-SF-Gtc and boost-immunized with 109 PFU of rVSVInd-SARS-CoV-2-msp-SF-Gtc 20 days after the prime-immunization. For measuring NAb in the sera of NHP, Vero E6 cells were seeded at 15,000 cells/well in a 96-well plate. At 48 hours post-seeding, the sera were serially diluted two-fold starting at 1:20. Diluted sera were incubated with 60 PFU SARS-CoV-2-Nano-Luciferase (nLuc) [58]; or 100 PFU of SARS-CoV-2 wild-type, SARS-CoV-2 Alpha variant, SARS-CoV-2 Beta variant, or SARS-CoV-2 Gamma variant in a 96-well round-bottom plate (serum + virus mix) at a total of 100 ul and incubated at 37°C/5% CO2. After 1 hour, media was removed from the Vero E6 cells and 100 μl of the incubated serum + virus mix was added to the appropriate wells in duplicate (nLuc) or quadruplicate for SARS-CoV-2 variants. Cells were incubated with serum + virus for 1 h with agitation every 10 min. SARS-CoV-2 variant infections were then incubated for 48 h at 37°C/5% CO2 prior to processing for qRT-PCR. Media: virus mixture was removed from SARS-CoV-2 nLuc infected cells and replaced with DMEM + 2% FBS then incubated for 24 h at 37°C/5% CO2 until luciferase readout. For readout of the SARS-CoV-2-nLuc neutralization assay, media was removed from cells and replaced with 50 μl PBS. An equal volume of 50 μl Nano Luciferase Assay Substrate (Promega) was added to each well and mixed before transfer to a black 96-well plate. Luciferase signal was measured using Synergy LX multi-mode reader (BioTek) following the manufacturer’s instructions. Neutralization was indicated by greater than 50% reduction in luciferase signal compared to serum from the same animals prior to vaccination (non-immune controls). For the neutralizing assays using SARS-CoV-2 wild-type and variants, qRT-PCR was performed on the viral RNA released into the supernatant from the cells exposed to the sera. All RNA extracts were collected using a QIAamp 96 Vir [file ppat.1010092.s014.tif]

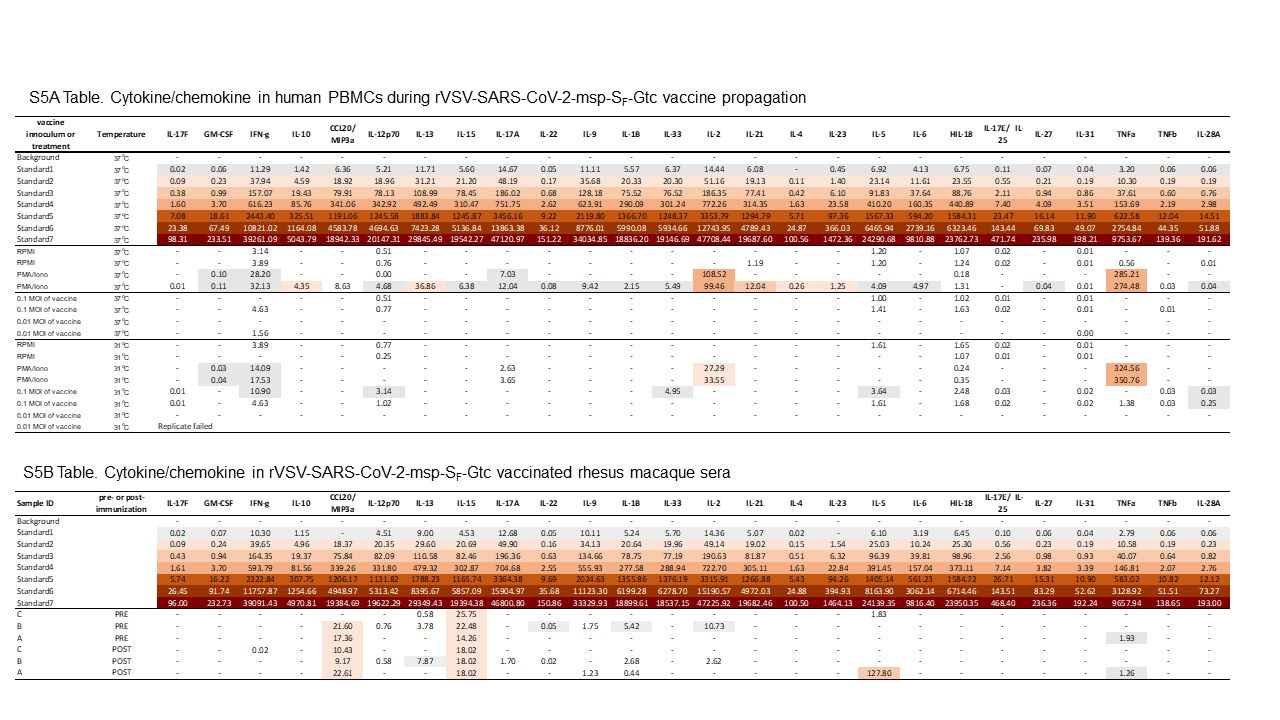

Supplement: S5 Table — We used a multiplex instrument MAGPIX to quantify the cytokine/chemokine profiles in the cell culture supernatant of VSV-SARS-CoV-2 infected human PBMCs (A) and the cytokine/chemokine profiles within the serum of VSV-SARS-CoV-2 vaccinated NHP (B). Serum samples or cell culture supernatant samples were prepared following the manufacturer’s instructions (Milliplex MAP kit, Millipore) and 25 μl of undiluted samples was added to 25 μl of assay buffer. Then, 25 μl of magnetic beads coated with specific antibodies (HT17MG-14K-PX25 combined with HIL18MAG-66K, Milliplex MAP Kit, Millipore) was added to this solution and the reaction was incubated at 4°C for 16 h. Next, the beads were washed and incubated with 25 μl of biotinylated detection antibody at room temperature for 1 hour. To complete the reaction 25 μl of Streptavidin–Phycoerythrin conjugate compound was added and allowed to incubate at room temperature for 30 minutes. The beads were then washed and incubated with 50 μl of sheath fluid at room temperature for 5 minutes. The samples were analyzed on MAGPIX instruments. The concentration of the analytes was then determined by MAGPIX xPONENT software. The assays were run in duplicate to confirm the results. Analytes were normalized to total protein concentration. Twenty six analytes were studied: interleukin 1 beta (IL-1β), interleukin-2 (IL-2), interleukin-4 (IL-4), interleukin-5 (IL-5), interleukin-6 (IL-6), interleukin-9 (IL-9), interleukin-10 (IL-10), interleukin-12p70 (IL-12p70), interleukin-13 (IL-13), interleukin-15 (IL-15), interleukin-17A (IL-17A), interleukin-17E/ interleukin-25 (IL-17E/25), interleukin-17F (IL-17F), interleukin-18 (IL-18), interleukin-21 (IL-21), interleukin-22 (IL-22), interleukin-23 (IL-23), interleukin-27 (IL-27), interleukin-28A (IL-28A), interleukin-31 (IL-31), interleukin-33 (IL-33), granulocyte-macrophage colony-stimulating factor (GM-CSF), interferon gamma (IFNγ), macrophage inflammatory protein 3 alpha (MIP-3α), tumor necrosis fact [file ppat.1010092.s015.tif]

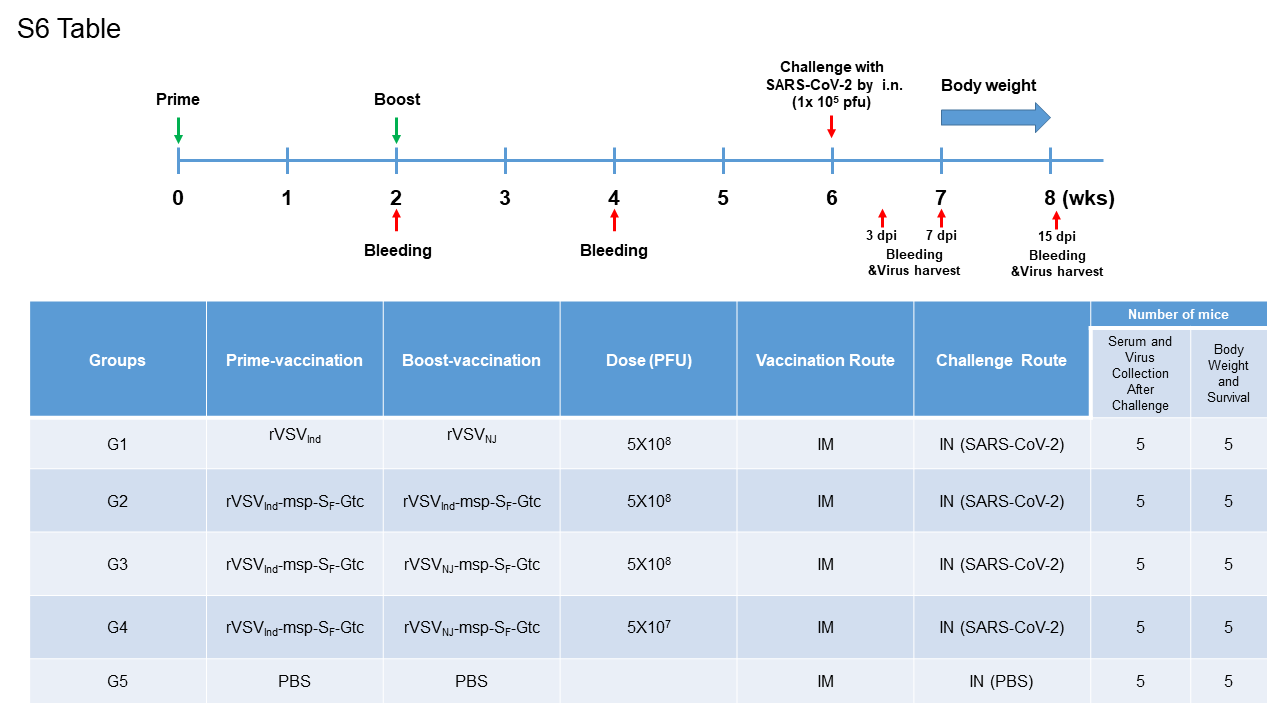

Supplement: S6 Table — Six-week-old female hACE2 transgenic mice (n = 5 per group) were prime-immunized with rVSVInd-msp-SF-Gtc and boost-immunized with rVSVInd-msp-SF-Gtc or rVSVNJ-msp-SF-Gtc two weeks after prime-immunization. Four weeks after boost-immunization, mice were challenged intranasally with 1x10^5^ PFU of SARS-CoV-2 (S clade, National Culture Collection for Pathogens (NCCP) #43326 Korea Disease Control and Prevention Agency) in a 50 μl volume intranasally under anesthesia. The survival and body weight of each mouse was monitored daily. (TIF) [file ppat.1010092.s016.tif]

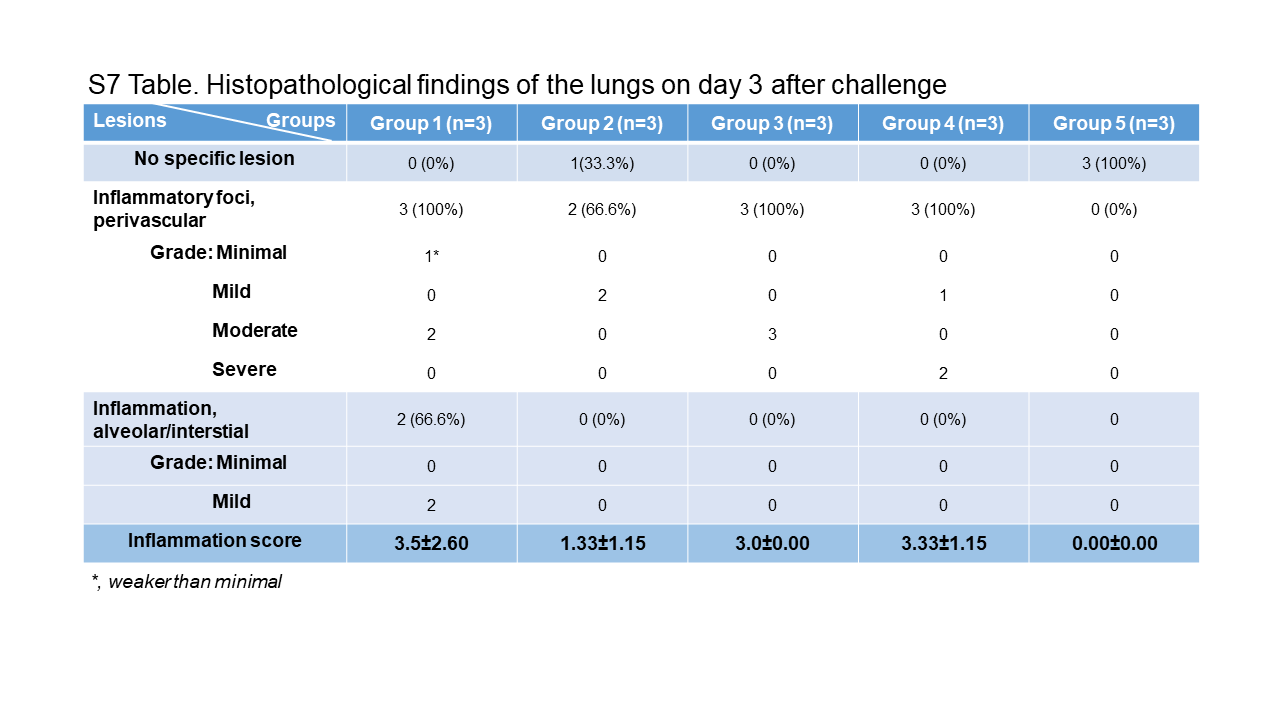

Supplement: S7 Table — Lesions in the lung tissues were graded semi-quantitatively depending on their severity. SARS-CoV-2 infection-related inflammatory lesions included inflammatory foci, characterized by infiltration of inflammatory cells around blood vessels and interstitial alveolar inflammation. In the cases of inflammatory foci, we graded inflammation based on the following criteria: 1) cases with very weak cell infiltration, close to normal, but there is recognizable inflammatory cell infiltration around some vessels (inflammatory score = 0.5), 2) some blood vessels with mild perivascular inflammatory cell infiltration in the lung section (minimal, inflammatory score = 1), 3) some blood vessels in the lung section with prominent infiltration of inflammatory cells (mild, inflammatory score = 2), 4) more than 50% of blood vessels in the lung section have prominent perivascular infiltration of inflammatory cells (moderate, inflammatory score = 3), 5) most blood vessels in the lung section have prominent and heavy perivascular infiltration of inflammatory cells (severe, Inflammatory score = 4). We scored the level of inflammation in each case by combining the two different inflammation scores of inflammatory foci and alveolitis. We then calculated the mean inflammatory score of each group from the individual inflammatory scores. (TIF) [file ppat.1010092.s017.tif]

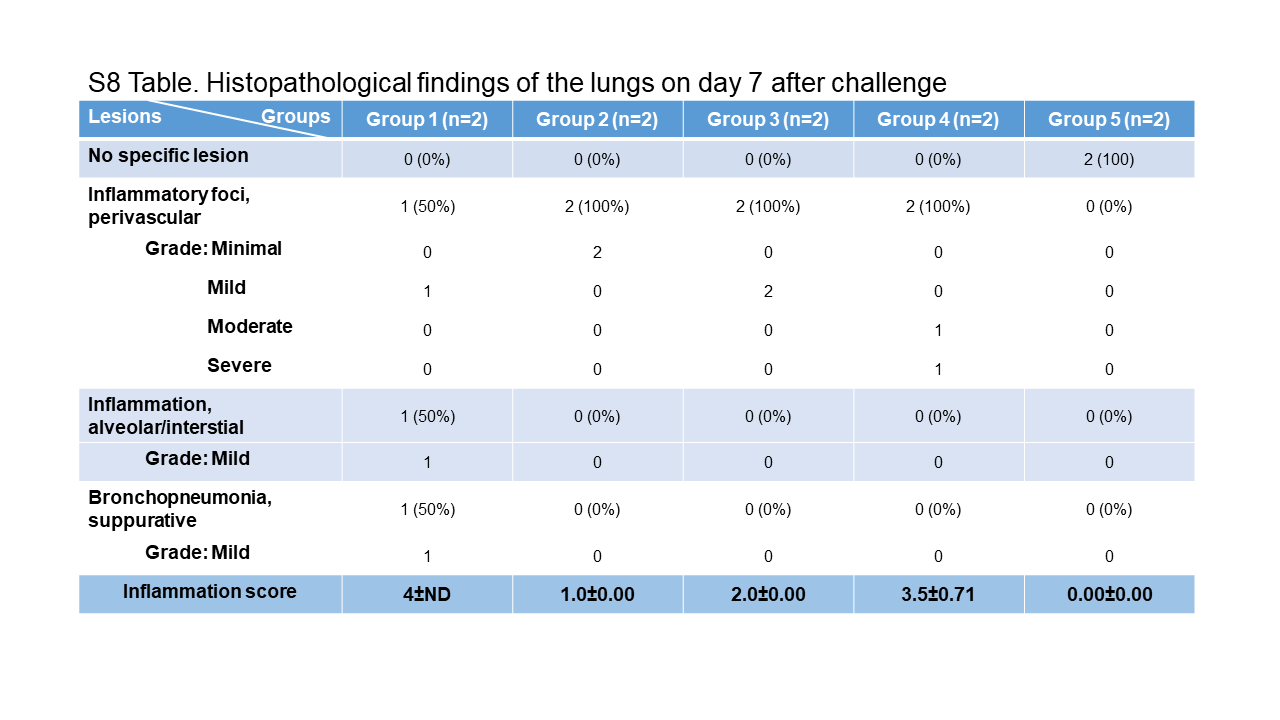

Supplement: S8 Table — Lesions in the lung tissues were graded semi-quantitatively depending on their severity. SARS-CoV-2 infection-related inflammatory lesions included inflammatory foci, characterized by infiltration of inflammatory cells around blood vessels and interstitial alveolar inflammation. In the cases of inflammatory foci, we graded inflammation based on the following criteria: 1) cases with very weak cell infiltration, close to normal, but there is recognizable inflammatory cell infiltration around some vessels (inflammatory score = 0.5), 2) some blood vessels with mild perivascular inflammatory cell infiltration in the lung section (minimal, inflammatory score = 1), 3) some blood vessels in the lung section with prominent infiltration of inflammatory cells (mild, inflammatory score = 2), 4) more than 50% of blood vessels in the lung section have prominent perivascular infiltration of inflammatory cells (moderate, inflammatory score = 3), 5) most blood vessels in the lung section have prominent and heavy perivascular infiltration of inflammatory cells (severe, Inflammatory score = 4). We scored the level of inflammation in each case by combining the two different inflammation scores of inflammatory foci and alveolitis. We then calculated the mean inflammatory score of each group from the individual inflammatory scores. (TIF) [file ppat.1010092.s018.tif]
